# Natural compounds-based nanomedicines for cancer treatment: Future directions and challenges

**DOI:** 10.1007/s13346-024-01649-z

**Published:** 2024-07-13

**Authors:** Tatiana Andreani, Ruoyu Cheng, Khalil Elbadri, Claudio Ferro, Thacilla Menezes, Mayara R. dos Santos, Carlos M. Pereira, Hélder A. Santos

**Affiliations:** 1 Chemistry Research Centre (CIQUP) and Institute of Molecular Sciences (IMS), Department of Chemistry and Biochemistry, Faculty of Sciences of University of Porto, Rua Do Campo Alegre s/n, 4169-007 Porto, Portugal; 2 GreenUPorto-Sustainable Agrifood Production Research Centre & Inov4Agro, Department of Biology, Faculty of Sciences of University of Porto, Rua Campo Alegre s/n, 4169-007 Porto, Portugal; 3 Drug Research Program, Division of Pharmaceutical Chemistry and Technology, Faculty of Pharmacy, University of Helsinki, FI-00014 Helsinki, Finland; 4 Department of Biomaterials and Biomedical Technology, The Personalized Medicine Research Institute Groningen (PRECISION), University Medical Center Groningen, University of Groningen, 9713 AV Groningen, The Netherlands; 5 Research Institute for Medicines, iMed.Ulisboa, Faculty of Pharmacy, Universidade de Lisboa, 1649-003 Lisbon, Portugal

**Keywords:** Biological compounds, Nanocarriers, Cancer cells, Drug delivery and targeting, Clinical trials

## Abstract

Several efforts have been extensively accomplished for the amelioration of the cancer treatments using different types of new drugs and less invasives therapies in comparison with the traditional therapeutic modalities, which are widely associated with numerous drawbacks, such as drug resistance, non-selectivity and high costs, restraining their clinical response. The application of natural compounds for the prevention and treatment of different cancer cells has attracted significant attention from the pharmaceuticals and scientific communities over the past decades. Although the use of nanotechnology in cancer therapy is still in the preliminary stages, the application of nanotherapeutics has demonstrated to decrease the various limitations related to the use of natural compounds, such as physical/chemical instability, poor aqueous solubility, and low bioavailability. Despite the nanotechnology has emerged as a promise to improve the bioavailability of the natural compounds, there are still limited clinical trials performed for their application with various challenges required for the pre-clinical and clinical trials, such as production at an industrial level, assurance of nanotherapeutics long-term stability, physiological barriers and safety and regulatory issues. This review highlights the most recent advances in the nanocarriers for natural compounds secreted from plants, bacteria, fungi, and marine organisms, as well as their role on cell signaling pathways for anticancer treatments. Additionally, the clinical status and the main challenges regarding the natural compounds loaded in nanocarriers for clinical applications were also discussed.

**Graphical abstract:**

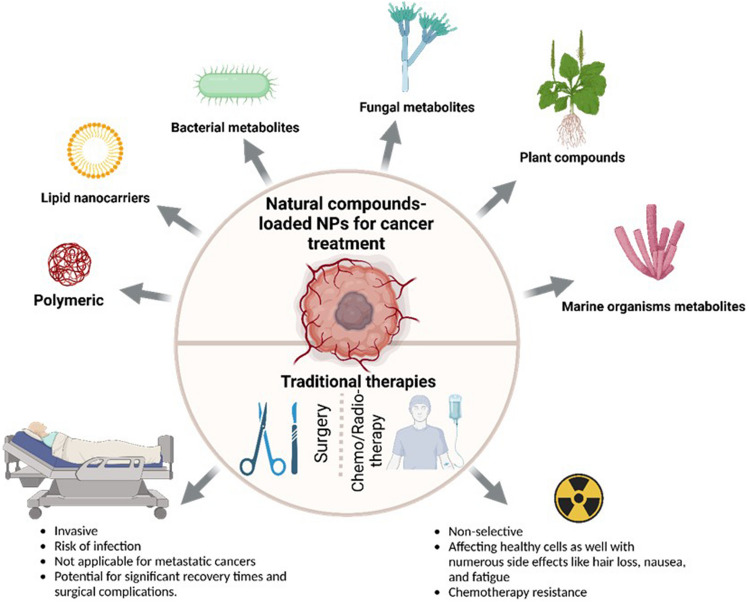

## Introduction

Cancer is a global public health condition affecting several aspects of person’s well-being leading to a decrease of the life expectancy all over the world [1]. Despite the prominent advances in cancer treatments, according to the estimative from World Health Organization (WHO), in the next two decades, the incidence rate of new cancer cases and the number of cancer deaths are expected to increase approximately 50 and 70%, respectively, worldwide [2] being breast, lung, colon, rectum and prostate are the most common type of cancers. In general, cancer comprises plenty of diseases which are attributed to an abnormal cell division leading to rapid and uncontrollable cell growth [3]. Although, genetic mutations play a pivotal role in altering the normal pathway of cell proliferation and differentiation, the physiological mechanisms of cancerous cell formation can also be driven by external factors, including, tobacco and alcohol consumption [4], obesity [5], poor sleep quality and physical inactivity [6]. However, despite the lifestyle behaviors can prevent the cancer risk, remarkable efforts have been performed for the improvement of the cancer treatments with different types of new drugs and less invasives therapies in comparison with the traditional modalities (radiotherapy, chemotherapy, immunotherapy and surgery), which are widely associated with long-term side effects, drug resistance, non-selectivity and high costs, limiting their clinical response [7, 8].

Given the chemotherapeutic properties toward different cancers, in the past decades, the use of natural compounds has played a powerful role for the development of several natural anticancer products for cancer prevention and treatment like paclitaxel, vincristine, vinblastine, camptothecin and irinotecan from herbal sources, actinomycin and doxorubicin from bacterial origins and bleomycin from marine organisms. However, although the natural products exhibit remarkable effects toward different cancer cells, the efficacy of these agents is limited due to their physical/chemical instability, poor aqueous solubility, and low bioavailability level [9]. In this sense, although the advent of nanotechnology in cancer therapeutics is recent, the application of nanoformulations containing natural agents has emerged as a promise strategy to circumvent the several shortcomings associated not only with the low absorption of natural compounds, as well as with the current cancer therapeutical modalities. Several nanomaterials have anticancer properties per se, whereas others are defined as nanocarriers in order to target and deliver anticancer compounds to cancer cells. Therefore, the use of nanocarriers can be considered as eligible candidates for the encapsulation of compounds from natural origin, protecting them from premature degradation, enhancing their aqueous solubility, delivering them directly to the tumor, improving the pharmacological response and reducing the side effects [10–12]. However, most of the formulations regarding the natural compounds loaded nanocarriers for cancer treatment are still in preliminary stages of development. The application of nanoformulations for cancer therapy faces various challenges required for the pre-clinical and clinical trials, such as technical and physiological aspects, as well as safety issues.

## Relevant strategies for anticancer agents delivery using nanomaterials (NM)

### Polymeric nanocarriers

Polymeric nanocarriers are polymer-based nanostructures that can physically encapsulate or trap various types of bioactive molecules. In this context, polymeric nanocarriers have been widely designed to potentiate the cancer treatments, serving as promising host systems for anticancer drugs due to their remarkable properties, including, homogeneous size distribution, high loading capacities, improvement of drug release and target, high drug circulation times and great stability in vivo [13–15]. Natural chemotherapeutic agents delivered by polymeric nanocarriers have showed significant enhancement in their therapeutic efficacy by site-specific targeting, as well as reduction of their toxic side effects [16, 17]. Polymeric nanocarriers are usually based on biodegradable polymers, which provide greater biocompatibility with tissues and organs and lower toxicity [18, 19].

Polymers can be synthetic or natural. Synthetic polymers are more reproducible, higher purity, easily manufactured and produce a continuous release of the active compounds over time. Synthetic polymers include aliphatic polyesters, such as poly(glycolic acid) (PGA), poly lactide-co-glycolide (PLGA), poly(3-caprolactan) (PCL) and polylactic acid (PLA), polymers based on poly(ethylene glycol)-PEG, aliphatic polycarbonates, polyphosphoesters (PPE), poly(acrylic/acrylates) like poly(methyl methacrylate) (PMMA), poly(acrylic acid) (PAA) and poly(methacrylic acid) (PAMA) [20–23]. Natural polymers present relatively fast release profiles and require additional purification steps before their use. However, they are recently gaining interest, since they are abundant in nature, inexpensive, show greater biocompatibility, high-water holding capacity and in general, they are prepared by mild methods (ionic gelation, coacervation, or complex complexation) [16, 24, 25]. Examples of natural polymers are polysaccharides such as chitosan, dextrin and pectin, proteins like silk fibroin and collagen, amino acids, such as arginine, peptides and polyesters like polyhydroxyalkanoates (PHAs) [25, 26]. According to their structural features, major polymeric nanocarriers used for delivery of natural cytotoxic drugs are polymeric nanoparticles (PNs), such as nanospheres and nano capsules, polymeric micelles (PMs), and dendrimers. Table 1 summarizes some studies allied to the use of polymeric nanocarriers for cancer treatment.

**Table 1:** Polymeric nanocarriers loaded with different biological compounds, their physicochemical properties and effect on cancer target

Type of Polymeric nanocarriers	Biological compound(s)	Physicochemical properties	Type(s) of cancer	Doses of the biological compounds applied on cancer target	Results on cancer target	References
Nanoparticles composed by zein protein and chitosan	Curcumin (CUR) and berberine (BER)	Particle size: 106.97 to 168.24 nm; Zeta potential (ZP): + 21.56 to + 36.76 mV	Breast cancer Lung cancer	Cell viability: CUR: 2.05 to 8.2 μg/mL BER: 1.1 to 4.3 μg/mL Cellular uptake: 8 μg/mL CUR and 4.2 μg /mL BER; Apoptosis and Enzyme-linked immunosorbent assay (ELISA): 4.1 μg /mL CUR and 2.2 μg /mL BER	Acquired higher cytotoxicity on cancer cells; Higher cellular uptake; Apoptosis and inhibition of IL-8 inflammatory cytokine secretion	[26]
Nanoparticles composed by Eudragit S100 (ES100) and polylactic-co-glycolic acid (PLGA)	CUR	Particle size: 120.58 ± 0.07 nm; ZP: -36.92 ± 0.2 mV	Colon cancer	Cytotoxicity: 0.001 to 10 µg/ml; Flow cytometric analysis: 10 µg/ml	Reduction of cell viability, enhanced specificity and higher cytotoxicity as compared to free drug	[17]
Nanoparticles composed by methoxy polyethylene glycol and poly (ε–caprolactone) (mPEG-PCL)	Doxorubicin (DOX)	Particle size: 38 to 135.1 nm; ZP: -3.6 to -5.8 mV	Malignant glioma	10 µg/mouse	Great circulation effects, increased tumor cell sensitivity and inhibition of tumor growth	[27]
Nanoparticles composed by chitosan-coated PLGA	Silibinin (SB)	Particle size: 284 ± 0.47 nm; ZP: + 22.5 ± 0.78 mV	Lung Cancer	In vitro cytotoxicity: 5 μg/mL; In vivo studies: 2 μg/mL	High antiproliferative effect and great cytotoxicity against A549 cells; Anticancer activity and enhanced cell death; Improvement of SB bioavailability	[28]
Nanoparticles composed by polylactic-co-glycolic acid and poly- ethylene glycol (PLGA-PEG)	Metformin (MET) and SB	Particle size: 235 ± 8.38 nm; ZP: ‒2.9 ± 1.2 mV	Lung cancer	SB: 0 to 100 μmol/L MET: 0–25 mmol/L	Great cytotoxicity against lung carcinoma cells (IC50 = 0.85 μM); Induction of cell apoptosis by upregulation of caspase-3, caspase-7 and Bax and via decreasing the expression level of cycling D1, Bcl-2 and hTERT mRNA gene	[29]
Nanoparticles composed by mPEG-PCL	DOX and Noscapine (NOS)	Particle size: 101 ± 4.80 nm; ZP: − 15.40 ± 1 mV	Breast Cancer	In vitro Cell Viability Assay: NOS: 10 to 1000 μmol/Lol/L and DOX: 0.10 to 10 μM. In vivo: DOX: 2 mg/kg and NOS: 50 mg/kg	Improvement of anticancer effect, synergistic behavior and lower cytotoxic effects; Tumor growth inhibition effect, antiangiogenic and apoptotic effects	[21]
Nanoparticles composed by mPEG-PCL	Thymoquinone (TQ)	Particle size: 117 ± 4 nm; ZP –10.6 ± 2.6 mV	Breast Cancer, epithelioid and colon carcinoma	In vitro TQ at concentrations equivalent to 3.12 to 200 µmol/L. In vivo: Pharmacokinetic Study: 6 mg/kg	Great effective in killing cancer cells, exhibited superior anticancer selectivity compared, enhanced cancer cell selectivity and exhibited low toxicity to normal cells; Enhancement in bioavailability, increased solubility of TQ	[23]
Micelles composed by hydrophilic poly(γ-glutamic acid) (γ-PGA) and α-tocopherol (Vitamin E)	CUR	Particle size: 50 – 70 nm	Breast Cancer	In vitro: Cellular Uptake Study: 30 µg/mL. Cytotoxicity Assay: 2.3 to 600 µg/mL. Multicellular Tumor Spheroid Assay: from 3 to 100 µg/mL. MitoTracker Assay and Angiogenesis Assay: 10–30 µg/mL. Wound-Healing Assay: 0–30 µg/mL In vivo assays: 0–60 mg/L	Inhibited the growth of the breast cancer cells, induced cellular apoptosis and showed strong angiogenesis inhibition activity; Embryonic malformation (Zebrafish) rates were significantly increased	[30]
Micelles composed by Polysorbate 80	CUR	Particle size: 17.59 ± 0.90 to 21.47 ± 2.68 nm; ZP:—3.31 ± 0.77 to – 3.27 ± 0.29 mV	Breast cancer	In vitro assays: Cellular uptake: 10 µmol/L; Cytocidal effects: 0–4 µg/mL In vivo assays: 4 mg/mL	High cellular uptake and high level of cytotoxicity; Significant reduction in tumor size and increase in bio-vailability/effectiveness due to nano formulation	[31]
Micelles composed by methoxy poly(ethylene gly- col)-b-poly(allyl glycidyl ether)-b-poly(ε-caprolactone (mPEG-b-PAGE(MPA)-b-PCL)	DOX	Particle size: 50.75 to 122.4 nm; ZP: − 21.5 to − 3.51 mV	Breast cancer	0.01 to 10 µg/mL	Readily cellular uptake, increased the endocytosis rate, decreased cytotoxicity and inhibited cancer cells growth	[32]
Micelles composed by PEG	DOX	Particle size: 27 nm	Osteosarcoma	In vitro assays: 0.5 to 20.0 µg/mL In vivo assays: 1 mg/mL	Enhanced cellular uptake, better antitumor effect; Observed obvious cancer cell atrophy, chromatin condensation, and great increase in osteosarcoma cell apoptosis	[33]
Micelles composed by 1,2-distearoyl-sn-glycero-3-phosphoethanol-amine-N-[methoxy(Polyethyeleneglycol)-2000] (DSPE-mPEG2000)	DOX	Particle size: 12.8 ± 0.4 to 13.2 ± 0.6 nm; ZP: − 3.1 ± 0.6 to − 2.4 ± 0.5 mV	Breast cancer	0.625–10 μmol/L	Micelles loaded with DOX were able to inhibit tumor growth, promoted tumor accumulation and exhibited antitumor efficacy	[18]
Micelles composed by (polyethylene glycol-block-poly[(1,4-butanediol)-diacrylate-β-N,N-diisopropylethylenediamine] (BDP)	SB and Docetaxel (DTX)	Particle size: 85.3 ± 0.4 nm	Breast cancer	In vitro assays: Cellular uptake: SB-DTX at 50 ng/well. Wound healing assay and in vitro migration and invasion assay: SB-DTX at 10 µg/mL. Cytotoxicity assay: SB: 2–200 µg/mL and DTX: 1–100 µg/mL In vivo assays: DTX: 4 mg/kg; SB: 8 mg/kg	Enhanced the cellular uptake of the drugs and good biocompatibility; Good suppression capability on primary tumor growth and the higher intracellular concentration	[34]
Dendrimers composed by PAMAM G4.0 dendrimer-ethylenedi-amine core (-NH2 terminated)	BER	Particle size: 52.8 ± 1.3 to 210.7 ± 9.98 nm; ZP: 30.3 ± 0.69 to + 30.6 ± 1.38 mV	Breast cancer	In vitro assays: 5–50 mg/mL In vivo assays: Hematological study and Pharmacokinetic studies: 500 µg/mL	Higher toxicity, high and significant anticancer potential, increased cellular uptake; Sustained release in vivo	[35]
Dendrimers composed by PAMAM G4.0	DOX	Particle size: 70 ± 1.68 to 106 ± 3.26 nm; ZP: 2.5 ± 1.6 to + 32.7 ± 1.98 mV	Breast Cancer	In vitro assays: 0.25 – 4.0 µg/mL In vivo assays: 10 mg/kg	Cellular uptake, increased cell death and enhanced permeability and retention (EPR) effect; Superior antitumor ability and limited side effects to normal organs	[36]
Dendrimers composed by PAMAM G4.0	CUR	Particle size: 10 – 120 nm; ZP: + 7 to + 35 mV	Glioblastoma	In vitro assays: 5 to 45 μmol/L In vivo assays: 0.5 mg CUR per 25 g of body weight	Cancer cell death and remarkably high apoptosis; Shrinked the flank tumors and preferential tumor accumulation	[37]
Dendrimers composed by Boc-Lys(Boc)–OH and Boc-Arg(Pbf)–OH amino acids	DOX and Gemcitabine (GEM)	Particle size: 5.82 to 6.05 nm; ZP: -1.25 to -7.72 mV	Pancreatic cancer	In vitro assays: Cellular uptake assay: DOX 2 – 10 μg/mL. Cytotoxicity assay: DOX 2 – 4 μg/mL In vivo assays: DOX biodistribution and tumor tissue penetration assays: DOX: 10 mg/mL. Antitumor assay: DOX: 5 mg/kg, GEM: 20 mg/kg	Higher cytotoxicity, long blood circulation and good biocompatibility; Enhanced the anticancer efficacy of either DOX or GEM	[38]
Dendrimers composed by PAMAM G3.0	DOX	Particle size: 37.8 to 142.8 nm; ZP: -29 to + 39 mV	Liver Cancer	1.25 – 40 μg/mL	Promoted high uptake efficiency, a more effective eradication of cancer cells, lower tumor growth and cancer regression	[39]
Dendrimers composed by PAMAM G4.5	Sorafenib (SOR) and Paclitaxel (PAX)	Particle size: 132.5 ± 1.8 to 152.6 ± 2.8 nm; ZP: -19.6 ± 2.4 to + 143.8 ± 3.2 mV	Liver cancer	In vitro assays: PAX: 0.01 – 50 μg/mL and SOR:0.06 – 300 μg/mL In vivo assays*:* PAX: 7.5 mg/kg and SOR: 45 mg/kg	Promoted the intracellular accumulation, enhanced cellular uptake, half-generation of PAMAM G4.5 dendrimers decreased the cytotoxicity of the carrier itself and improve the anti-tumor effect, enhancing the cellular concentration; Inhibited the increase of tumor volume, excellent tumor suppression and ensured a high tumor targetability	[40]

**Fig. 1:**
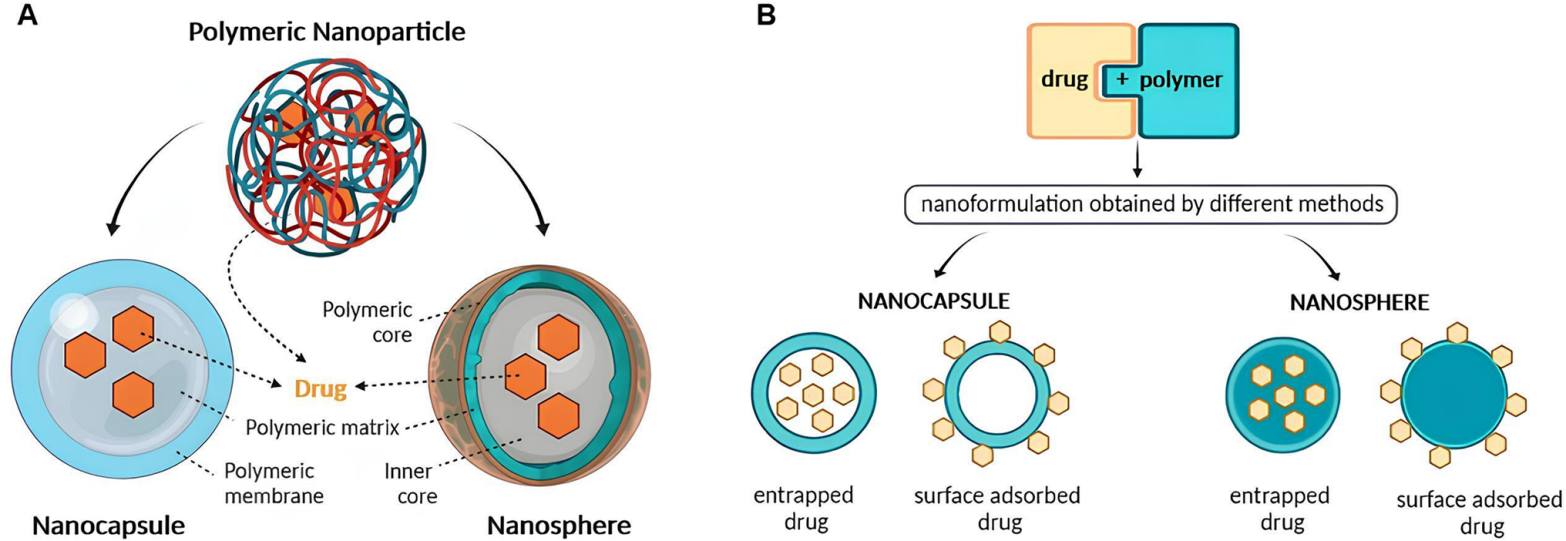
Types of structural forms of polymeric NPs: **A** Schematic depiction illustrating the structure of nanocapsules and nanospheres. **B** Various options for drug association with nanospheres and nanocapsules [41, 43]. This article is an open access article distributed under the terms and conditions of the Creative Commons Attribution (CC BY) license (https://creativecommons.org/licenses/by/4.0/)

PNs are widely used in nanomedicine for their drug delivery capacity. PNs refer to nano-sized spherical or irregular biodegradable colloidal systems. The antineoplastic drugs can be, in case of nanospheres, physically entrapped within cross-linked polymer or regarding nano capsules chemotherapeutic agents are entrapped in a cavity and surrounded by a polymeric membrane (Fig. 1). In both cases, the chemotherapeutic agent can also be conjugated to the surface or core of polymer NPs  [41, 42].

PNs have small sizes, mainly ranging from 1 nm to 1000 and have shown superior therapeutic efficacy by improving the bioavailability and pharmacokinetic profile of cytotoxic drugs, such as ursolic acid, curcumin and berberine, when compared to non-encapsulated anticancer drugs [26, 43, 44]. In addition to improve the solubility of cytotoxic agents, PNs also offer: a good cellular uptake [45], an increased residence time [28, 29], a more controlled drug release profile [46, 47] (and in some cases, it is possible to avoid burst effect [48]), a site-specific delivery [17], and a higher cytotoxicity in comparison to non-nanoencapsulated drugs [17, 29, 47]. Furthermore, PNs can provide desirable particle size. Small-sized NPs offer longer blood circulation time and as a result, a better delivery of cytotoxic drugs to the tumor tissue is observed [27].

Polymeric micelles (PMs) have attracted increasing attention from the scientific community as novel drug delivery systems for the treatment of diverse types of cancer. PM nanosized self-assembled core–shell structures that are formed by the self-association of amphiphilic di-block, tri-block or grafted copolymers in aqueous solutions. The spheroidal micellar structures are made up of two separated functional segments: outer shell and inner core. The term PMs are generally associated to systems where the hydrophilic part of the amphiphilic polymer is the outer shell, and the lipophilic part is directed to the core of the micelles. The hydrophilic shell provides stability to the NPs, allows the conjugation of hydrophilic drugs, and controls the in vivo pharmacokinetic behavior, while the hydrophobic polymer core can hold hydrophobic anticancer drugs and control the drug release behavior [49–51].

**Fig. 2:**
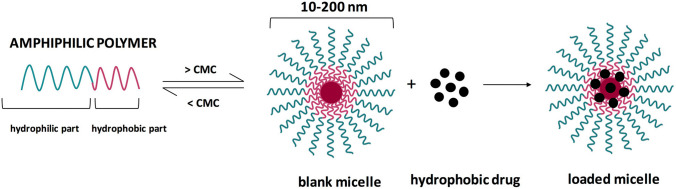
Process of formation of a polymeric micelle [56]. This is an open access article distributed under the terms of the Creative Commons CC-BY license, which permits unrestricted use, distribution, and reproduction in any medium, provided the original work is properly cited (https://creativecommons.org/licenses/by/4.0/)

**Fig. 3:**
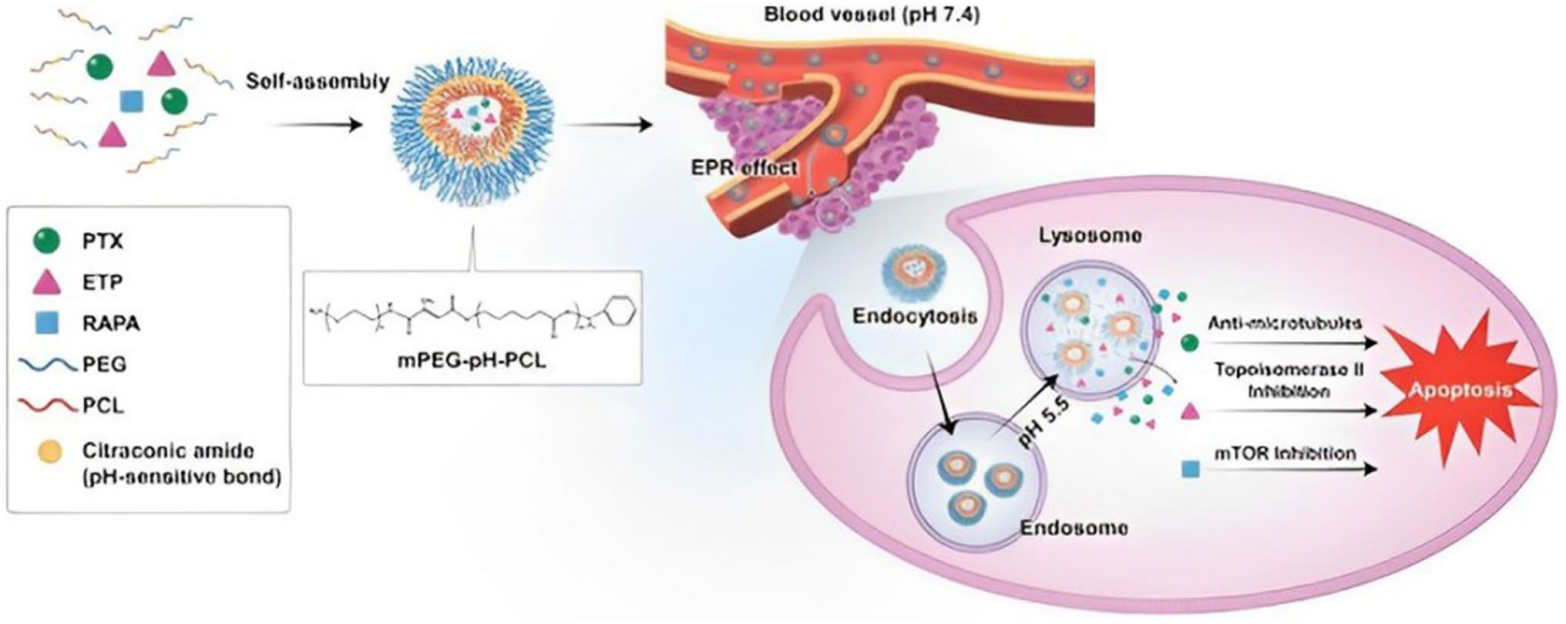
Schematic illustration of pH-sensitive mechanisms for effective tumor-targeted drug delivery, generating enhanced cellular uptake, and facilitating intracellular drug release [55, 57]. This is an open access article distributed under the terms of the Creative Commons Attribution (CC BY-NC) license (https://creativecommons.org/licenses/by-nc/4.0/)

In general, nanomaterials that are less than 10 nm can extravasate from normal blood vessel walls and from renal excretion, while those larger than 100 nm can be rapidly eliminated by the liver and spleen clearance. The size of the micelle ranges from 20 to 100 nm, providing several advantages. For example, their suitable size can accommodate a high number of anticancer drugs, generates long circulation times of the drug in blood that could result in enhanced bioavailability and decreased dosage and improves their drug delivery into the tumors with the advantage of lowering of the risk of nonspecific organ toxicity [49, 52]. Their unique structure results in the capacity of bypass the biological barriers and promote the drug accumulation in tumors via the enhanced permeability and retention (EPR) effect [33, 53]. Furthermore, PMs which have low critical micellar concentration (CMC) values present great tumor-specific accumulation, as well as having their micellar stability enhanced [15, 18, 20]. A potential strategy to increase the therapeutic efficacy and reduce side effects of cytotoxic drugs is to couple a specific stimuli-sensitive drug release mechanism with the delivery systems [15, 18]. Among all approaches, pH-sensitive micelles have been extensively studied and have demonstrated the capability of improving the efficiency of cancer treatment. The microenvironment of most solid tumors is intrinsically acidic (pH 6.5), while the physiological pH and normal tissue is about 7.4. The pH-sensitive micelles release the encapsulated cytotoxic drug at the tumor site after the degradation induced by acidic pH [54, 55] as illustrated in (Figs. 2 and 3).

**Fig. 4:**
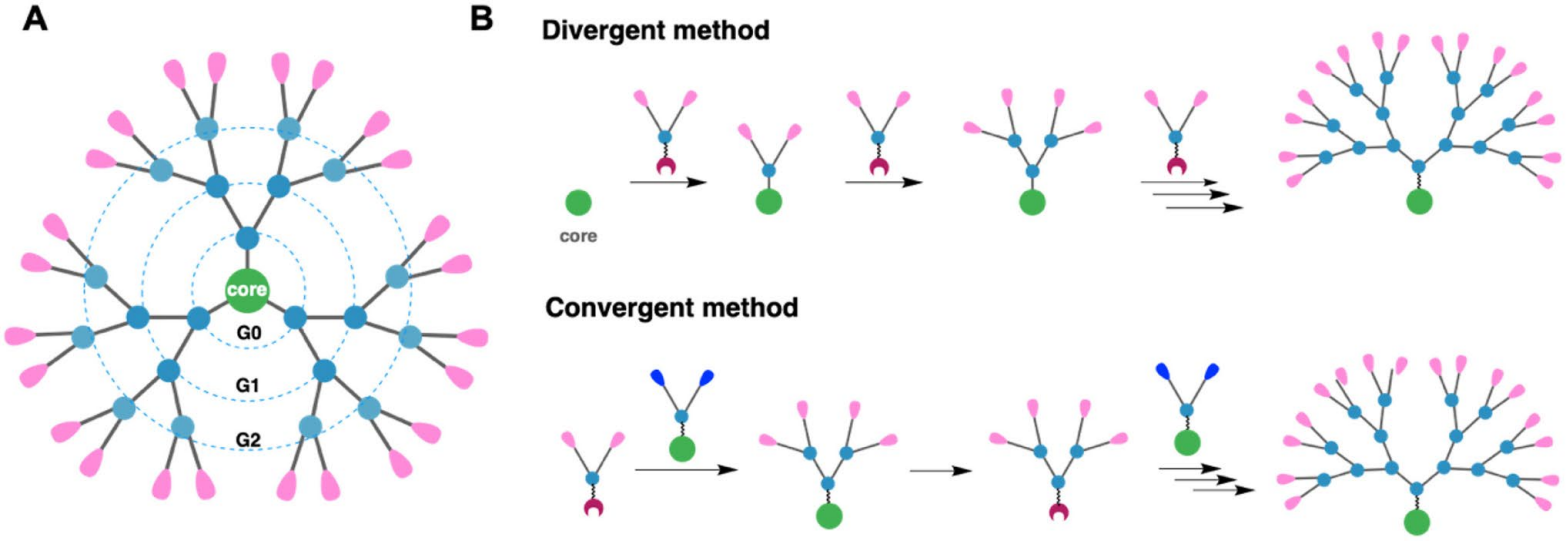
Diagram of a dendrimer structure and dendrimer synthesis. **A** General illustration of a dendrimer composed by a central core, radiating branch units defining the number of generations, and surface terminals. **B** Dendrimer synthesis via divergent and convergent approaches [61]. This is an open access article distributed under the terms of the Creative Commons CC-BY license, which permits unrestricted use, distribution, and reproduction in any medium, provided the original work is properly cited

Dendrimers are highly branched three-dimensional synthetic polymeric macromolecules and radially symmetrical. Which has a similar size to polymeric micelles. Dendrimers size ranges from 10 to 100 nm. They have three very well-defined structural regions: a central core, repetitive branching units covalently linked to the nucleus that determine the generation (G) of the dendrimer and finally, terminal groups that provide modifiable surfaces (Fig. 4). The high level of control over macromolecular growth and multifunctional surface structure of the dendrimers overcame other polymeric nanocarriers. In dendrimers, the antineoplastic drugs can be either encapsulated in their core or bound to their surfaces by covalent linkage or electrostatic interactions [58–60].

There are two major routes to synthesize dendrimers: the divergent method, developed by Tomalia et al. in 1985 [62], and the convergent method, introduced by Hawker & Frechet, 1990 [63] (Fig. 4). In the divergent method, the dendrimer is formed by the attachment of successive monomer layers to the dendrimer core. Moreover, the convergent method starts from the dendrimer outside (monomer) to the inside (core) [40, 64].

In drug delivery approach, dendrimers have high structural and chemical homogeneity, facilitating pharmacokinetic reproducibility. They can improve physicochemical features of cytotoxic drugs, such as stability, solubility, biodegradability as well as biocompatibility. In addition, they provide better pharmacological and pharmacokinetic properties, and minimized toxicity in normal cells by multiple functional groups in their surface [35, 36, 38, 40, 64–67]. Furthermore, they have selectivity for biological target, which increases the nanocarriers specificity along with stimuli-sensitive groups which triggers the dendrimer a controlled degradation allowing the anticancer drugs to be released in reaction to minor changes in the environment [37, 38, 40, 65, 68]. Apart from these encouraging characteristics, dendrimer suffer from high cost of production due to their multistep synthesis which can limit their extensive employment.

### Lipid nanocarriers

Lipid nanocarriers are promising nanostructures to deliver poorly water-soluble molecules, providing considerable protection of the active drugs from enzymatic degradation, higher stability, and bioavailability enhancement [69–71]. Lipid-based nanocarriers are composed of natural or synthetic lipids which can be (bio)degraded leading to a drug release in the controlled and targeted site. These systems are versatile nanostructures, easy to produce formulations, non-toxic and biocompatible [72–74]. Based on their physical and chemical properties, the main lipid-based nanocarriers are categorized into liposomes, solid lipid nanoparticles (SLNs) and nanostructured Lipid Carriers (NLCs).

Solid lipid nanoparticles (SLNs) are stable colloidal carriers introduced in 1990 as a potential attractive carrier system to substitute liposomes and polymeric nanoparticles due to their natural components [75]. SLNs are colloidal nanocarriers made from a mixture of solid lipids at both room and body temperatures stabilized by one or more surfactant agents.

SLNs encapsulate both lipophilic and hydrophilic drugs and are prepared from biocompatible and biodegradable lipids. Compared to liposomes, SLNs present the advantage of not employ organic solvents, reducing toxicity and are feasible for large-scale production at low cost [76, 77].

SLNs possess several characteristics that make them interesting for drug delivery systems, such as improvement in stability of the natural product coupled with enhanced cytotoxic activity, particularly against cancer cells [70, 78] and the increase bioavailability of poorly soluble drugs [79, 80]. SLNs enable controlled drug release (sustained or fast) [81], in addition to enhance the drug uptake when compared to the natural bioactive unconjugated [69, 82]. However, SLNs suffer from the high-water content of dispersion, lower drug loading capacity and drug expulsion on storage due to their rigid structure. To solve the drawbacks associated with SLNs, was developed the second generation of SLNs, the so-called nanostructured lipid carriers (NLCs) [83].

The innovation brought by NLCs consists in the replacement the solid lipids by solid/liquid two-phase hybridized lipid matrix varying in a ratio of liquid and solid lipids of 70:30 up to a ratio of 99.9:0.1, and the particles are stabilized by adding 0.5–5% surfactant agents [77]. The blend of solid and liquid lipids leads to the formation of disordered structures with more imperfections enabling a high drug loading. Other advantages of NLCs over SLNs are less inclined to unexpected gelation due to the presence of less water content, control release properties, and extend chemical stability upon storage. Regardless of the presence of liquid lipids in a high proportion, the NLCs are solid at room temperature and body temperatures [84, 85].

**Fig. 5:**
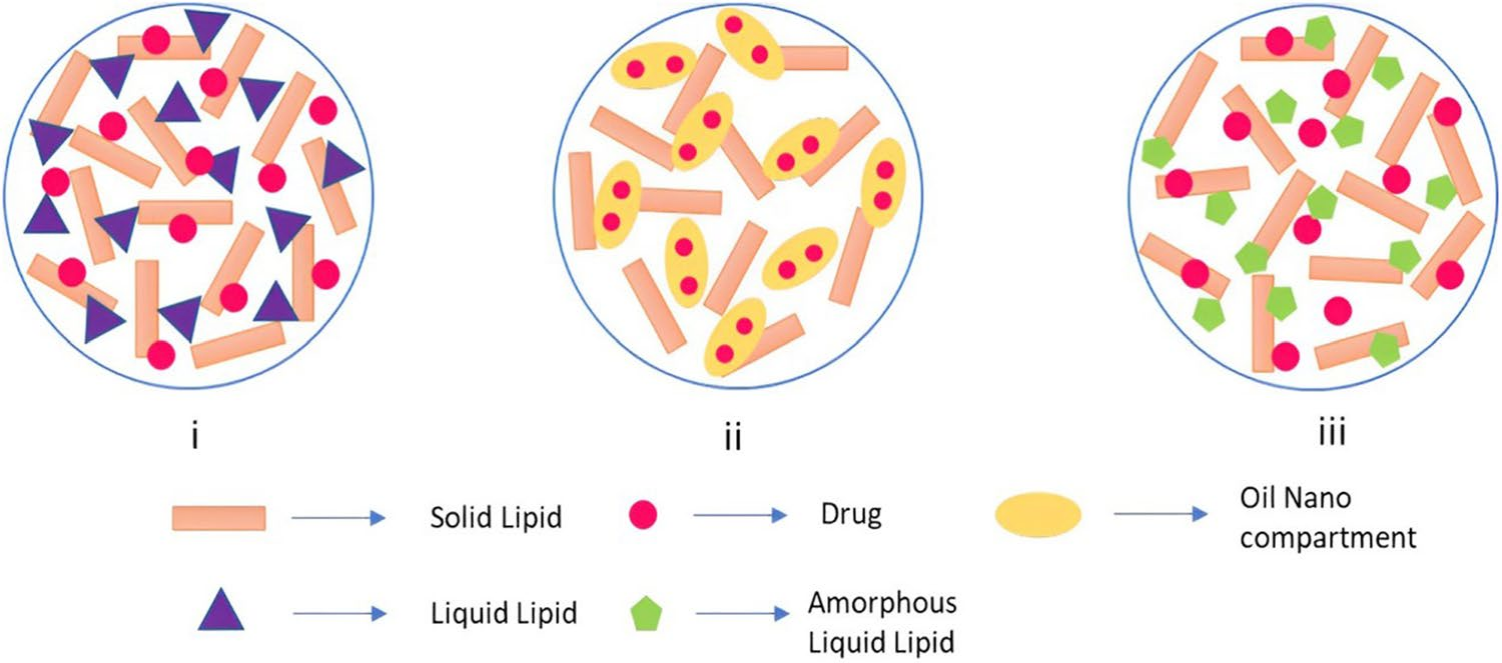
Different types of NLCs: **A** Type I: imperfect, **B** Type II: multiple and **C** Type III: amorphous [84]. This is an open access article distributed under the terms of the Creative Commons CC BY license, which permits unrestricted use, distribution, and reproduction in any medium, provided the original work is properly cited

NLCs can be classified in three different ways: Type I, Type II and Type III (Fig. 5). In NLCs Type I, also known as imperfect crystal, a small amount of the solid lipid is replaced by liquid lipids, increasing the ratio of imperfections in the structure. This extra free space to integrate drugs leads to a higher loading capacity.

In NLCs Type II, also known as the multiple types, the solid lipid is mixed with higher amounts of oil. As a result, there is a formation of oily nano-compartments within the solid lipid matrix and a better control release of the drug from the matrix is observed.

Finally, the NLCs Type III, the central matrix is solid, but the central core remains amorphous. This technique avoids the crystallinity in the matrix, preventing drug leakage, besides allowing the drug-controlled release [77, 84, 86].

**Fig. 6:**
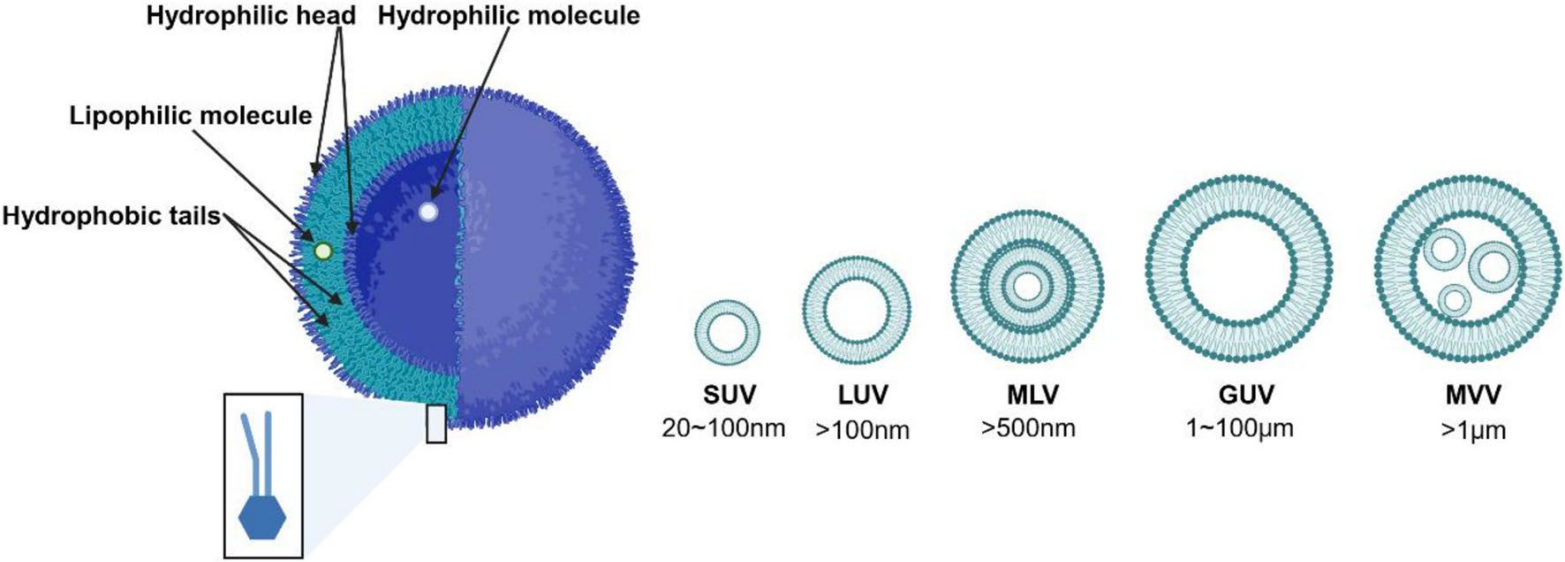
General liposome structure and classification based on layer number and size [92]. This is an open access article distributed under the terms of the Creative Commons CC-BY license, which permits unrestricted use, distribution, and reproduction in any medium, provided the original work is properly cited

Liposomes are spherical self-assembled vesicles composed of an aqueous core surrounded by one or more phospholipid bilayer membranes. The characterization of liposomes is based on the structure of the lipid bilayers. In the large unilamellar vesicles (LUVs), ≥ 100 nm, and small unilamellar vesicles (SUVs), < 100 nm, the vesicles have a single phospholipid bilayer surrounding an aqueous core. LUVs and SVUs are better suitable for encapsulating hydrophilic compounds [87–90], while the multilamellar vesicles (MLVs) are composed of multiple lipid bilayers creating a multilamellar vesicle of concentric phospholipid spheres, wherein each lipid bilayer is separated by the aqueous medium. The MLVs present sizes from a few hundred nanometers to several micrometers and are usually employed to encapsulate lipophilic drugs due to possible incorporation within each lipid bilayer. Another form of liposome is multivesicular vesicles (MVVs). The MVVS are composed by several SUVs surrounded by a single phospholipid bilayer [72, 87, 89, 91]. Figure 6 shows the main types of liposome structures.

Liposome-based lipid nano systems are attractive candidates for encapsulation and controlled delivery of cytotoxic compounds applied in cancer treatment. Studies showed that natural antineoplastic drugs liposome-encapsulated presented lower toxicity towards normal tissues and better anticancer activity in relation to the natural free drugs [25, 93–95]. In addition, it is observed a good stability in the blood circulation and improvement in pharmacokinetics and biodistribution of the natural drug [71, 91, 96]. Moreover, the physiochemical properties of liposomes can be easily modified to better control their size, change their surface charge, and target drug delivery to improve the antitumor effect of chemotherapeutics and reduce the harm of normal tissues [97–100].

Lipid-based nanocarriers, such as SLNs, NLCs and liposomes have been extensively explored for encapsulating/conjugating biological compounds for anticancer therapy as shown in Table 2.

**Table 2:** Lipid nanocarriers loaded with different biological compounds and their effect on cancer therapy

Type of Polymeric nano-carriers	Biological compound(s)	Physicochemical properties	Type(s) of cancer	Doses of the biological compounds applied on cancer target	Results on cancer target	References
SLNs composed by hydrogenated Soybean Phospholipids	CUR	Particle size: 101.4 ± 0.21 to 106.1 ± 0.45 nm ZP: + 26.8 to + 30.0 mV	Liver cancer, lung cancer and Hepatocellular carcinoma	Cytotoxicity assay: 6.25–100 μg/mL. Cell Apoptosis assay: 10–20 μg/mL	Inhibition of the proliferation of the cancer cells, prevention of invasion and metastasis of malignant tumor cells, reduction of CUR toxicity to normal hepatocytes and showed a significant apoptotic effect	[81]
SLNs composed by stearic acid	CUR	Particle size: 218.5 ± 3.7 to 231.4 ± 2.5 nm ZP: − 8.36 ± 0.1 to − 8.11 ± 0.1 mV	Prostate cancer	In vitro assays: Cell viability test and Cellular uptake analysis by flow cytometry: 10 mg/mL. Apoptosis study: 25 – 100 µg/mL. Cellular uptake analysis using fluorescence microscopy: 50 μg/mL In vivo assays*:* 10 mg/kg	Strong anti-proliferative activity, high cellular uptake and improvement of apoptotic activity; Significant tumor regression	[69]
SLNs composed by trilaurin, cholesterol and stearoyl chitosan	CUR	-	Triple- Negative Breast Cancer (TNBC)	In vitro assays: Cytotoxicity: 5–50 µmol/L Rhodamine 123 Efflux, Reactive Oxygen Species and Nuclear Factor-kB and Hypoxia Inducible Factor-1α Activity: 5 mg/kg In vivo assays: 5 mg/kg	Loaded CUR increased DOX accumulation, increased intracellular retention of DOX and rhodamine 123 in cells and reduced the levels of Pgp mRNA; CUR-loaded SLNs decreased the rates of tumor growth and mass	[79]
SLNs composed by lecithin and glyceryl monostearate	PAX and CUR	Particle size:178.1 to 250 nm ZP: –48.4 to + 26.4 mV	Ehrlich ascites carcinoma	In vivo assays: Toxicity Study: PAX: 10–20 mg/kg Anticancer Study: PAX: 10 – 20 mg/kg, CUR: 10 – 20 mg/kg	Toxicity studies showed near-normal values. Anticancer study showed cellular blebbing and shrinkage, besides better anticancer property	[80]
SLNs composed by precirol, transcutol, and gelucire	Resveratrol (RES) and CUR	Particle size:100 to 200 nm ZP: − 37.48 ± 9.88 to –6.45 ± 5.33	Colorectal cancer	In vivo assays: RES and CUR: 0–10 µmol/L	Showed better percentage viability and anticancer activity	[101]
SLNs composed by cetyl alcohol	Papain	Particle size: 123.1 ± 3.41 to 126.9 ± 2.82 nm ZP: -27 ± 3 mV	Colorectal cancer	In vitro Cell Viability: 5 to 80 µg/mL	Cell viability of HT-29 was decreased significantly with high cytotoxicity	[82]
SLNs composed by stearic acid	Variabilin	Particle size: 198.2 ± 42.56 to 322.8 ± 36.02 nm ZP: − 31.4 ± 5.16 to − 18.3 ± 4.68	Breast, colon, and prostate cancer	Cell Culture and Cytotoxicity Studies: 1.56–50 µg/mL. Apoptosis Assays: 38.08 – 100 µmol/L	The study showed improvement of stability, a protective effect on the non-cancerous cells, increased the viability of the cells and induction of a more significant apoptotic effect	[70]
NLCs composed by octyl decyl acid triglycerate and lecithin	CUR	Particle siz: 99.99 ± 1.87 nm ZP: − 19.9 ± 0.65 mV	Hepatocellular carcinoma	In vitro assays: Apoptosis: 10 μmol/L. Determination DR4 and DR5: 5–20 μmol/L	Cur-NLC modulated DR5/caspase-8/-3 mediated extrinsic apoptosis pathway involved in HepG2 cell apoptosis	[102]
NLCs composed by Glyceryl monostearate	CUR and Temozolomide (TMZ)	Particle size: 78.49 ± 0.38 to 97.53 ± 0.56 nm ZP: -8.54 ± 0.51 to + 0.22 ± 0.01 mV	Glioma	In vitro: MTT assays: CUR: 5 μg/mL and TMZ: 10 μg/mL. Cell cycle and apoptosis analysis: CUR and TMZ: 10 μg/mL. Calculation of cooperativity between dual drugs: CUR and TMZ 3.33 and 6.67 μg/mL In vivo: CUR: 0.2 mg/kg and TMZ: 0.4 mg/kg	Stronger inhibitory effect on glioma cells, reduced cytotoxicity on normal cell, synergistic inhibition effect on C6 cell lines by inducing cell apoptosis; Improved synergistic anticancer effects of encapsulated dual drugs, inhibitory effect on the growth of tumor, enhanced synergistic treatment effect in vivo and a minimal toxicity on normal organs and tis sues in vivo	[103]
NLCs composed by cetyl palmitate, Gelucire® and oleic acid	DOX	Particle size: 185 ± 9 to 281 ± 18 nm ZP: − 30.0 ± 1.0 to –22.3 ± 0.6 mV	Breast cancer cells	In vitro assays: 0–1.7 μg/mL	Enhanced cytotoxicity and cellular uptake. Improvement the target therapeutic effectiveness in situ, while minimizing the well-known side-effects	[74]
NLCs composed by Arginyl-glycyl-aspartic acid	DOX and Sildenafil citrate (SIL)	Particle size: 70.2 ± 2.5 to 280.7 ± 3.9 nm ZP: -18.5 mV	Lung adenocarcinoma	In vitro assays: DOX: 0.5 – 8 μmol/L and SIL: 5 – 25 μmol/L	More accumulation effect and high induced apoptosis	[104]
NLCs composed by lecithin	Gambogic acid (GA)	Particle size: 19.19 ± 0.06 to 26.59 ± 0.10 nm ZP: − 7.64 + 0.47 to − 3.04 ± 0.49 mV	Breast cancer	In vitro assays: 0.1- 2.5 µg/mL In vivo assays: 2 mg/kg	Higher cytotoxicity and anti-proliferative effect, excellent biocompatibility; Great tumor growth inhibition and facilitated GA intracellular uptake	[105]
NLCs composed by myristyl myristate or illipe butter	S. *lycocarpu’*'s fruits alkaloidic extract	Particle size: 56.81 ± 1.1 to 72.2 ± 2.9 nm ZP: − 13.05 ± 2.52 to − 10.22 ± 0.65 mV	Bladder cancer	In vitro: Cytotoxicity Assays: 2.344 – 75 μg/mL. Flow Cytometry: 12.6 μg/mL	Time-dependent cytotoxic effect, antitumor activity in bladder cancer cells and high anti-proliferative activity	[106]
Liposomes composed by Cholesterol and hydrogenated soybean phospholipids	BER and Irinotecan (IRI)	Particle siz e: 164.8 ± 0.473 nm ZP: -17 ± 0.473 mV	Pancreatic cancer	In vitro assays: IRI and BER: 0.3–2.4 μg/mL In vivo* assays:* Pharmacokinetic and biodistribution: IRI: 4 mg/kg and BER 2 mg/kg. Anti‑cancer effect and safety estimate of lipBI: IRI: 6 mg/kg and BER 3 mg/kg	Strong dose-dependent inhibitory effect on BXPC-3 cells and no impact on normal cells; Significantly inhibition of tumor growth and reduction of the gastrointestinal toxicity	[107]
Liposomes composed by P90G and P100H, and cholesterol	BER and TAR	Particle size: 127.8 ± 29.6 to 230.3 ± 38.6 nm ZP: − 20.3 ± 2.5 mV	Leukemia	In vitro assays: DOX and TAR: 10 µmol/L	Induced cell apoptosis and accumulation in tumor cells	[108]
Liposomes composed by cholesterol and soybean phospholipid	CUR and Docetaxel (DTX)	Particle siz e: 208.53 ± 6.82 nm ZP: − 23.1 ± 2.1 mV	Breast cancer	In vitro: Cell viability assays: DTX: 25–200 g/mL and CUR: 12.5–100 g/mL In vivo assays: Pharmacokinetic studies: CUR: 1.0 mg/kg and DTX: 2.0 mg/kg. antitumor study: CUR: 0.1 mg/kg and DTX: 0.2 mg/kg	Low vitality and inhibition of MCF-7 cells proliferation;. Increased pharmacokinetic parameters, enhanced antitumor effect, reduced their exposure in non-targeted tissues and significantly inhibited tumor growth	[109]
Liposomes composed by multifunc- tional peptide probe (EMC) and DSPE − PEG_2000_ − Mal	DOX and Tariquidar (TAR)	Particle size: 111.80 to 136.18 nm ZP: − 13.7 ± 0.9 to − 7.8 ± 1.4 mV	TNBC	In vitro assays: Antitumor Efficacy: DOX and TAR: 0 to 156.25 μmol/L. Drug Uptake, Efflux and Penetration: DOX: 10 μmol/L In vivo assays: DOX and TAR: 5 mg/kg	Inhibition of tumor and extensive apoptosis; High local concentration of the chemotherapeutic agents in tumor cells, enhancement of the antitumor efficiency and reduction of the systemic toxicity	[99]
Liposomes composed by soy phosphatidylcholine and cholesterol	CUR	Particle size: 76.1 ± 12.9 to 91.6 ± 23.3 nm ZP: − 90.4 ± 50.6 to − 41.7 ± 4.3 mV	Hepatocellular carcinoma	In vitro: Cytotoxicity, Hepatic targeting and lysosomal targeting assays: 20 µmol/L In vivo: Targeting assay: 5 mg/kg. Antitumor effect and biosafety: 9.9 mg/kg	Increased the cellular uptake, great targeting effect and high biosafety; Enhanced antitumor effect, induced cell apoptosis with better therapeutic effect	[110]
Liposomes composed by soy lecithin and cholesterol	DOX	Particle size: 99 ± 2.33 to 115 ± 1.23 nm ZP: − 24.2 ± 2.08 to − 15.5 ± 0.25 mV	Hepatocellular carcinoma	In vitro assays: 6.3 – 1584.9 ng/mL In vivo assays: 5 mg/kg	Increased the cellular uptake, greater targeting effect, higher biosafety and enhanced antitumor effect; Drug accumulation in tumors, superior anti-tumor ability, with limited side effects	[111]

### Naturally derived nanovesicles

**Fig. 7:**
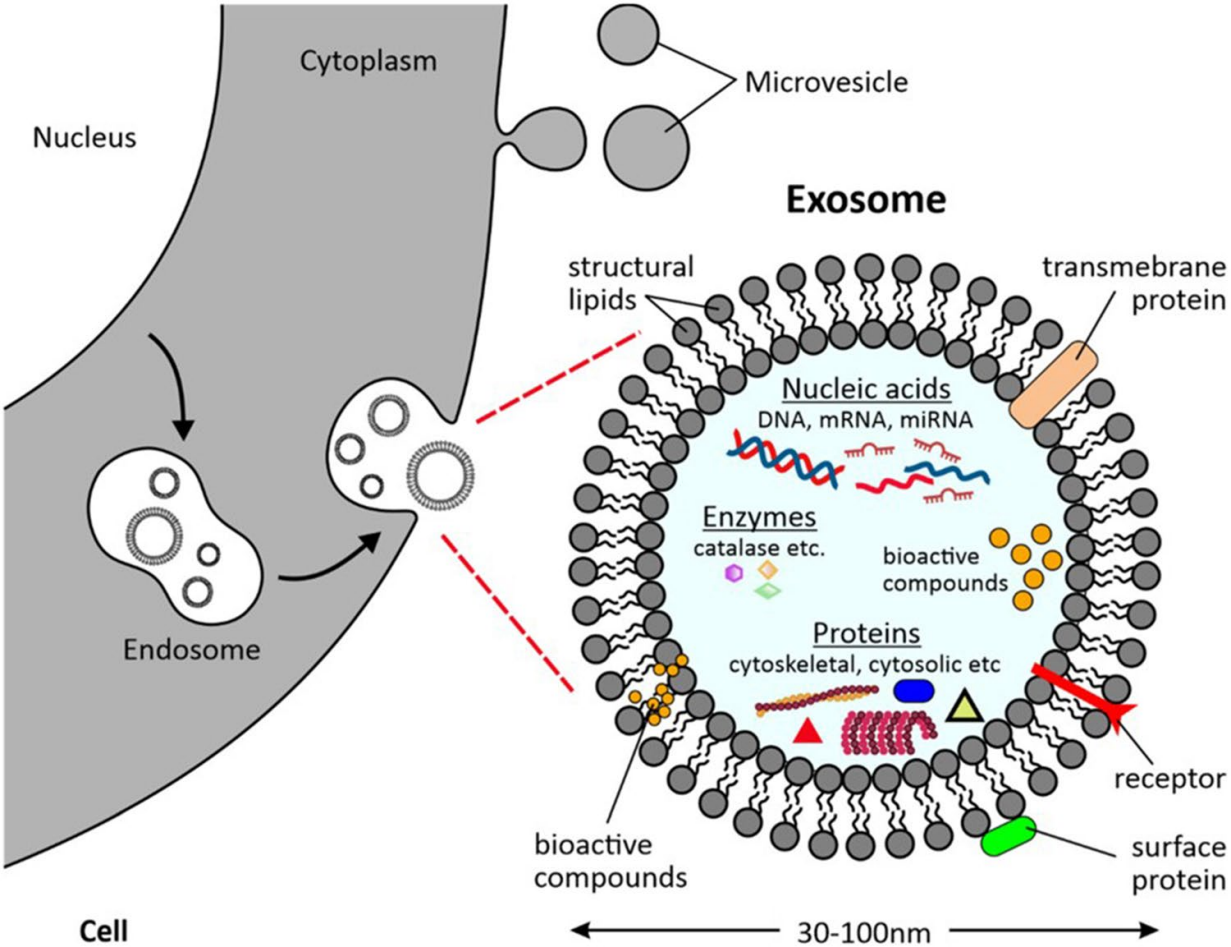
An illustration depicting the releases of a specific type of EV, namely an exosome [112]. The image presents several examples of bioactive compounds that can be incorporated in these EVs for delivery and diverse therapeutic applications. This is an open access article distributed under the terms of the Creative Commons CC-BY license, which permits unrestricted use, distribution, and reproduction in any medium, provided the original work is properly cited (https://creativecommons.org/licenses/by/4.0/)

Naturally derived nanovesicles, such as extracellular vesicles (EVs) and microvesicles, have also gained significant attention in recent years due to their potential in cancer treatment. These nanovesicles are derived from various cell types and are naturally occurring, making them an attractive alternative to synthetic nanosystems like liposomes. EVs and microvesicles are lipid bilayer-enclosed vesicles naturally released from cells (Fig. 7). EVs include exosomes (30–150 nm) and microvesicles (100–1000 nm), distinguished by their size and biogenesis pathways. EVs are formed within the endosomal system and released upon fusion of multivesicular bodies with the plasma membrane, while microvesicles bud directly from the plasma membrane. Both EVs and microvesicles carry a diverse array of biomolecules, including proteins, lipids, and nucleic acids, reflective of their parent cell's phenotype and state. This composition allows them to facilitate intercellular communication and modulate various biological processes, making them highly relevant for therapeutic applications.

The therapeutic potential of EVs and microvesicles in cancer treatment is multifaceted. One of their primary advantages is their ability to deliver therapeutic agents directly to cancer cells, exploiting their natural targeting capabilities and ability to cross biological barriers. Furthermore, their surface proteins can be modified to enhance targeting specificity, reducing off-target effects and improving therapeutic efficacy. Additionally, these vesicles can carry immunomodulatory molecules, potentially reprogramming the tumor microenvironment to enhance anti-tumor immune responses. EVs derived from immune cells can be used to boost the immune system's ability to recognize and attack cancer cells. Furthermore, the molecular cargo of EVs can serve as biomarkers for cancer diagnosis and progression monitoring, providing a non-invasive method for real-time assessment of treatment efficacy.

Naturally derived nanovesicles offer several advantages over synthetic nanocarriers, such as liposomes. Due to their natural origin, EVs and microvesicles are inherently biocompatible and exhibit low immunogenicity, reducing the risk of adverse immune reactions compared to synthetic nanocarriers like liposomes. EVs possess innate homing abilities, allowing for more precise delivery of therapeutic agents to specific tissues or cell types, which is often challenging with synthetic nanocarriers. Moreover, EVs can carry a complex array of therapeutic molecules simultaneously, including proteins, nucleic acids, and small molecules, offering a multifaceted approach to cancer treatment. Despite their potential, the use of EVs and microvesicles in clinical applications faces several challenges. One of the primary challenges in the clinical translation of EVs is the lack of standardized protocols for their isolation, purification, and characterization. Developing scalable and reproducible methods is crucial for their widespread clinical use. The heterogeneity of EVs in terms of size, composition, and functional properties poses a challenge for consistent therapeutic outcomes. Advanced sorting and characterization techniques are needed to ensure uniformity in EV-based therapies. The regulatory landscape for EV-based therapies is still evolving. Clear guidelines and stringent quality control measures must be established to ensure the safety and efficacy of these nanomedicines. Additionally, enhancing the targeting capabilities and therapeutic payloads of EVs through genetic and chemical modifications remains a key area of research. Innovations in this field could significantly improve the specificity and potency of EV-based cancer therapies.

### Passive and active targeting strategies for cancer therapy

#### Passive targeting

In healthy tissues, exchange of molecules smaller than 3 nm (*e.g.*, water, gases, salts, specific metabolites) across vasculature takes place mainly in capillaries, which are composed of a layer of endothelial cells and connective tissue, while molecules larger than 3 nm cannot pass through the vascular endothelium [113, 114]. However, solid cancers have a unique structure, which enables the accumulation of NPs delivery systems in a process defined as passive targeting [115, 116]. Passive targeting is mainly based on the tumor physiology and non-cellular components, such as the development of neo-vasculature from the pre-existing systems (angiogenesis) and defects in the lymphatic drainage in solid tumors that exceed 2 mm^3^ size, with increased tumor interstitial fluid pressure [117–121].

**Fig. 8:**
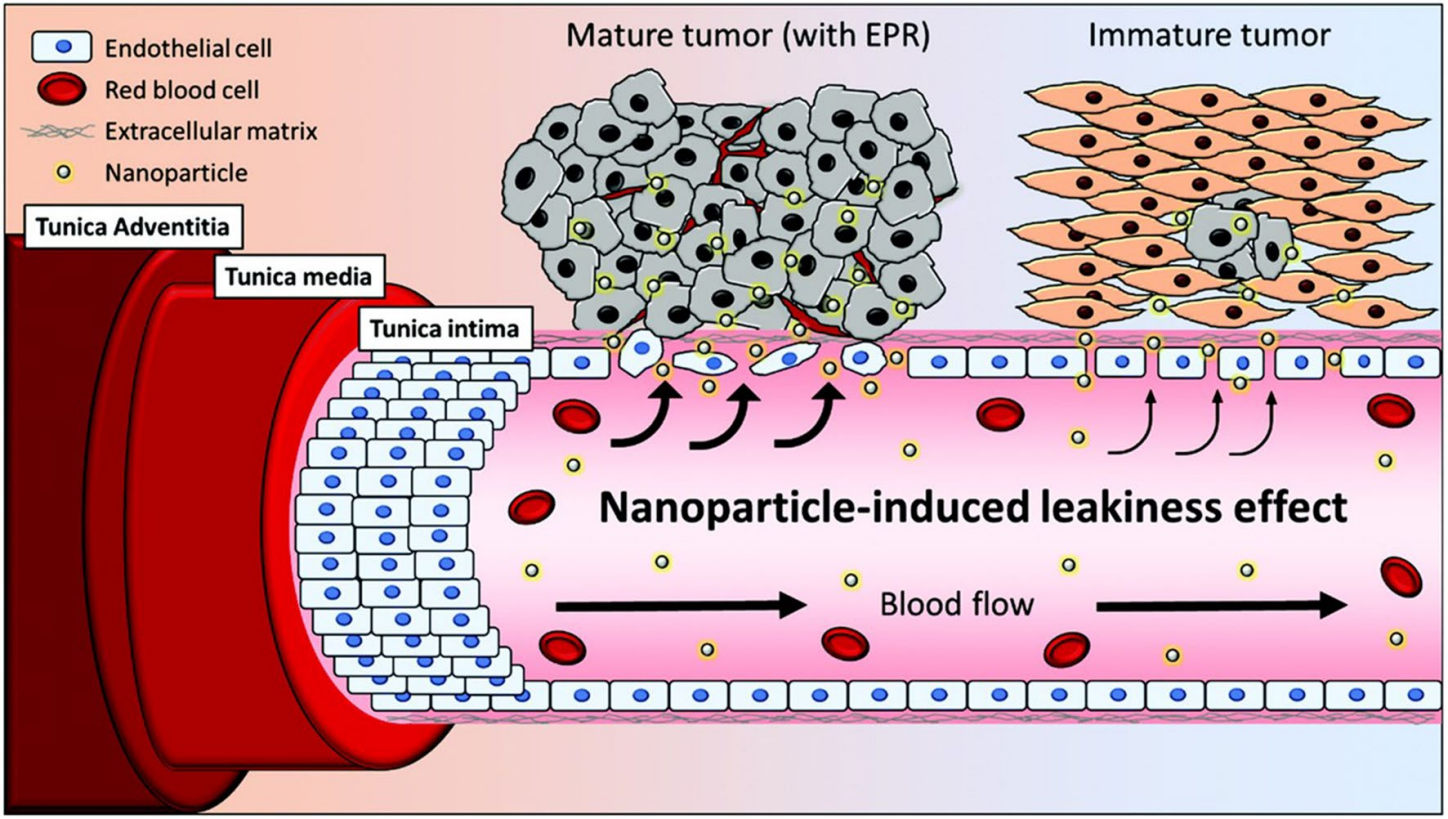
Differences between a healthy and endothelium surrounding a mature tumor. While endothelial cells of healthy tissues present are tightly joined while the tumor is still immature, the tumor vessel's structure contains wide fenestration that allows an EPR of NPs in the tumor interstitium. Adapted with permission [115]. Copyright 2015, Elsevier

The formation of aberrant tissue masses leads to inflammation and hypoxia, with consequent recruitment of new vessels [114]. These irregular blood vessels, with low pericyte count, proliferating endothelial cells that are weakly aligned with wide fenestrations and an irregular build of the basement membrane, facilitate the extravasation of circulating nanoparticles (Fig. 8) [114, 115, 118, 122, 123]. Furthermore, the increasing concentration of several molecules such as free radicals that affect the integrity of vascular endothelium, nitric oxide, and vascular permeability promoters (*e.g.*, bradykinin/kinin, prostaglandins, cytokines) also contributes to an enhanced permeability and retention (EPR). Therefore, NPs sizing 20–200 nm are can extravasate from the lumen of tumor blood vessels into the tumor interstitium through interendothelial gaps, in a transport mechanism defined as paracellular route, usually via passive diffusion [113, 115–118, 120, 124], proving a reasonable tumor specificity with a delivery 20–30% higher than normal organs, decreasing the therapeutics side effects [114].

The EPR effect is also highly affected by the NPs physiochemical properties, such as size, shape/morphology, surface charge and chemistry, which can be modified by altering the NPs material or the fabrication method [118, 122]. For example, the NPs small size increases their circulation time, penetration, and accumulation into growing tumor interstitium, which results in higher the drug availability and timespan [114, 116]. The reduced NPs size also increases the inter-particular friction and surface area, leading to higher chemical reactivity and antitumor efficacy [116]. However, the range of NPs used for cancer therapy tends to be narrow, ranging from 30 and 100 nm. NPs with less than 10 nm are cleared out by the kidney, and bigger than 100 nm can be removed via the reticuloendothelial system in the spleen and liver [116, 118, 123, 125]. Besides, NPs tend to be immediately opsonized and removed from the blood by the mononuclear phagocytic system (MPS) after IV administration [115, 116, 120]. For that reason, PEGylation of NPs has been used to reduce their rapid clearance and therefore increase their blood circulation time and the accumulation in the tumor microenvironment (TME) [114, 115]. Additionally, NPs shape plays an important role in their distribution and cell uptake, being that rod-shaped NPs have higher absorption, followed by spherical, cylindrical, and cubic NPs [122]. NPs surface charge also plays a significant role in their internalization [126]. Positively charged NPs are internalized by cancer cells rapidly and strongly than the negatively charged ones [117, 122, 127]. On the other side, the positive charged NPs are more prone to interact to blood components, such as proteins, lipids, and sugars, which forms a “protein corona” around the NPs surface that affects their cellular internalization. This “protein corona” is divided into two sections, the hard and the soft corona. The hard corona contains proteins with high affinity and that are tightly bound to the NPs surface, being more stable than the soft corona, which is more dynamic and unstable and is constituted by proteins with low affinity towards the NPs surface [120, 128]. This “protein corona” gives the NPs new biological properties that define their physiological response [120]. Also, positively charged particles are more prone to bind to opsonins and be cleared by macrophages, or to be removed by the liver, resulting in decreased pharmacological activity [118, 120, 122, 123], while neutral or negatively charged NPs are considered ideal to avoid the formation of a protein corona [129].

Other passive targeting strategies that have been also studied are based on the tumor-induced hypoxia, adenosine nucleoside accumulation and acidic pH (5) [117]. Indeed, while the constant physical and chemical stress withing the TME increases the amount of ATPs release by the activated immune cells and the malignant cells, an oxygen-deficient environment changes the metabolic pathways of tumor cells to anaerobic, producing pyruvate and then to lactic acid instead of CO_2_ (5) [117]. Therefore, while the extracellular pH of normal healthy tissues and blood ranges between 7.2–7.5, the tumors present a low extracellular pH around 6.4 to 7 [114, 117, 118]. Materials with acid-cleavable bonds that degrade and release their cargo in lysosomes or in the acidic tumor tissue are often used as a stimulus for NPs responsive drug delivery systems [127].

**Fig. 9:**
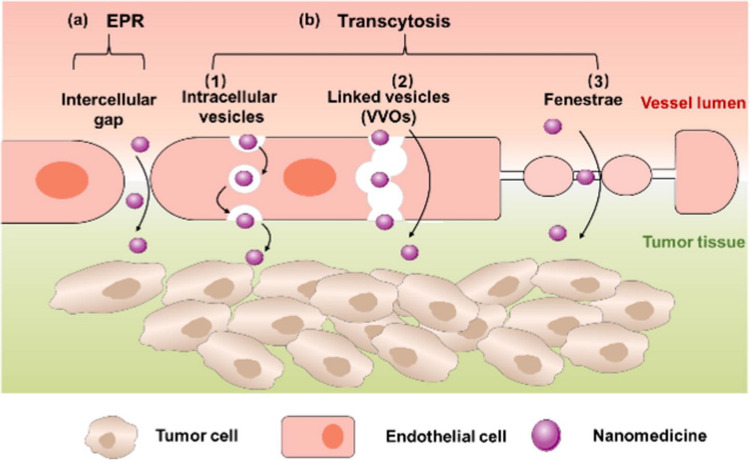
NPs from the blood circulation into the tumor interstitium by the EPR effect (**a**) or transcytosis (**b**), which can be subcategorized as intracellular vesicles-mediated (1), vesiculo-vacuolar organelles (VVOs)-mediated (2) or fenestrae-mediated (3) pathways. Reproduced with permission [130]. Copyright 2022, Elsevier B.V

However, the EPR effect is very heterogenic, being highly variable across tumor angiogenesis, lymphangiogenesis, and size (large tumors present a weak EPR effect), type of cancer, patients, and their individual lesions, leading to highly unpredictable and low therapeutic efficiency [114–116, 123, 130]. Smaller tumors are usually very vascular, with a more homogenous EPR effect than larger ones (2–3 cm in diameter in mice), in which the blood vessels are obstructed by coagulation or thrombogenic system activation, with formation of necrotic areas where is impossible for NPs to reach [114, 115]. Also, while hepatocellular and renal carcinoma have abundant vascularization, pancreatic and prostatic cancers present low levels of blood vessels [115]. So, despite the efforts made to explore the EPR effect within the TME, the NPs delivery efficiency and clinical results are still limited [115]. A new approach for NPs passive delivery, based on active trans-endothelial delivery have been studied [115]. Transcellular transcytosis is an active process in which macromolecules are transported across the cell interior to actively extravasate into solid tumors. The transcytosis transport pathways can be intracellular vesicles, vesiculo-vacuolar organelles (VVOs), and fenestrae (Fig. 9). Transcytosis through intracellular vesicles involves 3 steps: apical endocytosis, intracellular transportation, and basolateral exocytosis. Vesiculo-vacuolar organelles (VVOs) enable macromolecules transcytosis by connecting the blood-facing lumen and the extravascular interstitium and are especially active in tumor endothelial when compared with healthy tissues. Fenestrations are gaps between endothelial cells, which occurrence is increased in vascular permeability factor VEGF. Since transcytosis does not depend on the EPR effect, it is less susceptible to cell-to-cell variation [130]. Besides, the gaps between the endothelial cells only made up about 0.048% of the blood vessel surface area, while the tumor vasculature is abundant in the structures involved in transcytosis [115]. Therefore, transcytosis of tumor endothelial cells and tumor cells have demonstrated an increment of NPs penetration in solid tumors by active extravasation, accumulation, without relying on the EPR effect [115, 130].

#### Active targeting

Since it is also reported that passive targeting is only effective in rodents and not in humans [131], another approach to increase the NPs anticancer efficacy is to decorate their surface with ligands by either chemical conjugation or physical adsorption [129]. The functionalization with ligands that identity and bind to superficial receptors overexpressed in the tumor cells is described as active targeting [115, 116, 118, 131, 132]. This targeting strategy not only improves anticancer therapeutic effects, but also decreases the side effects of the drug loaded into the NPs [118, 133, 134]. Several ligands have been used for NPs active targeting like proteins, aptamers, antibodies, nucleic acids, protein/peptides, carbohydrates (*e.g.*, glucose and cellulose) and other small molecules, which can mediate the attaching and increase their accumulation within the tumor cells via receptor-mediated endocytosis [113, 117, 118, 135].

There are several active targeting approaches, such as angiogenesis mediated targeting, receptor-mediated targeting and peptide targeting [117, 123]. The angiogenesis mediated targets are not tumor cells, but the adjacent endothelial cells, involved in the formation of neovasculature and the downregulation of the immune system, by destroying the basement membrane and interstitial matrix [117, 129]. Gene mutation and hypoxia increases hypoxia-inducible factor (HIF-1α) and matrix metalloproteinase (MMP), leading to the overexpression of endothelial-derived growth factor (VEGF), which main function is angiogenesis and increased vascular permeability [131]. For this reason, the main angiogenesis mediated targets are VEGF and its receptor, vascular cell adhesion molecules (VCAM), *a*_*x*_*ß*_*y*_-integrins [117, 135] and MMPs [117, 131, 134], which inhibition using monoclonal antibodies like Mab-bevacizumab (marketed as Avastin), anti-VCAM-1 and M200/volociximab, respectively, have shown antineoplastic effects [117]. Other example is the F3 peptide, which targets the nucleolin receptor, expressed on endothelial cells produced during angiogenesis This active targeting results in lower tumor blood supply, reducing the apport of nutrients and oxygen and preventing poor drug delivery, being more adapted to tumor heterogenicity [114].

Receptor-mediated targeting is based on the decoration of NPs with ligands or antibodies, such as folic acid, transferrin and cetuximab or trastuzumab, that bind to receptors overexpressed in the tumor cell membranes like the folate receptor, the transferrin receptor, and the human epidermal growth factor receptor (EGFR), respectively [113, 114, 117, 124, 127, 135]. Since cancer cells require folic acid for their development and maintenance [129], folate receptors are overexpressed in several cancer types, such as ovary, breast, colon, lung, kidney, head, neck, brain and testicular. Furthermore, because folic acid has low molecular weight and immunogenicity, its use for NPs functionalization lowers the time necessary to reach the target, while increasing NPs penetration [131]. Also, the iron demand of cancer cells is much higher than healthy cells, which leads to the overexpression of transferrin receptors (2–10 times more than normal cells) [129, 135]. Another example of receptor-mediated targeting is the decoration of NPs with hyaluronic acid, that binds to CD44 [119, 122, 125], overexpressed in several cancer tissues, such as pancreatic, breast and lung [131]. Besides, several monoclonal antibodies (cetuximab, rituximab, trastuzumab, and bevacizumab) with demonstrated targeting ability have been approved by the FDA. However, since antibodies are complex and large compounds, the conjugated NPs can present drug delivery limitations. The NPs functionalization with antibody fragments proved to have potential for NPs functionalization, since they are smaller and lack the complement activation region of antibodies, while preserving their specificity towards the therapeutic target [136].

Peptide targeting examples are the tumor homing peptides (THPs) and the cell-penetrating peptides (CPPs). THPs are peptides that target both the tumor and its TME, consisting in 3–15 amino acid sequence motifs, such as RGD (Arg-Gly-Asp) and NGR (Asn-Gly-Arg), which binds to α integrins and a receptor aminopeptidase on the surface of endothelial cells, respectively [114, 117, 127, 131]. The CPPs have 5–30 AA and are hydrophobic, cationic, and amphipathic, being able to translocate through the plasma membrane, which is made of negatively charged lipidic bilayer [117, 118]. Chlorotoxin is another peptide used in active targeting. This 36-amino acid peptide, obtained from *Leiurus quinquestria* scorpion venom, targets glioma, breast cancer, prostate cancer, and neuroectodermal tumors (*e.g.*, neuroblastoma, melanoma, ganglioneuroma, pheochromocytoma, small cell lung carcinoma) [135]. Furthermore, glucose coating with has also shown to increase NPs uptake, since glucose transporter channel 1 is overexpressed in high metabolically active cells, such as cancer ones [135].

**Fig. 10:**
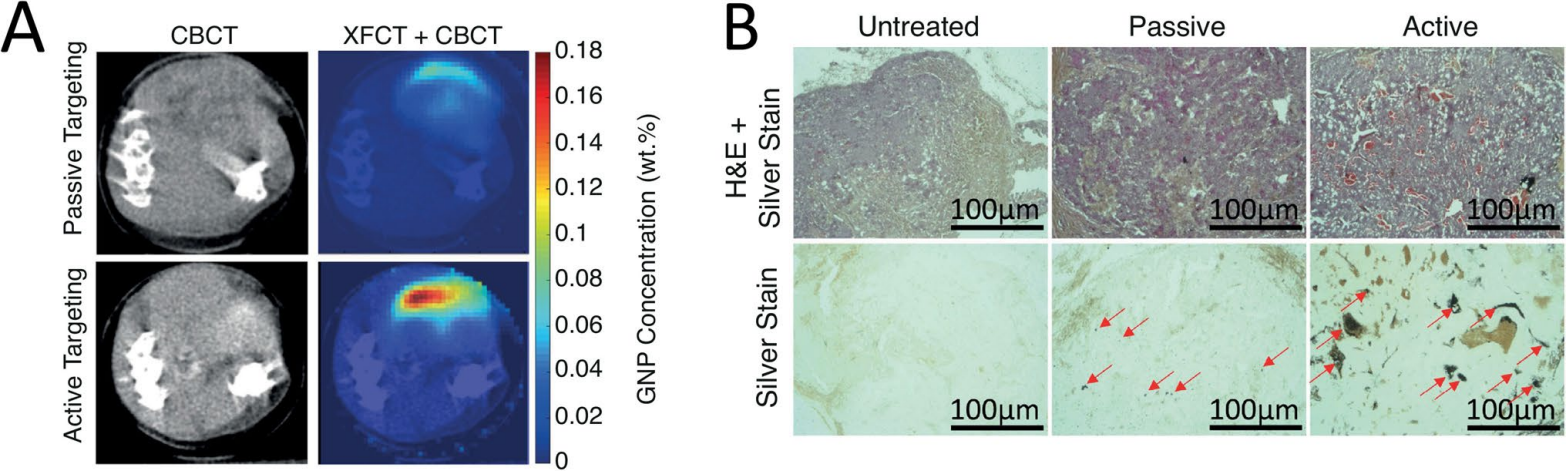
The active targeting of AuNPs using anti-PSMA antibodies has demonstrated higher penetration into the tumor site than non-functionalized AuNPs. **A** Dual-modality computed tomography (CT) and x-ray fluorescence CT (XFCT) transverse slices of 2 mice receiving passive and active targeting GNPs administrated IV demonstrated that the active targeting presented a maximum concentration 3 times higher than that of passive-targeting GNPs. **B** The H&E and silver staining demonstrated that GNPs (stained black; red arrows) presented a higher accumulation in the tumor site when they were functionalized with anti-PSMA antibodies, being possible to observe clusters of GNPs. Adapted with permission [137]. Copyright 2021, Elsevier B.V

Also, several ligands and their targeting receptors can be specific to the type of cancer. For example, pectin targets the asialoglycoprotein receptor, which is mostly expressed on the surface of hepatic cells [125, 136]. Also, antibodies have been used to target the prostate-specific membrane antigen (PSMA) (Fig. 10), a transmembrane protein overexpressed in advanced, metastatic, and hormone-refractory prostate cancers [119, 137]. Moreover, both the human epidermal growth factor receptor-2 (HER2) and the estrogen receptors, Erα and Erβ are overexpressed in several types of breast cancer, being also studied as targets for NPs functionalization [129].

However, even if NPs bind to the target cell receptor, it does not ensure they are successfully internalized into the cell, especially if the affinity of ligands is low or if the binding force between the ligands and their receptor is weak. Furthermore, drug efflux caused by p-glycoproteins contributes to multidrug resistance [122]. Currently, other active targeting approaches have been studied, such as use mannan, a biodegradable polysaccharide, to target mannose receptors expressed in the surface of antigen presenting cells (APC), with potential applications for vaccine delivery [135]. Also, stimuli-responsive NPs have also been demonstrated to selectively target cancer cells. These NPs are controlled by an external stimulus, such as magnetic field, ultrasound, radiofrequency ablation, microwaves, such forces NPs to concentrate on the cancer site [122, 125].

## Anticancer potential of natural compounds-loaded NM

### Bacterial metabolites-loaded NM

Natural compounds derived from bacterial secondary metabolites have been extensively studied in recent decades and used in the treatment of several types of cancer, due to their high therapeutic efficiency. Bacteria have evolved to produce various secondary metabolites with different structural and functional capabilities, and their produced bioactive compounds have been categorized as non-ribosomally synthesized products (antibiotics, non-ribosomal peptides, polyketides, and others) and ribosomally synthesized (small peptides and toxins). Most of these compounds have shown potential activity against cancer cells [138, 139].

The mechanisms of action for several compounds such as actinomycin D, bleomycins, carfilzomib, doxorubicin, ixabepilone, mitomycin C, taxol, pentostatin, rapamycin and romidepsin, among others, against cancer cells have been thoroughly discussed in detail in the literature [139–141]. Most of these secondary metabolites are now produced synthetically in the laboratory to increase of drug yield, minimize the toxicity effects by genetic modification, and especially to reduce of production costs facilitating the direct access to these therapeutic compounds [142]. However, despite their high potential in cancer treatment, these anticancer compounds have some drawbacks, including low solubility in aqueous media [143], low bioavailability [144], reduced duration in blood circulation, rapid enzymatic degradation, limitation of permeation across the cell membrane [145], as well as, inability to distinguish cancer cells from normal cells, resulting in life-threatening side effects [146] and significant general organ toxicity [147]. In this sense, several inorganic and organic nanomaterials have been developed and used as promising carriers for anticancer drug delivery due to their unique physicochemical and biological properties [148] to significantly enhance cancer therapy efficiency through better targeting, reduced systemic toxicity, regulation of therapeutic residence time in circulation and preservation of therapeutic bioavailability [149].

Inorganic NPs, including metallic, magnetic, carbon nanotubes, quantum dots and mesoporous silica have been selected as nanocarriers for different anticancer drugs due to many advantages, related to the fact that their physical and chemical properties are improved due to their small size, large surface area, effect of quantum size and electron configuration [150], which can be viable for biosensor applications, cell marking, redirection, image processing and detection, as well as, for therapeutical purposes [151]. However, the successful application of such NPs in pharmaceutical area is strictly dependent on surface modification by functionalization and/or bioconjugation, as inorganic NPs cannot overcome the cellular barriers, which in turns hampers the drug delivery to the site of action [152, 153]. Amongst the types of inorganic nanocarriers, it is possible to highlight a large amount of works which describe the association of biomolecules for cancer therapy to gold NPs. This fact is mainly related to the refractive indices of gold nanocarriers, which are among the most critical factors attracting them to health, facilitating the connection between gold NPs and biomolecules, such as enzymes, carbohydrates, fluorophores, peptides, proteins and DNA [151].

There is a notable interest for a specific secondary metabolite, Doxorubicin (Dox), which is a compound originally produced by the bacterium *Streptomyces peucetius*. Dox is approved by the FDA for cancer therapy, including breast cancer, ovarian cancer, Kaposi's sarcoma and lymphomas [154] and its mechanism of action is essentially based on DNA strand breakage, mitochondrial depletion, formation of the metabolite doxorubicinol mediated by free radicals [155]. However, the treatment associated to Dox presents some limitations, including life-threatening side effects, toxic effects on non-diseased cells, dose-limiting myelosuppression, and dilated cardiomyopathy leading to congestive heart failure [3, 156]. A recent study conducted by Zheng et al. [157] showed that gold NPs (AuNPs) loaded with cysteine alginate nanogel (ACA) and the cationic drug doxorubicin led to the formation of a multistage drug nanotransporter (Dox@ACA), which effectively inhibited the lung cancer cells growth. The electrostatic attractions between AuNPs, ACA and Dox and the photothermal effect of the AuNPs demonstrated that these NPs were significantly more efficient in A549 human lung adenocarcinoma cells (in vitro) and reduced considerably the hepatocellular tumor (in vivo) in comparison with each compound individually. Furthermore, since AuNPs can respond to external light stimuli and enhance tumor-target drug release, Dox@ACA did not induce significant effects on non-target cells/tissues in contrast to free Dox.

Although Dox is the most natural bacterial compound loaded NPs, other secondary metabolites like Bleomycin, Salinomycin, Exotoxin A, Listeriolysin O, Mitomicyn C and Carfilzomib have also been explored and conjugated with metallic NPs to overcome some disadvantages widely associated with the use of these compounds, such as suppression of bone marrow function [158], unfavorable bioavailability and pharmacokinetics [159], cumulative and dose-dependent pulmonary toxicity [160], cutaneous reactions [161], hematological malignancies [162], as well as, hair loss, nausea, vomiting [163], stomatitis and tissue necrosis [164]. Table 3 highlights the main studies on inorganic NPs for cancer therapy.

**Table 3:** Natural compounds of secondary metabolites from bacteria loaded inorganic nanoparticles, their physicochemical characterizations, and effects on cancer cell lines

Biological compounds	Bacterial source	Type of Nanomaterial	Nanoparticle properties	Target cell lines	Doses of biological compound applied on target cells	Effect on target cells	Reference
Doxorubicin	*Streptomyces* peucetius	Gold nanoparticles	Particle size: 7.47 ± 0.16 to 10.15 ± 0.21 nm; Zeta potential (ZP): -1.44 ± 5.36 to + 33 ± 6.45 mV	MCF-7	0.1 mg/mL	Induction of apoptosis and necrosis pathways	[165]
		Fe_3_O_4_ magnetic nanoparticles	Particle size: 9.74 ± 1.73 to 12.74 ± 3.58 nm; ZP: -74.6 to -77.9 mV	HeLa	100 μg/mL	DNA intercalation and inhibition of topoisomerase II Reactive oxygen species (ROS) via one-electron reduction Cell apoptosis	[166]
		Silica nanoparticles	Particle size: 140 ± 4 to 190 ± 5 nm; ZP: -1 ± 4 to -14 ± 4 mV	HepG2	1 to 100 μg/mL	Minimized the off-target cytotoxicity while killing the HepG2 cells	[167]
		Graphene-based nanomaterials	Particle size: 1 – 500 nm	HEp-2	0.625 μg /mL	Induction of p53 and p21 signaling resulting in G0/G1 cell cycle arrest Autophagy process Production of ROS	[168]
		Graphene Quantum Dots	Particle size: < 10 nm	U87	250, 200, 100 and 50 μg/mL	Alteration of the membrane permeability, facilitating the uptake of Dox inside U87 cells	[169]
Bleomycin	*Streptomyces verticillus*	Gold nanoparticles	Particle size: 29.3 ± 5.7 nm; ZP: -0.8 ± 0.6 mV	MIA PaCa-2 PC-3	0.04 mmol/mL	Gold-thiol bond (GNP-BLM) complexes were internalized via an endocytosis process. MIA PaCa-2 cell line seems to have a better response to the treatment compared to PC-3	[170]
Salinomycin	*Streptomyces albus*	Grapheneoxide–silver nanocomposite	-	A2780	2 μg/mL	Mitochondrial dysfunction ROS production Mitochondrial-mediated apoptosis and modulation of the down-regulation of anti-apoptotic Bcl-2 and up-regulation	[171]
		Iron oxide nanoparticles	Particle size: 84.1 ± 14 nm; ZP: + 0.8 mV	U251	30 μg/mL	Induction of ROS generation and apoptosis in U251 cells and inhibition of their proliferation	[172]
		Mesoporous silicon nanoparticles	Particle size: 100 to 200 nm; ZP: -30 mV	MCF-7	5.8, 58.8, 117.6, 235.3 mM/mL	Inhibition of cancer cells growth	[173]
Exotoxin A	*Pseudomonas aeruginosa*	Silver nanoparticles	Particle size: 522.8 nm; ZP: -0.152 mV	MCF‐7	0, 3.9, 7.8, 15.625, 31.25, 62.5, 125, 250, and 500 μg/mL	Exhibited an inhibitory effect on the viability of breast cancer cells through apoptosis	[174]
Listeriolysin O	*Listeria monocytogenes*	Gold glyconanoparticles	-	B16.F10 A375 MB-49 T-24 TC-1 A-549 NSCLC O627 Hepa1-6	50 μg /mL	Increased percentages of CD4 + and CD8 + T cells, B cells, and functional antigen-presenting dendritic cells (DCs) in tumor- infiltrated lymphocytes, while reduced the levels of myeloid-derived suppressor cells (MDSC) and suppressor T cells (T_reg_)	[175]
Mitomicyn C	*Streptomyces caespitosus*	Gold nanoparticles	Particle size: 2, 5 and 14 nm; ZP: + 2.8 to + 16.3 mV	MDA.MB.231 HDF	5 and 25 mmol/mL	MDA.MB.231 cells were found to be more sensitive than HDF cells, at all gold nanoparticles conjugated with 7-mercaptoethylmitomicyn C (Au@MEMC) concentrations and Au NP sizes, with cell death reaching 70% of the total cell number when exposed to 2.5 μM of Au@MEMC with particle size of 14 nm	[176]
Carfilzomib	*Actinomycetes strain*	Cobalt oxide nanoparticle	Particle size: 78 to 615 nm ZP: + 0.368 to -11.2 mV	U87	75 μg/mL	Block of the flow of Cfz-activated autophagy, which led to more toxic/non-degradable protein accumulation and synergistic killing of cancer cells	[177]

*MCF-7* breast cancer, *HeLa* cervical cancer, *HepG2* liver hepatocellular carcinoma, *U87* human glioblastoma brain cancer, *MIA PaCa-2* human pancreatic cancer, *PC-3* prostate cancer, *A2780* ovarian cancer cell, *U251* glioblastoma multiforme brain cancer, *B16.F10* murine metastatic melanoma, *A375* human melanoma, *MB-49* murine bladder tumor cell, *T-24* human bladder tumor cell, *TC-1* murine lung tumor, *A-549 NSCLC* murine lung tumor, *O627* murine glioblastoma cell, *Hepa1-6* murine hepatocarcinoma cell, *MDA.MB.231* human breast cancer, *HDF* human dermal fibroblasts

Even though inorganic NPs have been extensively used for biomedical applications, the studies about their long-term safety are still scarce and controversial [178]. In this sense, organic NPs have achieved greater interest from academia and industrial organizations for the encapsulation of different compounds, which despite having excellent pharmacological properties, possess noticeable limitations. Therefore, the nanoencapsulation of these anticancer agents can confer exceptional biocompatibility that results in lower toxicity in addition to facilitate the specific delivery of the compounds to target cells [179]. The amount and variety of compounds extracted from bacteria and loaded in organic NPs are relatively similar compared to inorganic NPs, with great efforts by the scientific community to explore a wider range of anticancer drugs. In this context, Sirolimus, Salinomycin, Rapamycin, Prodigiosin, Mitomicyn C, Marizomib, Enediyne, Doxorubicin and Carfilzomib are examples of bacterial metabolites loaded/encapsulated in diverse types of organic NPs as highlighted in Table 4.

**Table 4:** Natural compounds of secondary metabolites from bacteria loaded organic nanoparticles, their characterizations, and effects on cancer cell lines

Biological compounds	Bacterial source	Type of Nanomaterial	Nanoparticle properties	Target cell lines	Doses of biological compound applied on target cells	Effect on target cells	Reference
Sirolimus	*Streptomyces hygroscopicus*	Polymeric micelles composed by Poly(ethylene glycol)-b-poly(lactic acid) (PLA)	Particle size: 45.5 ± 2.2 to 60.4 ± 1.9 nm	Caco-2	100 μmol/L	Alteration of membrane fluidity Inhibition of ATPase activity Block of drug binding site Decrease of P-gp expression, depletion of ATP Interaction with membrane and interference with the ATP binding sites	[180]
Salinomycin	*Streptomyces albus*	Poly(lactic-co-glycolic acid) (PLGA) nanoparticles	Particle size: 185.8 ± 60.7 to 187.4 ± 63.3 nm ZP: + 49.3 ± 6.4 to + 51 ± 7 mV	MG-63	2.73, 7.51 and 15.02 μg	Induction of caspase-3 expression while suppressing β-catenin (Wnt/β-catenin pathway) and c-myc gene expressions in osteosarcoma cancer cells	[181]
Rapamycin	*Streptomyces hygroscopicus*	Lipid nanocapsules	Particle size: 92.3 ± 2.6 to 112.6 ± 8.4 nm ZP: -5.5 ± 0.5 to -8.6 ± 0.6 mV	U87MG	0.04; 0.2; 1; 5; 10; 20; 100; 200 μmol/L	Potent inhibitor of endothelial cell proliferation in vitro*.* Reduction of Vascular endothelial growth factor (VEGF) production by tumor cells and inhibition of VEGF induced proliferation in endothelial cells	[182]
		PLGA nanoparticles	Particle size: 211 ± 4 to 565 ± 9 nm ZP: -11.22 ± 0.29 to -18.27 ± 0.52 mV	C6	0.01–100 μg/mL	Induction of cell apoptosis	[183]
Prodigiosin	*Serratia marcescens*	Electrospun nanofibers scaffold composed by PLGA/gelatin	-	MDA-MB-231 MCF-7	5 mg/L	Reduction of cell viability and apoptosis	[184]
		Halloysite-based nanoformulation	-	Caco-2 HSF HCT116	100 μg/mL	Suppression of Caco-2 and HCT116 cells proliferation, followed by alteration of cell morphology and F-actin structure disorganization	[185]
Mitomicyn C	*Streptomyces caespitosus*	Soybean phosphatidyhlcholine complex loaded phytosomes	Particle size: 201.9 ± 2.4 nm ZP: -27.5 ± 1.67 mV	HeLa A549	1 mg/mL	Induction of cell apoptosis/death via the cytotoxicity enhancement effects of the nanoscaled phytosomes and, most importantly, folate (FA) receptor-mediated endocytosis	[186]
Marizomib	*Salinospora tropica*	PLGA nanoparticles	Particle size: 152.36 ± 0.03 nm ZP: -15.5 ± 0.03 mV	Caco-2 HepG2	30 μg/mL	Decrease of hepatocellular carcinoma (HCC) treated cells in the mitochondrial membrane potential	[187]
Enediyne	*Streptomyces carzinostaticus*	Small-molecule micelles composed by maleimide-based enediyne/gemcitabine	Particle size: 80.83 to 105.5 nm ZP: -14.2 to -18.8 mV	A549	100, 50, 25, 12.5, 6.25, 1, 0.1 and 0.01 μmol/L	Remarkable cytotoxicity toward cancer cells, and potential passive targeting ability due to the permeability and retention (EPR) effect	[188]
Doxorubicin	*Streptomyces peucetius*	Dexosome	Particle size: 99.2 ± 2.7 to 148 ± 2.9 nm ZP: -10.7 ± 7.39 to -20.2 ± 4.48 mV	A549	0.6, 1.2, and 1.8 μg/mL	Cytotoxicity against A549 cells showed that Dox loaded dexosomes (dExOI, dExOII, and dExOIII) induced higher cytotoxic effects than Dox dosages administered individually. Control groups demonstrated that A549 cell line was highly metastatic	[189]
		Lignin-based hollow nanoparticle	Particle size: 396 ± 13 to 405 ± 9 nm	HeLa	1 mg/mL	Inhibition of HeLa cells proliferation	[190]
		Solid lipid nanoparticles	Particle size: 130 ± 17 to 400 ± 10 nm ZP: -2.2 ± 0.84 to + 12.12 ± 1.52 mV	A2780	10^–4^ to 10^–2^ mmol/L	High activity of apoptotic mediators, such as caspase-3 and, even more clearly, with annexin-V	[147]
Carfilzomib	*Actinomycetes strain*	Liposomal nanoparticles	Particle size: ~ 70 nm ZP: -10.17 ± 1.78 to -12.73 ± 0.87 mV	MM.1S NCI-H929	2.10^–6^ mmol/L	Increase of proteasome inhibition and higher cytotoxicity than free carfilzomib	[191]
		Lipid nanodisk	-	U87MG HUVECs	0.005 mmol/L	There were significantly less viable cells and more apoptosis cells treated with carfilzomib-loaded lipid nanodisk modified with multifunctional peptide ^D^A7R (^D^A7R-ND/CFZ) than unmodified disks. However, free carfilzomib exhibited the strongest cytotoxicity, which was testified by either the MTT or cell apoptosis assay. This might relate to the incomplete release of carfilzomib from the nanodisks	[192]
		Ternary polypeptide nanoparticles	Particle size: 46.1 ± 4 to 56.5 ± 9.9 nm ZP: + 0.47 ± 0.12 to + 1.16 ± 0.29 mV	DLD-1	10 mg/mL	Increased intracellular drug accumulation, prolonged proteasome inhibition, and enhanced cytotoxicity of carfilzomib (CFZ) in drug-resistant DLD-1 colorectal cancer cells	[193]

*Caco-2* colon cancer cells, *MG-63* human osteosarcoma, *U87MG* glioblastoma cell, *C6* glioma cells, *MDA-MB-23* triple-negative breast cancer cell, *MCF-7* breast cancer cell, *HSF* human skin fibroblasts, *HCT116* human colon cancer cell, *HeLa* cervical cancer, *A549* adenocarcinoma human alveolar basal epithelial cells, *HepG2* liver hepatocellular carcinoma, *HeLa Kyoto* human cervical cancer cell, *A549* lung cancer, *A2780* human ovarian carcinoma, *MM.1S* lymphoblast cell line, *NCI-H929* human B lymphocyte, *HUVECs* human umbilical vein endothelial cell, *DLD-1* human colorectal cancer cell

More recently, a new class of nanomaterials known as metal–organic frameworks (MOFs) has been developed to encapsulate/conjugate natural compounds. Although inorganic NPs are highly stable and multifunctional, their biodegradability and biocompatibility have been contested, in addition to rapid drug release, limiting, thus, their use in clinical applications. Contrarily, organic carriers are known for their high biocompatibility and biodegradability, but low stability and single functionality [194]. MOFs have attracted interest for drug delivery and theragnostic science due to their controllable pore size, easily tunable compositions, and high surface area to volume, which is advantageous for drug loading capacity and stimulus-sensitive responses [195]. In addition, a large number of organic–inorganic hybrid materials have been developed in the course of several innovative projects dealing with photoactive, photocatalytic devices [196] that have been crucial in the treatment of cancer via phototherapy, radiotherapy, microwave, radiation and ultrasound therapy [197]. However, despite the multiple advantages of using MOFs as drug delivery systems, there is a paucity of works regarding the encapsulation of bacterial secondary metabolites. According to our best knowledge, the first work reported in the scientific literature using MOFs for encapsulation of bacterial compounds as a cancer therapy pathway was published in 2013 with the encapsulation of Doxorubicin [198]. In this work, the authors developed a new type of coordination polymer sphere prepared by combining of 1,10-(1,4-butanediyl)bis(imidazole) (bbi) and ferrous ions as a targeted delivery system for the encapsulation of anticancer drugs Dox [Dox/Fe(bbi)@SiO_2_]. Particle sizes between 150–250 nm are produced with 98% drug loading efficiency and showed sustained release of the drug for several days due to its superior stability being selective for HeLa cells (derived from human cervical cancer) via receptor mediated mechanisms of cell surface. In addition, cell viability experiments indicated that Fe(bbi)@SiO_2_ without Dox did not induce toxic effects on HeLa cells demonstrating, thus, that the decrease of cell viability was dependent on the effective delivery of Dox from Fe(bbi)@SiO_2_.

Despite the efforts, there are few studies in the literature related to the association between bacterial compounds and MOFs. In this context, Rastegari et al. [199] loaded Prodigiosin (PG) into Fe_3_O_4_ magnetic NPs (MNPs) grafted with carboxymethyl chitosan (CS) and cyclodextrin (CD) in order to evaluate the efficacy in MCF-7 cancer cell lines (human breast cancer) and HepG2 (human liver cancer). It was possible to verify that due to the intrinsic magnetic properties of the MNPs, the PG was exclusively directed to the target cells, while the -CS-CD-MNPs showed the apparent targeting capacity leading to an overexpress glucose, as well as, to a deliver and release of PG efficiently in cancer cells target, increasing antitumor efficacy and reducing side effects.

Rapamycin (RAP) is a lipophilic macrolide originally isolated from *Streptomyces hygroscopicus* [200] and is a promising therapeutic agent with immunosuppressive and antitumor properties, through specific inhibition mechanism of mTOR protein kinase (mammalian target of rapamycin) and prominently known as a strong inducer of autophagy and apoptosis [201]. However, its clinical applications are partially hampered by unfavorable bioavailability and pharmacokinetic profile [159]. A recent study conducted by Peng et al. [202] showed that the combination of DNAzyme) with an intelligent nanosystem assembled by a pure RAP core and a MOF shell could passively accumulate in tumor tissue, impose potent gene silencing efficacy, and inhibited tumor growth after intravenous administration in breast tumor-bearing mouse models, offering opportunities for combined TNBC gene therapy.

### Fungal metabolites-loaded NM

Fungi are a group of eukaryotes, which comprise two subcategories: A. Unicellular (e.g., Yeast) and B. filamentous (*e.g.*, molds) [203]. Endophytic fungi have been under intensive investigation due to their unique features, e.g., source of broad range of bioactive compounds, ease of culturing and constant production of potential secondary metabolites [204]. Endophytic fungi are symbiotic microorganisms that colonize several tissues of their hosts. They are ubiquitous organisms and present in almost all species of plants in either terrestrial [205] or marine environments [206].

The ever-famous fungal compounds, penicillin and statins have saved millions of lives. However, there are yet several other bioactive compounds with important therapeutic potential that have been isolated from endophytic fungi. For instance, camptothecin (isolated from *Camptotheca acuminata*) and hypericin (isolated from *Hypericum*) have anticancer and antibiotic properties, respectively [207–213]. In addition, a variation of bioactive compounds has been purified from these fungi, but they still need to be examined for their anticancer properties [214]. These bioactive compounds may be favored over the conventional chemical drugs owing to their high biocompatibility, high bioavailability, broad biodiversity, and safe mode of action on different healthy tissues. Comparably, the conventional chemical drugs which always come with drastic side effects. On the other hand, current studies have proven the positive effect of these secondary metabolites within several cancer therapies [208]. The main advantages of involving fungi in cancer fights are for example, their good response to routine culture techniques which facilitate upscaling production. Hence, fungi can be cultured in bulky amounts in bioreactors and stored for relatively prolonged period guaranteeing the availability of the source organism and the constant production of the needed bioactive compounds [215].

In modern pharmaceutical technology, nano-size materials are employed to shield and deliver the anti-cancer drugs to their specific target and reduce the loss of the drug throughout the delivery route. Several studies have also shown an improved cellular uptake of the drug when in nanoparticles formulation [216].

Hence, in the following text, we present an overview report on the recent research advancement about some of well characterized fungi bioactive compounds and their therapeutic importance, as well as the modern technologies to deliver such therapeutics in different nano-formulations. Moreover, we also present a brief look at the present challenges and future prospects.

### Bioactive fungal metabolites

#### Gliotoxin (GT) study

GT is an extensively studied bioactive compound derived from molds (mold is one of the structures certain fungi can form) of several fungal families, *i.e.,* Gliocladium, Trichoderma, Penicillium and Aspergillus. GT was discovered in 1932 as anti-microbial toxins produced by *Penicillium spp* [217, 218]. However, later investigation has also proved other therapeutic effects of GT, *e.g.,* immunosuppressive properties [219–221]. For instance, GT plays a critical immunosuppressive role in the lethal infection Invasive Aspergillosis (AI) [221]. Besides, GT has been also found to inhibit phagocytosis, inflammation, antigen presenting cells, T and B cells activation [222–224]. Most importantly, GT is also a vigorous anti-proliferative, pro-cell death factor, anti-angiogenic and anti-fibrotic agent [225]. Therefore, GT is considered a potential anti-cancer agent which is able to initiate cell death in target cell, either through apoptosis or necrosis. GT possesses a disulfide bridge in its structure that interacts with thiol groups on proteins involved in the production of reactive oxygen species ROS, leading to mitochondrial membrane disruption, DNA damage and then eventually cell death [226, 227]. In mice with prostate cancer, GT has shown very promising results to inhibit angiogenesis and suppress tumor gross, without causing weight loss in the treated mice [228]. Nonetheless, like other immunomodulatory agents, GT can be toxic for healthy tissues, so its use for human treatment has not been optimized yet. Thus, precise delivery technologies are needed. Such technologies would require targeted delivery of GT into malfunctioning cells, meanwhile avoiding exposing the surrounding healthy tissue to this toxic effect, and thus safely treating the disease without much compromise.

Comas et al. [218]*,* have developed magnetic NPs (MNPs) for intercellular delivery, wherein GT bound covalently to these MNPs. The outcome of this study showed an improved delivery of GT into cancer cells, meanwhile retaining its biological activity, and established a basis for incorporating GT in tumor therapeutics. MNPs have been widely used as drug delivery nano-vehicles due to their biocompatibility and low toxicity [229, 230]. They typically consist of an inorganic core and organic shell. The inner inorganic core is usually composed of magnetite and maghemite (Fe3O4, γ-Fe2O3). Meanwhile, the organic shell is the external surface of the MNPs, and it enables chemical functionality and plays a leading role in the solubility, stability, and interaction with other molecules. Hence, these MNPs can be covalently conjugated to various biological molecules and surface coatings, for instance, Polyethylene glycol (PEG) and carbohydrates. PEG enhances hydrophilicity and solubility in water. It also prevents MNPs aggregation and binding with non-specific molecules. While coating MNPs with certain carbohydrates (*e.g.,* Glucose) may improve cellular internalization through specific interaction with glucose transporters for example, which is highly expressed on cancer cells. Thus, When GT is bound to biocompatible MNPs functionalized with glucose moieties, the results showed that cellular internalization was improved meanwhile maintaining the biological activity of GT. However, GT have shown a potent effect on sarcoma cancer cells, meanwhile the effect on other cancer, i.e., breast cancer MCF-7 or cervix carcinoma HELA was humbler [218].

#### Manoscin (MNS)

Another example of potent fungal bioactive isolate is Manoscin (MNS), an extract of red mold rice *Monascus pilosus. Historically,* this species of fungi has been utilized for food making and meat preserving. Red-mod rice fermented with *Monascus* spp. Has potent therapeutic effects, *e.g.,* decreasing blood pressure [231] and plasma cholesterol level [232], besides exhibiting antimicrobial activity [233]. Recent study has also found that MNS was able to inhibit skin cancer development in mice [234]. Other studies have also investigated the anti-inflammatory, antioxidative and antineoplastic activities [234–236]. However, MNS is highly hydrophobicity which significantly reduces its bioavailability. Therefore, researchers have been developing delivering technologies to overcome this limitation and improve the bioavailability of this bioactive molecule. El-far et al. [237]*,* have developed casein micelles (CAS MCs) for delivering MNS. Naturally, Casein possesses amphiphilic properties because they are composed of hydrophilic and hydrophobic amino acids; hence it is easily self-assembled into nanosized micelles (~ 150 nm). Furthermore, CAS MCs can capture the hydrophobic cancer drugs within their hydrophobic core, facilitating their delivery, maintaining their bioactivity, and enhancing their bioavailability. For instance, the antitumor activity of flutamide, a hydrophobic cancer drug, in prostate cancer bearing animals was enhanced when incorporated in CAS MCs than the free drug [238]. Likewise, the multi drug resistance (MDR) in gastric cancer for paclitaxel (chemotherapeutic drug) and P-gp inhibitor (tariquidar) was significantly hindered when both drugs were coloaded in β-casein nano-vehicles [239]. The result presented in El-far et al., work has proven the efficacy of CAS MCs to deliver the fungal metabolite, MNS, maintaining its anticancer activity. When compared with free drug, this drug reservoir has shown an improved cytotoxicity, reduction in tumor growth and reduction in all the measure tumor growth biomarkers *(i.e.,* aromatase, VEGF, NF-kB and CD1) [240].

## Structural fungal material for nanoparticles synthesis

Besides fungal bioactive compounds, other structural fungal components are also being incorporated in fabricating nanocarrier, *e.g.,* fungal β glucan. For instance, Meng et al. [241] have developed dendritic nanoparticles derived from fungal β glucan to deliver the cancer drug, Doxorubicin. β-glucans are chiral polysaccharides that occur in the fungal cell wall, and they possess an intrinsic immunostimulatory activity especially on activating macrophages and dendritic cells. Therefore, β -glucans have been widely used as an anti-tumor adjuvant [242]. The unique chiral structure of glucans enables supramolecular interaction with pharmaceutical molecules based on chiral interaction rather than the frequently adopted electrostatic and hydrophobic interactions within the drug delivery systems. Electrostatic and hydrophobic interactions may influence the integrity of tissue proteins and cell membranes [243]. Moreover, β-glucans can self-assemble into nanoparticles making them a potential carrier for many therapeutics. All these features attracted researchers to further investigate β-glucans for drug delivery and anticancer therapeutic applications. Huang et al. [242] β-glucans with different chirality were investigated for DOX drug loading and encapsulation capacity, cellular uptake and immunoactivities activity. Their results have demonstrated that β-glucans can maintain strong immune activation as long as their chirality is maintained. In addition, a subtle variation in their chiral fingerprints may exhibit a considerable influence on the cytokines genes expression. Likewise, chirality played a key role in DOX drug encapsulation efficiency and drug release manner. Nevertheless, free DOX has exhibited higher cell toxicity when compared to the encapsulated DOX. According to Huang et al*.* [242]*,* this might happen due to the low molecular weight of free DOX which facilitates rapid cell internalization via diffusion, meanwhile, encapsulated DOX is released only under acidic conditions. Certainly, there are more investigations needed to further understand the potential of these structural carbohydrates in developing drug delivery systems for anticancer therapies.

Other fungal structural components have also been explored for the fabricating nano-carriers for drug delivery to target tumors, *e.g.,* extracellular vesicles secreted by fungi [244]. For instance, the EVs secreted by *Athrobotrys oligospora* were found to provoke the secretion of several proinflammatory cytokines and chemokines, referring to their potential as immunomodulating agents and adjuvants for tumor immune therapies. In association with DOX, these nanocomplex showed higher cytotoxicity than free DOX, when tested in several cancer cell lines [245]. However, in this study, DOX was associated with the nanoparticles outer surface only via simple electrostatic interaction. Thus, healthy cells can still be exposed to the anticancer drug, in addition to the plasma protein interaction with the nanoparticles may cause the drug to dissociate from the NPs causing burst release. Therefore, an alternative setting needs to be established for more efficient in vivo applications.

Despite the several advantages and therapeutic potential fungal-derived compounds show, there are challenges remaining to bring those compounds into clinical translation. More investigating research is needed to further understand the applicability for large scale production. Although several fungal metabolites have passed to the clinical trials [214], they still face difficulties to transform these good preclinical results into anticipated human responses because of their poor pharmacokinetic properties and solubilities [246, 247]. Researchers are constantly trying to reformulate these fungal metabolites and modify their structure to make them more suitable for biomedical applications. However, these formulas need to be examined in animal models and corresponding clinical trials to prove their competency. Eventually, pharma companies would have an essential role to further proceed forward with these formulations to the help patients.

### Plant compounds-loaded NM

For several years, the main source of medical treatments in developing countries have been plants, used as herbal medicines [248]. Several vegetables, fruits, herbs, and plant extracts have been used for cancer treatment, by inducing cellular apoptosis and inhibiting tumor proliferation [248, 249], since they present bioactive molecules with therapeutical efficacy [249]. For example, paclitaxel and its analogue docetaxel are chemotherapeutic agents used in clinics to treat several types of cancer, due to properties as microtubule disruptors, are plant taxanes [248, 249]. However, several of these phytochemicals, such as curcumin, quercetin, and resveratrol, present several disadvantages, such as low aqueous solubility, poor stability, fast metabolization, poor pharmacokinetics, low bioavailability, and poor target specificity towards cancer cells, with possible toxicity and multidrug resistance [250–252]. Besides, although plant compounds have been demonstrated to have antitumor properties in cell culture and animal studies, the results in human clinical trials are conflicting [250]. Therefore, several studies propose the loading of phytochemicals into biocompatible NPs, which can enhance their absorption and bioavailability, protect from liver metabolic degradation, increase the circulation time, and increase the drug uptake in cancer cells compared to healthy cells, reducing the side effects [250, 251]. Therefore, loading phytochemicals into NPs has been shown to increase therapeutic efficiency and decrease toxicity, which translates into better patient compliance [251, 253]. Several nanosystems have been formulated to encapsulate plant molecules or extracts for anticancer therapeutics, such as liposomes, nanoemulsions, SLNs, micelles, among others [250]. For example, ergosterol, a poor soluble plant sterol with anticancer properties, was encapsulated into poly(lactide-co-glycolide) (PLGA) NPs by emulsion/solvent evaporation technique, with increased cytotoxicity against glioma, hepatoma, and breast cancer. The oral administration of these polymeric NPs in mice presented a sustained drug release and a longer circulation time, being distributed specially in the stomach, brain, and liver, in smaller concentrations in the kidney, spleen and lung, and non-existent in the heart and lung. On the other hand, small amounts of sterols were detected in mice administrated with free ergosterol, which indicated that the PLGA NPs can improve the bioavailability, biodistribution and antitumor efficacy of poorly soluble plant compounds [254].

Herein, we access the recent research concerning the enhanced anticancer properties of plant compounds when encapsulated with NPs, being that the plant compounds are divided by chemical classes – alkaloids, polyphenols, benzophenones, quinones, terpenes. Also, we describe the properties of NPs loaded with plant extracts and oils, as well as the NPs produced by biogenesis.

### Alkaloids

Alkaloids are a class of naturally organic compounds with nitrogen containing heterocycles [255], and have demonstrated anti-inflammatory, neuroprotective, antimicrobial, and anticancer properties [255, 256]. However, their poor water solubility and the lack of specificity towards cancer cells [255, 257], leads to inadequate tissue discrimination and several side effects connected to their toxicity [255]. The use of nanocomposites, such as nanoemulsions, polymeric NPs [257] and lipidic NPs has shown to surpass these limitations and increase the anticancer potential of alkaloids, improving their bioavailability and providing passive and active targeting strategies. Therefore, in vitro and in vivo studies shown that loading several alkaloids into NPs increases their cytotoxicity for cancer cell lines and reduces the tumor growth and systemic toxicity, respectively (Table 5) [255, 257].

**Table 5:** Characteristics and anticancer properties of NPs loaded with of alkaloids

NPs properties	Cancer cell type	Dosage	Biochemical mechanism	Effect on target cells	References
Berberine					
Chitosan NPs Size: 258.2 ± 9.1 nm ZP: 34.6 ± 0.2 mV	Nasopharyngeal carcinoma (CNE-1)	20 mg/kg b.w	-	↑ Apoptosis ↓ Tumor Growth	[258]
Spherical chitosan NPs Size: 45 ± 5.6 nm ZP: + 39.82 mV	Lung cancer (urethane-induced)	75 mg/kg b.w. oral for 10 consecutive weeks	Modulation of gene expression: • BAX and VEGF receptor 2 • Lung caspase-9 and HIF 1	↓ Cancer Growth and Angiogenesis	[259]
Liquid crystalline NPs Size: 181.3 nm ZP: less than 5 mV	Lung cancer (A549)	IC_50_ = 10.1 µM	↓ SNAIL, P27, PDGF-AA, Axl, BCLx, Cathepsin S, Galectin-3, Survivin, CEACAM5, Pro-granulin, and ERBB3	Anti-migratory, colony formation and proliferation properties	[260]
NPs Size: 223 ± 1.8 ZP: -15.7 ± 0.1		-	↓ KRT18 mRNA ↑ PTEN and P53 mRNA ↓ AXL, CA9, ENO2, HER1, HER”, HER3, PRGN, and PDGF-AA proteins ↓ DKK1, CTSB, CTSD, BCLX, CSF1, and CAPG proteins		[261]
Au-collagen nanocomposites Size: 227 nm ZP: + 3.76 mV	Breast cancer (Her-2)	5–20 mg i.v	↑ Bax and p21 ↓ Bcl-2 and cyclin D1 ↓ MMP-9	Apoptosis, antimigratory properties In vivo tumor suppression	[262]
AgNPs Size: 113 ± 4 nm ZP: + 33.28 mV	Breast cancer (MDA-MB-231)	In vitro: IC_50_ = 1 μg/mL In vivo: 5 mg/Kg b.w. i.v	↑ ROS, cytochrome c, caspase-9, caspase- and Bax, mitochondria dysfunction ↓ Bcl-2 ↓ PI3K, AKT, Ras, Raf, ERK, VEGF, and HIF-1α	Apoptosis; nuclei dysfunction ↓ Tumor growth	[263]
Au nanorod within a silica stick Size: 200–250/100–120 nm ZP: + 4.7 mV	Liver cancer (SMMC-7721 and HL-7702)	25 mg/kg b.w. every 3 days + 1 Gy/min of X-ray radiation for 0, 5, 10 min	↑ Drug accumulation	Synergic with radiotherapy and photothermal therapy ↓ Tumor growth	[264]
HepG2 cell membrane coated MSNs Size: 57 nm ZP: -21 mV	Liver cancer (HepG2)	5 mg/kg b.w. i.v. every day (22 days)	↑ Immune escape capacity ↑ Targeting ↓ Blood clearance	↑ Accumulation of drug in tumor tissue ↓ Tumor growth	[265]
PLGA-HA copolymeric NPs Size: 101 ± 6.32 to 185 ± 15.62 nm	Ehrlich Ascites Carcinoma	0.45, 0.9, 2.25, 4.5, and 9.03 mg/Kg b.w i.v	↑ ROS levels, Mitochondria dysfunction	Apoptosis, cell cycle arrest at sub-G1 ↑Life span ↓Tumor-burden	[266]
Polylactic acid polymeric NPs Size: 265 ± 43 nm	Colon cancer (HCT116)	IC_50_ = 56.82 µM	↑ Drug accumulation	↓ Cell Viability	[267]
Camptothecin					
Antibody-targeting PLGA NPs Size: 127.2–170.8 nm	Colon cancer (HCT116)	IC_50_ = 0.37 ng/mL	↑ Cytotoxicity when compared with free drug	↓ Cell Viability	[268]
PLGA NPs Size: 206 ± 32 nm ZP: -21.1	Glioma (GL261-luc2)	20 mg/Kg b.w. i.v	-	↑ Camptothecin tumor levels ↓ Tumor growth ↑ Time of survival	[269]
SLNs Size: 130–160 nm ZP: -24 to -16.2 mV	Human glioma (A172, U251, U373, and U87)	IC_50_ = 0.2 to 8.61 µM	-	↑ Cancer cell toxicity ↑ Brain drug concentration and timespan	[270]
MSNs Size: 245 nm	Breast Cancer (MDA-MB-231)	50 mg/kg b.w. i.p. once weekly for 4 weeks	-	↓ Tumor Growth and Metastasis	[271]
Capsaicin					
Lipid NPs Size: 108.5 nm	Ovarian cancer (SKOV-3)	10 mg/kg b.w. i.v	-	↑ Cytotoxicity and in vivo pharmacokinetics	[272]
Inorganic NPs Size: 105 nm	Hepatocellular carcinoma (Hepg-2)	CAP dose of 4 mg/kg b.w. i.v every 3 days	Activation of TRPV1 channels ↑ Calcium ions and ROS Mitochondria dysfunction	↓ Tumor growth	[273]
Nanoliposomes Size: 60.65 ± 19.5 nm ZP: -13.87	Breast (MCF7, MDA-MB-231), Leukemia (K562), Pancreas (PANC1), and Melanoma (A375) cancer	IC_50_ values (µM): MCF7—13.66 MDA-MB-231—39.72 K562—17.88 PANC1—23.04 A375—13.32	-	Selective toxicity when compared with fibroblasts	[274]
Colchicine					
MSNs Size: 330 ± 22.2 nm ZP: ≥ + 40 mV in acidic medium	Colon (HCT116), Liver (HepG2) and Prostate (PC3) cancer	IC_50_ after 72 h of incubation (µg/mL) HCT116 – 17.09 ± 5.08 HepG2 – 76.59 ± 12.57 PC3 – 124.4 ± 12.1	Mitochondrial dysfunction and cytochrome c release ↑ Caspase-3 ↓ BRAF, CD44, MALAT 1, mir-205, PD-1, tubulin	Cell arrest at G2/M and apoptosis	[275]
Evodiamine					
Polymeric NPs Size: 157.36 ± 1.7 ZP:	Breast cancer (MCF-7)	-	↑ Cyclin B1 ↓ β-actin	Cell morphologic changes, G2/M cell cycle arrest and apoptosis	[276]
PLGA NPs Size: 255 nm ZP: − 19.02 mV	Colorectal (cancer (LoVo)	In vitro: IC_50_ = 9.732 µg/mL In vivo: 4 mg/kg b.w. i.v	↓ EGFR, VEGF, and MMP-2	↑ Cytotoxicity ↓ Invasion, adhesion, and migration ↓ Number of metastasis and tumor volume	[277]
Noscapine					
Gelatin NPs Size: 155 ± 15 nm ZP: -33.1 ± 0.8 mV	Breast Cancer (MCF-7)	IC_50_ = 21.2 µmol/L	Endocytosis mediated by estrogen receptors	↓ Cell Viability	[278]
Iron oxide nanoparticles Size: 35.62 ± 4.1 nm ZP: − 21.6 ± 3.3 mV	Prostate cancer (LNCaP, DU-145 and PC-3)	-	-	Potential use as magnetic resonance imaging and NIR imaging agent	[279]
Piperine					
Nanomicelle Size: 136.53 to 184.16 nm ZP: 12.6 to 16.4 mV	Glioma (Hs683)	IC_50_ = 0.67ug/ml	↓ CDK2a	G1 cell arrest	[280]
Hydroxyapatite NPs Size: ≥ 2500 nm ZP: negative	Colon cancer (HCT116)	-	-	HCT116 spheroid shrinkage and deformation	[281]

*b.w.* body weight, *EGFR* epidermal growth factor receptor, *HA* hyaluronic acid, *IC*_*50*_ half maximal inhibitory concentration, *i.p.* intraperitoneally, *i.v.* intravenous, *MMP* metalloproteinase, *MSNs* mesoporous silica NPs, *NIR* near-infrared, *NPs* nanoparticles, *PLGA* poly(lactic-co-glycolic acid), *ROS* reactive oxygen species, *SLNs* solid lipid NPs, *VEGF* vascular endothelial growth factor, *ZP* ζ-potential

### Berberine

Berberine (BBR) is a natural isoquinoline alkaloid which main sources are *Phellodendron amurense*, *Coptis chinesis*, and *Hydrastis canadensis*. This compound has antimicrobial, anti-inflammatory, antidiabetic, and chemotherapeutic properties [259, 261, 266]. BBR has presented anticancer properties due to its ability to induce apoptosis and cell cycle arrest and inhibit cell migration and invasion [266], while having less side effects when compared with other chemotherapeutic drugs [264]. However, its low absorption rate, stability and targeting delivery are some factors that disable BRR therapeutic use [259, 263].

**Fig. 11:**
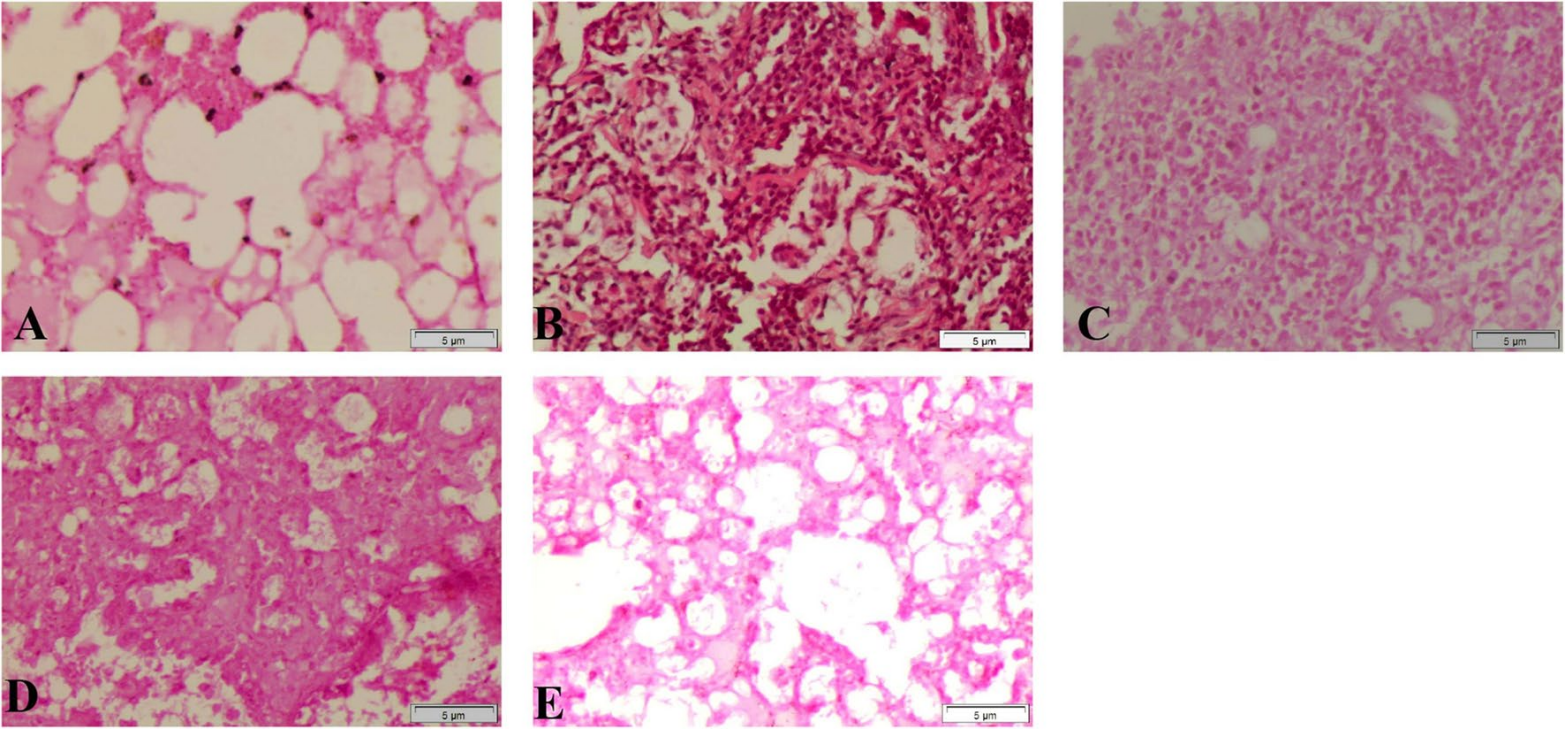
Histological photomicrograph of transverse lung sections: **A** control mice presented normal alveoli architecture with thin interalveolar septa constituted of simple squamous epithelial cells and normal interstitial tissues and alveolar sacs; **B** mice treated with urethane presented alveolar adenoma, cellular alterations (cells tend to be round, with stained cytoplasm, poor defined borders) and mildly differentiated squamous cell carcinoma; **C** mice treated with urethane also presented a defined alveolar adenoma demarcated from surrounding parenchyma as well as a mass of inflammatory cells infiltration, with damaging potential for the lung tissue; **D** mice treated with urethane and BBR displayed a partial alleviation of lung tissue degradation, with mild aggregations areas; **E** Mice treated with urethane and BBR-loaded chitosan NPs presented a significant reduction of the lung tissue degradation Reproduced with permission [259]. Copyright 2022, Elsevier B.V

For that reason, several studies have been encapsulating BBR in several types of NPs. For example, Wang et al. have demonstrated that BRR loaded into Chitosan NPs functionalized with FA modulated the migration, proliferation, and apoptosis of human nasopharyngeal carcinoma CNE-1 cells both in vitro and in vivo. The produced NPs presented pH-dependent release, inducing cancer cells apoptosis while restricting their mobility, resulting in a reduced tumor volume in mice [258]. BBR-loaded chitosan NPs also presented in vivo anticancer properties towards urethane-induced lung cancer. While control groups injected intraperitoneally with urethane presented increased levels of nitric oxide, NF-kB and HIF1-α and decrease glutathione (GSH), SOD, caspase 9 in the lung tissue, and high serum levels of Vascular Endothelial Growth Factor (VEGF) receptror-2, alanine aminotransferease, aspartate aminotransferase, urea, and creatinine, oral treatment with Chitosan NPs loaded with BBR (ChitosanNPs@BBR) modulated serum Bax and VEGF receptor-2 expressions, and lung caspase 9 and HIF 1 gene expressions, reducing cancer growth and promoting apoptosis, while inhibiting tumor angiogenesis (Fig. 11) [259]. BBR also demonstrated chemotherapeutic potential against non-small cell lung cancer when encapsulated into liquid crystalline NPs composed of monoolein and poloxamer 407. In vitro studies demonstrated and antiproliferative properties towards A549 cancer cell line (IC_50_ of 10.1 µM), as well as anti-migratory and colony formation properties, probably due to the inhibition of epithelial-mesenchymal transition (EMT)-related proteins, such as SNAIL, P27 and vimentin, as well as other proteins involved in the promotion of tumor proliferation and migration, such as PDGF-AA, Axl, BCLx, Cathepsin S, Galectin-3, Survivin, CEACAM5, Pro-granulin, and ERBB3 [260]. Another study concerning the BBR therapeutic potential against lung cancer produced liquid crystalline NPs produced by ultrasonication using poloxamer 407 and phytantriol as vehicle for BBR delivery. This nanocompound reduced A549 cells viability, due to the modulation of P53, PTEN, and KRT18 genes, and the downregulation of proteins associated with cell proliferation such as AXL, CA9, ENO2, HER1, HER3, HER3, PRGN, and PDGF-AA. Besides, A549 cell migration and colony formation were also inhibited due to the downregulation of DKK1, CTSB, CTSD, BCLX, CSF1, and CAPG proteins [261].

NPs loaded with BRR have also shown therapeutic potential against breast cancer [262, 263]. For example, a BBR loaded into nanocomposites of gold and collagen (Au-Col-BB) have demonstrated higher toxicity for Her-2 cancer cell line than to BAEC. This nanosystem presented were internalized into the cancer cells mainly via clathrin-mediated endocytosis and cell autophagy, inducing cancer cell apoptosis, by upregulating both Bax and p21 proteins, while suppressing anti-apoptosis proteins, such as Bcl-2 and cyclin D1, as well as reduced cancer cell migration by downregulating both metalloproteinase-9 (MMP-9) activity. Au-Col-BB also presented therapeutic properties in vivo, by reducing tumor size and increasing the time of survival [262]. Another study encapsulated BBR on citrate-capped silver NPs through electrostatic interactions, which were afterwards conjugated with conjugated with polyethylene glycol-functionalized folic acid (FA-PEG@BBR-AgNPs) to target the folate receptors, overexpressed in cancer cells. The resultant nanosystem presented higher toxicity against MDA-MB-231 breast cancer cells than HBL-100, a healthy breast cancer, inducing mitochondria dysfunction and increased reactive oxygen species (ROS) production, activating the pro-apoptotic factors cytochrome c, caspase-9, caspase- and Bax, while downregulating the antiapoptotic protein Bcl-2. On the other hand, FA-PEG@BBR-AgNPs also modulated the expression levels of proteins associated with tumor progression (PI3K, AKT, Ras, Raf, and ERK), angiogenesis (VEGF) and hypoxia (HIF-1α). Consequently, the nanosystem induced apoptosis, with nuclear alterations, such as nuclear shrinkage and bulging, nuclei fragmentation. Furthermore, in vivo studies showed that FA-PEG@BBRAgNPs inhibited tumor growth, without presenting any significant lesions in the lungs, liver, kidneys, heart, and brain [263].

Other studies have shown the potential of BBR-loaded NPs for liver cancer treatment. Li et al*.* [264] loaded BBR into Janus Au silica NPs conjugated with FA. This nanosystem presented dose-dependent cytotoxicity against SMMC-7721 cells and HL-7702 cells. Also, in vivo studies demonstrated the nanosystem synergic potential with X-ray radiation and photothermal therapy, reducing the tumor volume, without major side effects [264]. Yue et al*.* also loaded BRR into mesoporous silica NPs (MSNs) for the treatment of liver cancer. These NPs where then coated with human liver cancer HepG2 cell membranes to increase their targeting and reduce the blood clearance. The resulting nanosystem demonstrated antitumor properties both in vitro and in vivo, promoting the accumulation of BBR in tumor tissue, reducing the tumor volume and weight, without presenting relevant toxicity [265]. Additionally, BBR encapsulated into PLGA-HA copolymeric NPs presented anticancer properties in both HeLa and MCF-7 cancer cells, although the NPs uptake was higher in MCF-7 cells, due to the higher CD44 receptor density, which is targeted by HA. In vivo studies demonstrated that these NPs increase the ROS levels in EAC cells, with consequent mitochondria dysfunction, apoptosis, and cell cycle arrest at sub-G1, increasing the mice life span and decreasing the decreasing the tumor-burden in tumor-bearing mice [266]. Other polymeric NPs, composed of polylactic acid, were also studied for BBR delivery towards HCT116 colon cancer cells. The loaded NPs increased the cellular drug accumulation when compared with dree BBR, presented pH-dependent release and increased cytotoxicity towards cancer cells compared to NIN3T3, a non-neoplastic fibroblast call line [267].

### Camptothecin

Camptothecin (CPT), an alkaloid existent in the wood, bark, and fruit of the Asian tree *Camptotheca acuminate*, inhibits topoisomerase I, an enzyme involved in DNA replication that is overexpressed in several tumors, resulting in cell cycle arrest and apoptosis [268]. Therefore, CPT is an extremely cytotoxic chemotherapeutic compound, although it was poor water-solubility and is non-specific, failing in clinical trials due to its toxicity [271]. McCarron et al*.* loaded CPT into polymeric PLGA NPs, covalently attached to antibodies targeting the Fas receptor (CD95/Apo-1), overexpressed in colorectal cancer. It was found that, while CPT presented an IC_50_ of 21.8 ng/mL against HCT116 cancer cells after 72 h incubation, The polymeric CPT-loaded NPs exhibited an increased cytotoxic effect, with an IC_50_ of 0.37 ng/mL [268]. Studies have demonstrated that PLGA NPs loaded with CPT also presented antitumor potential against glioma, increasing the payload to 10 times higher in tumor compared to healthy brain tissue. The treatment of the NPs at 20 mg/Kg b.w. increase at CPT levels in the tumor site, decreasing the tumor growth and increased the time of survival compared to treatment with saline, free CPT, and NPs at 10 mg/Kg b.w, without adverse effects [269]. CPT have also presented increased antitumor potential when encapsulated within solid lipid NPs prepared using a lipid (cetyl palmitrate) and a surfactant (polysorbate 60 or 80). The encapsulation of CPT in NPs increased the drug uptake by an endocytic pathway and cell death in human glioma cell lines (A172, U251, U373, and U87), reducing the IC_50_ value compared to free CPT (A172: 93 × lower; U251: 99 × lower; U373; 896 × lower; U87: 129 × lower). Further in vivo experiments supported that the use of NPs as drug vehicle increased CPT concentration in serum and brain. Moreover, the CPT could be detected in brain until 24 h after i.v. administration, while free CPT could only be detected until 8 h after administration. On the other side, the accumulation of CPT increased in organs of the reticuloendothelial system, such as lungs, liver and spleen, responsible for NPs clearance [270]. More recently, Landgraf et al*.* prepared porous silicon NPs functionalized with cetuximab, an antibody targeting the epidermal growth factor receptor (EGFR), for CPT delivery. The produced NPs presented pH-responsive properties and have demonstrated anticancer properties in vivo against breast cancer induced by injection of MDA-MB-231 cells in the murine mammary fat pad. Overall, the tumor growth rate of mice treated with the loaded NPs was reduced compared with mice treated with unloaded groups, especially after the weeks 18 and 19, reducing the metastases in lung, liver and murine bone [271].

### Capsaicin

Capsaicin (CAP) is the main active compound of chili pepper (*Capsicum annuum*) and it is responsible for its hot taste [274]. Studies revealed the CAP plays a role at tumor development, as a carcinogen or as a cancer preventive substance [272, 282]. CAP has shown therapeutic properties against bladder, lung, breast, colorectal, stomach, prostate cancers, a nasopharyngeal carcinoma, among others [272, 274, 282], by inducing both mitochondrial intrinsic apoptosis and extrinsic death receptor pathways [272]. Besides, CAP has presented antitumor synergy with other chemotherapeutic agents (for example, 5-fluorouracil, cisplatin, pirarubicin, sorafenib) have been proved [282]. However, CAP therapeutic use is limited by its hydrophobicity, low affinity, and short half-life [273, 282]. Also, CAP is very irritant, causing skin and gastrointestinal pain and burn [274]. Therefore, its encapsulation into NPs and micelles have been studied to prolong the drug retention in the blood circulation, while increasing its targeting towards the tumor site and decrease the side effects [282]. Similarly, CAP loaded into lipid NPs produced by thin-film hydration technique, using 1,2-Distearoyl-sn-glycero-3-phosphoethanolamine-N- [methoxy (polyethylene glycol)-2000] folate presented more cytotoxicity towards SKOV-3 ovarian cancer cells than in healthy cells, probably due to the overexpression of folate receptors in cancer cells, increasing the receptor-mediated endocytosis of these lipid NPs. Moreover, the lipid NPs increased the CAP pharmacokinetics, probably due to the presence of PEG in the NPs structure [272]. More recently, Xu et al*.* [273] produced calcium carbonate NPs to load CAP, providing an additional efflux of extracellular calcium ions to cause cancer cell death. The resulting NPs presented pH-responsive properties, activating the TRPV1 channel on the Hepg-2 cells membrane, resulting in an excessive influx of calcium ions, with consequent mitochondria dysfunction and increase of intracellular ROS. In vivo studies have shown that the tumor growth of mice treated with loaded NPs was significantly decreased when compared with no treated mice, or mice treated with free drug, with elevation of the caspase-3 levels and the calcium weight per gram of tumor tissue, with no relevant tissue damage [273]. Finally, Al-Samydai et al*.* demonstrated the potential of CAP against several tumors, when loaded into nanoliposomes prepared using the thin-film method. The loaded NPs presented selective toxicity towards both MCF-7, MDA-MB-231, A375, K562 and PANC-1 cancer cell lines, when compared with normal cell fibroblasts [274].

### Other alkaloids

Other alkaloids have been encapsulated into NPs for antitumor therapy. Colchicine (COL), a natural alkaloid derived from the plant *Colchicum automnale*, is an antimitotic drug, since it affects the microtubule assembly during mitosis, having potential as a chemotherapeutic agent [275]. However, COL also presents severe side effects in patients. Therefore, its encapsulation into MSNs functionalized with phosphonate groups and coated with a FA chitosan-glycine complex increased the toxicity against HCT116 and, in less scale, against HepG2 and PC3 cancer cells, especially at 100 and 200 µg/mL, without presenting significant effects on normal BJ1 cells. The loaded MSNs induced HCT116 cells apoptosis by inducting mitochondrial dysfunction and cytochrome c release, with caspase-3 activation, while inhibiting the expression of the anti-apoptotic proteins BRAF and CD44 metastasis-associated lung adenocarcinoma transcript 1 (MALAT 1), microRNA (mir-205), programmed cell death protein 1 (PD-1) and Ang-2, a pro-angiogenesis cytokine that limits the immunologic attack against cancer cells. The NPs also inhibited tubulin activity, inducing cell arrest at G2/M and apoptosis [275].

**Fig. 12:**
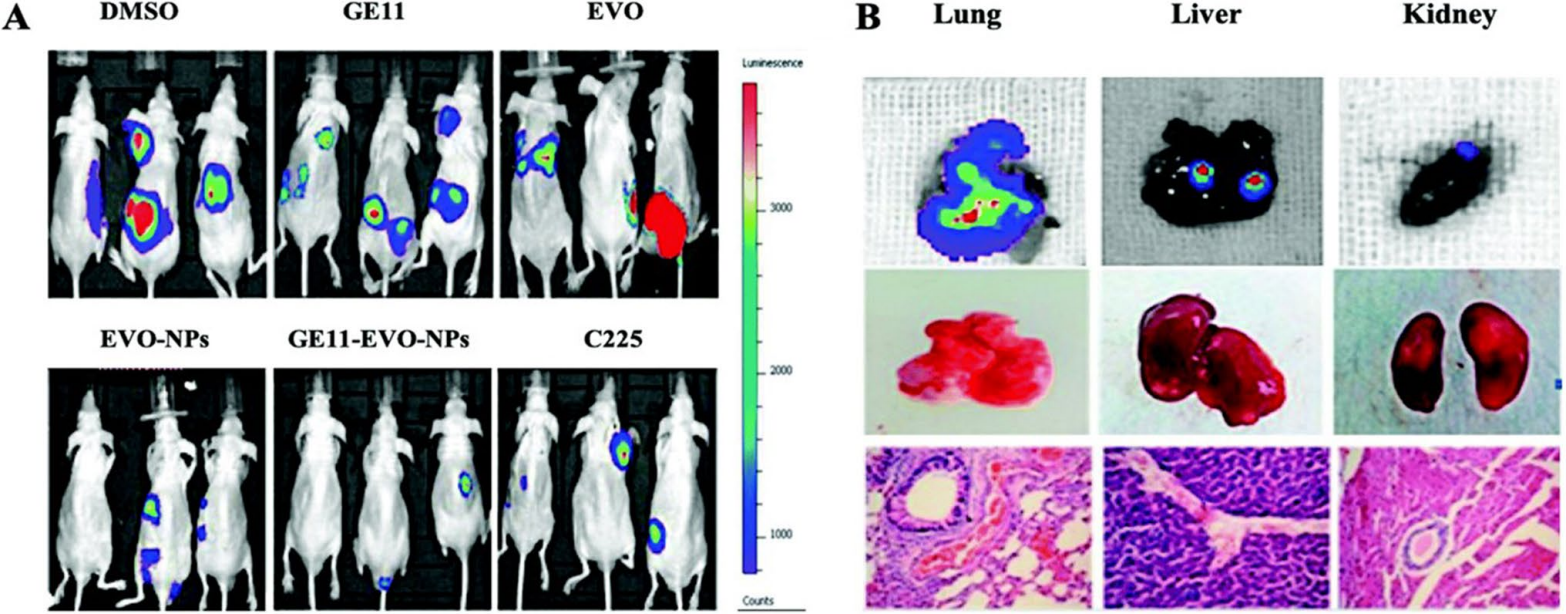
In vivo and ex vivo of mice after treatment with DMSO, GE11, Evo, Evo-NPs, GE11-Evo-NPs, and C225. **A** In vivo and **B** ex vivo bioluminescence images of lung, liver, and kidney metastases in the mice and follow H&E staining of metastasis tumor in the selected organs indicates the antimetastatic potential of GE11 NPs loaded with EVO. Reproduced with permission [277]. Copyright 2019, Royal Society of Chemistry. DMSO: Dimethyl sulfoxide; Evo: evodiamine; NPs: nanoparticles

Other plant-derived alkaloid that also showed increase antitumor properties when encapsulated in NPs is evodiamine (EVO), an indolequinazoline alkaloid known to alter the balance of Bcl-2/Bax gene expression in cancer cells, although with poor aqueous solubility [276]. To increase its solubility, Zou et al*.* loaded EVO into PLGA NPs by single emulsion (o/w) solvent evaporation method. The loaded PLGA-NPs increased the expression of cyclin B1 and decrease the expression of β-actin in MCF-7 breast cancer cells, resulting in cell morphologic changes (long spindle-shape cells turn round, with widened intracellular gaps), G2/M cell cycle arrest and enhanced apoptosis compared to free drug [276]. Additionally, Li et al*.* loaded EVO into poly(amino acid) NPs comprised of GE11, a peptide ligand targeting EGFR and study the in vitro and in vivo therapeutic potential of this nanosystem against colorectal cancer (Fig. 12). The loaded NPs presented increased cytotoxicity, reducing the invasion, adhesion, and migration of cancer cells i*n vitro*, by downregulating both EGFR, VEGF, and MMP-2 expressions, while reducing the tumor volume and the number of cancer metastasis in lung, liver, and kidney in nude mice [277].

Noscapine (Nos) is plant alkaloid derided from opium, used an oral antitussive with low toxicity in humans [283]. Also, it is also a tubulin-binding agent undergoing several clinical trials, being reported to be effective against lymphoma and multiple myeloma [278, 283]. However, Nos has a low aqueous solubility, poor absorption, and short biological half-life, with high first-pass metabolism that makes difficult an oral controlled-release formulation [278]. For this reason, Nos encapsulation into several types of NPs has shown to be an advantageous strategy for its drug delivery. Madam et al*.* encapsulated Nos into gelatin NPs functionalized with estrogen to target receptor-positive breast cancer MCF-7 cells, resulting in increased cytotoxicity when compared to free drug [278]. Also, Abdalla et al*.* loaded Nos into iron oxide NPs functionalized with a human-type 135 amino-acid amino-terminal fragment of urokinase plasminogen activator, that has a high-affinity towards the urokinase plasminogen activator receptor, overexpressed in prostate cancer, producing a nanosystem with pH-responsive behavior and that specifically targets and accumulates in prostate cancer cells, as well as with potential to be use as magnetic resonance imaging and near-infrared (NIR) imaging agent [279].

Piperine (PIP), another plant alkaloid with origin in the fruit of black pepper plants *Piper Nigrum L.* [280, 284], have also been encapsulated into NPs in way to reverse its poor solubility and permeability limitations and increase its antitumor and anti-metastatic properties [280], including chitosan-sodium tripolyphosphate, solid lipids, PLGA, chitosan NPs, and self-emulsifying drug delivery [281]. For example, Sedeky et al*.* loaded PIP into PF127 nanomicelles coated with positively charged trimethyl-chitosan, forming a core–shell structure. This nanostructure has presented higher cytotoxic properties against Hs683 cancer cells when compared with free drug, inducing G1 cell arrest, by inhibiting the cell cycle regulation protein CDK2a [280]. PIP have also presented increased antitumor properties when encapsulated in hydroxyapatite NPs functionalized with phosphonate groups to increase the interconnection between the articles, FA, as a targeting agent, and coated with gum Arabic. The loaded NPs presented a high cytotoxic effect against HCT116 colon cancer cells, although its anticancer properties were weaker against Caco2 colon cancer and MCF-7 breast cancer cells. The NPs also presented inhibitory properties against HCT116 spheroids, which could be observed by smaller cell size and the deformation of the spheroids [281].

### Terpenoids

Terpenoids are the greatest and most diverse group of natural phytocompounds, being. associated with the fragrance, taste, and pigmentation of plants. The number of carbons formed by isoprene units is used for their classification into monoterpenoids, sesquiterpenoids, diterpenoids and triterpenoids. Terpenoids have presented antitumor properties, by both exacerbate the ROS production, leading to mitochondrial depolarization and caspase activation, and by modulate the immune system as well. However, NPs have shown to enhance their uptake into cancer cells, improving their anticancer efficacy (Table 6) [284].

**Table 6:** Characteristics and anticancer properties of NPs loaded with of terpenoids

NPs properties	Cancer cell type	Dosage	Biochemical mechanism	Effect on target cells	Reference
Celastrol					
FA-AuNPs Size: 22.00 ± 4.48 nm	Breast Cancer (MCF-7)	Cell viability of 7.76 ± 1.56% at 20 µM under 24 h	Mitochondria dysfunction Changes in cell morphology Nuclear shrinkage	Early apoptosis ↑ Inhibition of cancer spheroid growth	[285]
MSNs Size: 150 nm	Breast cancer (BT-474), Neuroblastoma (SH-SY5Y) and neck squamous carcinoma (SCC7)	In vitro studies: IC_50_ values (µM): BT-474 – 1.1 SH-SY5Y – 31.1 In vivo studies (SCC7): 1 mg/kg b.w. i.v. four times every 3 days	Mitochondria dysfunction ↑ Cytochrome release ↑caspase 3/9 and PARP expression	Increased antitumor efficacy, decreasing the tumor size	[286]
Nanomicelles Size: 48 nm	Retinoblastoma (SO-Rb50)	27.2 mg/kg/2 days b.w. i.p for 16 days	↓ HIF-1α/VEGF pathway	↓ Migration, and invasion human umbilical vein endothelial cells ↓ Tumor growth and Angiogenesis	[287]
Ginsenoside					
Ginseng-derived extracellular vehicles Size: 141.5 nm	Melanoma (B16F10)	250 μg/100 mL i.p. every four days	Polarization of tumor supportive M2 into tumoricidal M1 macrophages ↑ IL -1/6/12, TNF-α, MCP-1, CCL5 ↑ ROS production	↓Tumor growth, angiogenesis, and cell invasion	[288]
Bovine Serum Albumin NPs Size: 201.4 nm ZP: − 22.5 mV	Breast cancer (MCF-7)	In vitro studies: IC_50_ values (µM): 24 h – 26.55 ± 0.84 48 h – 19.75 ± 2.58 In vivo studies: 0.5 mg/kg b.w. i.p	-	↓ Tumor volume	[289]
Graphene oxide nanoparticles	Osteosarcoma (MG63 and U2OS)	-	Co-treatment with near-infrared light: ↑ ROS production, ↑ Bax and caspase-3 ↓ Bcl-2	Cancer cells apoptosis and autophagy ↓ Tumor growth	[290]
Manganese dioxide nanoparticles Size: 141.5 nm	Osteosarcoma (K7M2)	10 mg/kg b.w. i.v	↑Oxidative stress ↑ Bax, BCL-2, and Caspase-3. CD4 + /CD8 + T cells activation ↑ IL-6, interferon-γ. and TNF-α FOXP3 + T cells (Tregs) suppression	↓ Tumor growth Immunomodulation of the tumor microenvironment Magnetic resonance imaging properties	[291]
Linalool					
SLNs Size: 90–130 nm ZP: − 4.0 mV	Lung cancer (A549) and hepatocellular carcinoma (Hepg2)	-	-	Antiproliferative properties	[292]
AuNPs Size: 13 nm	Ovarian cancer (SKOV-3)	-	↑ Oxidative stress and mitochondria dysfunction ↑ Cytochrome, caspase-3/7/8/9, p53, tBid, Bax, Bad, Bim, SMAC, HtrA-2, Fas, Hsp60, HTRA, IGF-1/2, IGFBP1/4, P21, P27, survivin, TNFR-I/II, TRAIL-1/2/3/4 ↓ Bcl-2, Bcl-w, cIAP-2, XIAP, LIVIN, Hsp27, IGFBP5/6, IGFBP6, IGF-IR, TNF-α/β and XIAP	Cancer cell apoptosis, with cell and nuclear membrane disruption, DNA fragmentation and migration	[293]
Triptolide					
Exosomes Size: 159.9 ± 2.7 nm ZP:	Ovarian cancer (SKOV-3)	In vitro studies: IC_50_ values (ng/ml): 24 h – 270.3 48 h – 18.93 In vivo studies: 0.2 mg/kg b.w. i.p	-	Cell cycle arrest at S phase (24 h) and at G0/G1 ↑ Tumor suppression ↓ Side effects	[294]
Polymeric nanoparticles Size: 73.4 ± 5.2 nm ZP: 57.3 ± 6.69 mV (pH = 9.6); 28.7 ± 5.1 mV; (pH = 7.0); 21.1 ± 4.73 mV (pH = 5.6)	Colorectal adenocarcinoma (HT29)	In vitro studies: IC_50_ values (nM): 24 h – 128 ± 79.5 48 h – 38.9 ± 10.3 In vivo studies: 0.9 mg/kg b.w. i.v. 3x/week	-	Cell cycle arrest at G0/ G1 phases ↑ Apoptosis ↑Mice Survival Rate ↓ Tumor growth	[295]
Polymeric nanoparticles Size: 191.31 ± 7.08 nm ZP: -6.94 ± 0.58	Breast Cancer (4T1, MDA-MB-231, MCF-7)	In vitro studies: IC_50_ values (nM): MDA-MB-231 pH 7.4 – pH 7.4 with 10 mM GSH – pH 5.8 – MCF-7 pH 7.4 – pH 7.4 with 10 mM GSH – pH 5.8 – In vivo studies (4T1): 0.9 mg/kg b.w. i.v. 3x/week	↓Bcl-2, MMP-9 and CD31 ↑ p53, caspase-3 and E-cadherin	G0/G1 cycle arrest ↓ Cancer cells motility, migration, and invasion ↑ Mice survival ↓ Tumor growth and metastasis	[296]
Chitosan NPs	Hepatocellular carcinoma (SMMC-7721)	In vitro studies: IC_50_ values (µg/mL) Triptolide – 1.61 ± 0.69 Triptolide NPs – 2.08 ± 0.53 In vivo studies Triptolide – 1 and 2.5 mg/kg Triptolide NPs – 6.76 to 16.90 mg/kg b.w. intragastrical administration	↓ TNF/NF-kB/BCL2 signaling (TNF, NFKB1, NFKB1A, RELA, BCL2 and XIAP proteins)	Apoptosis ↓ Side effects compared with free triptolide	[297]

*b.w.* body weight, *FA* folic acid, *GSH* glutathione, *IC*_*50*_ half maximal inhibitory concentration, *i.p.* intraperitoneally, *IL* Interleukin, *i.v.* intravenous, *MMP* matrix metalloproteinases, *MSNs* mesoporous silica NPs, *NPs* nanoparticles, *ROS* reactive oxygen species, *SLNs* solid lipid NPs, *TNF* tumor necrosis factor, *VEGF* vascular endothelial growth factor, *ZP* ζ-potential

### Celastrol

Celastrol is a pentacyclic terpenoid extracted from *Tripterygium wilfordii* roots [285, 286] and has been used in Chinese medicine for cancer treatment, by inhibiting proteasome, topoisomerase activity, the expression of VEGF and inducing apoptosis [285]. However, it also presents poor water solubility, low oral bioavailability, short plasma half-life, and poor tumor targetability, being that the use of nano delivery systems has showed promise to increase celastrol delivery and therapeutic efficiency [285, 286].

**Fig. 13:**
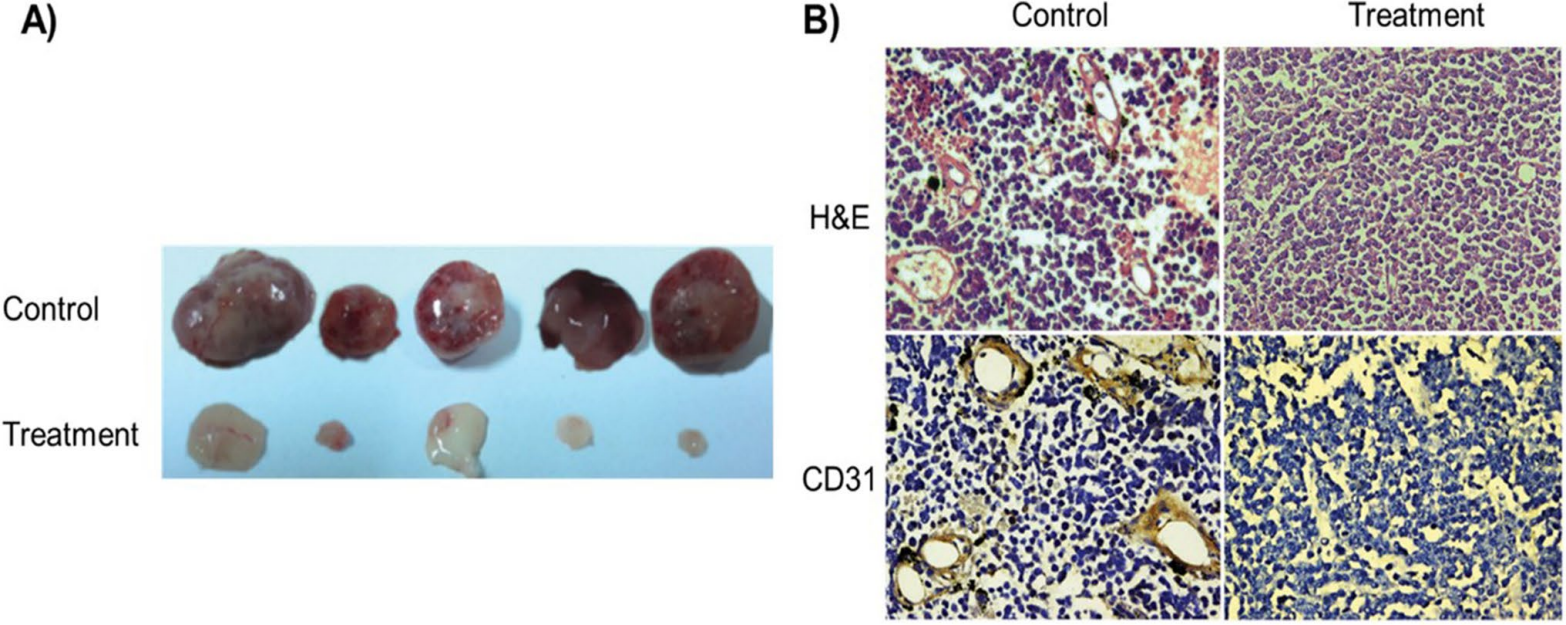
Nanomicelles loaded with celastrol have presented antitumor properties, reducing the tumor growth (**A**) and the neovascularization (**B**) in SO-Rb50 tumor-bearing mice. Adapted with permission [287]. Copyright 2019, Taylor & Francis Online ~ . H&E: Hematoxylin and Eosin

Law et al*.* produced AuNPs conjugated with celastrol, and a polymer named PVP-co-2-dimethylaminoethyl methacrylate, to confer pH-responsive properties, and functionalized with FA, to target MCF-7 cancer cells. The conjugated and functionalized AuNPs presented increased anticancer properties compared to free drug and the conjugated AuNPs, causing mitochondria dysfunction, changes in cell morphology and nuclear shrinkage, and increased early apoptotic cells. Furthermore, treatment of 3D tumor spheroids with the conjugated and functionalized AuNPs resulted in higher cell growth inhibition compared with free drug and bare or functionalized AuNPs did not shown any growth inhibition (Fig. 13) [285].

Choi et al*.* loaded celastrol into MSNs capped with PEGylated polyaminoacid to enhance the systemic delivery by inducing a controlled drug release and evading macrophages clearance. The nanosystem presented higher anticancer properties than free celastrol, by increasing the expression of HIF-1α and causing mitochondria dysfunction, leading to cytochrome c release and caspase-9 activation. In vivo studies concerning SCC7 tumor-bearing xenograft model also showed that the nanosystem increased both caspase-3 and PARP expressions, significantly decreasing the tumor volume, without being toxic to healthy cells [286].

**Fig. 14:**
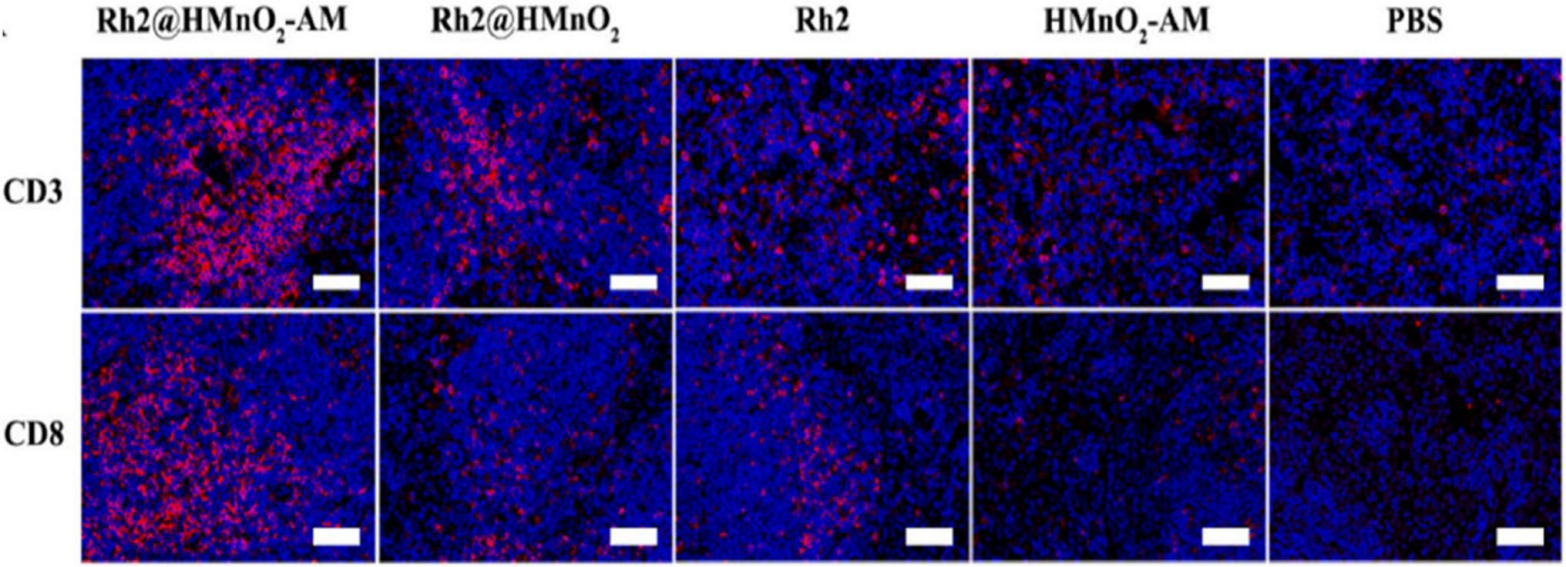
Tumor tissue immunofluorescence staining of CD3 + and CD8 + T cells. It is possible to observe that Rh2@HMnO2-AM group have the largest distribution of CD3 + and CD8 + T compared to the other treatments, followed by the Rh2@HMnO2, Rh2, HMnO2-AM, and PBS groups. Adapted with permission [291]. Copyright 2022, Elsevier.; AM: alendronate/K7M2 cancer cell membranes; HMnO_2_: hollow manganese dioxide; NPs: nanoparticles

Additionally, celastrol loaded into nanomicelles have also shown to inhibit angiogenesis induced by growth of retinoblastoma, both in vitro and in vivo, reducing hypoxia-induced proliferation, migration, and invasion of EA.hy 926 human umbilical vascular endothelial cells, by inhibiting the HIF-1α/VEGF pathway, while inhibiting tumor growth and vascularization in SO-Rb50 tumor-bearing xenograft model (Fig. 14) [287].

### Ginsenoside

Ginsenosides are triterpene saponins and are the active ingredient of ginseng [289, 291]. Ginsenosides can be divided into several types (Rh2, Rh3, Rg1, Rg2, Rg3), each one with different properties. For example, while Rg1 is known for its memory improvement and antiaging properties, Rg2 has beneficial cardiovascular and immunomodulatory properties as well as inhibits cancer cells growth [291]. Also, Rg3 and R5 have been shown to prevent angiogenesis and induce cancer cell apoptosis [289, 290].

However, Cao et al*.* used fresh ginseng roots to produce ginseng-derived extracellular vehicles, nanomembranes composed by lipids and proteins that showed to induce the polarization of tumor-supportive M2 into tumoricidal M1 macrophages via Toll-like receptor (TLR)-4 and myeloid differentiation antigen 88 (MyD88)-mediated signaling, as well as increased the production of M1 markers, such as interleukin (IL) -1/6/12 and TNF (Tumor Necrosis Factor) -α, monocyte chemoattractant protein-1 and chemokine ligand 5. Furthermore, in vivo studies in mice bearing B16F10 melanoma demonstrated with the extracellular vehicles not only switch from an immune-supportive M2-like macrophages (CD11b + F4/ 80 + CD206 +) to tumoricidal M1-like macrophages (CD11b + F4/80 + CD86 +) in the TME, but also induced ROS stress, inhibiting tumor growth, angiogenesis, and cell invasion. Therefore, the ginseng-derived extracellular vehicles can act as an cancer immunotherapeutic agent [288].

Dong et al*.* encapsulated Rg5 into bovine serum albumin NPs functionalized with FA by desolvation method, to produce a nanocompound with pH-responsive behavior that presented higher toxicity towards MCF-7 breast cancer cells than L929 fibroblasts. Also, in vivo studies demonstrated that the nanocompound reduced the tumor volume in mice compared to free Rg5. In fact, in vivo real-time bioimaging allowed to observe that the functionalization with FA direct the noncompounds towards the tumor site (Fig. 14) [289].

Lu et al*.* studied the potential of ginsenoside Rg3 for osteosarcoma, when loaded into PEG-graphene oxide NPs, conjugated with the photosensitizer indocyanine green and folic acid. The nanosystem presented photodynamic properties when in co-treatment with near-infrared light, by increasing ROS production, activating both Bax and caspase-3, while downregulating Bcl-2, which resulted in enhanced apoptosis and autophagy of MG63 and U2OS cancer cells by autophagy. In vivo studies confirmed the existence of synergy between the laser and the treatment with the Rg3 loaded nanosystem, reducing the osteosarcoma tumor growth and confirming the nanosystem potential for photodynamic treatment [290].

More recently, Fu et al*.* also ginsenoside Rh2 also presented anticancer properties against osteosarcoma when loaded into manganese dioxide (HMnO_2_) NPs modified with alendronate and coated with K7M2 cancer cell membranes (AM). The nanosystem not only presented cytotoxicity towards cancer cells, but also triggered immunogenic cell death by inducing the maturation of dendritic cells. The immuno-chemotherapeutic combined therapy increases the oxidative stress and induces the Bax, BCL-2, and Caspase-3 overexpression. In vivo studies also supported the magnetic resonance imaging contrast properties of the nanosystem produced, as well as its potential to activate CD4 + /CD8 + T cells (Fig. 14), enhance the secretion of interleukin-6, interferon-γ. and TNF-α and inhibit FOXP3 + T cells (Tregs), without demonstrating significant side damage [291].

### Linalool

Linalool is a monoterpene found in essential oil that, although has antiproliferative properties, presents poor aqueous solubility and high toxicity [292, 293]. Therefore, Rodenak-Kladniew et al*.* purposed its encapsulation into SLNs of myristyl myristate, cetyl esters and cetyl palmitate, prepared by sonication using Pluronic® F68 as surfactant. The loaded SLNs presented in vitro antiproliferative properties against hepatocarcinoma (HepG2) and lung adenocarcinoma (A549) cell lines up to 1.0 mM, however the cetyl palmitate SLNs did not present anticancer properties at the concentration used [292]. Further studies loaded linalool into AuNPs capped with GSH and conjugated with a CALNN peptide, which enhanced its cellular delivery and increased their aqueous solubility, The loaded AuNPs presented anticancer properties against SKOV-3 ovarian cancer cells, by increasing the intracellular superoxide production, causing mitochondria dysfunction, with release of cytochrome c into the cytoplasm, and upregulation of caspase-3/9. Furthermore, an increase in the expression of p53, and caspase-8 was observed, which also induced caspase-3/7, having the ability to crosslink with the mitochondrial pathway by cleaving Bid to tBid. The overexpression of other pro-apoptotic proteins (Bax, Bad, Bim, SMAC, HtrA-2, Fas, Hsp60, HTRA, IGF-1/2, IGFBP1/4, P21, P27, survivin, TNFR-I/II, TRAIL-1/2/3/4) and the downregulation of antiapoptotic proteins (Bcl-2, Bcl-w, cIAP-2, XIAP, LIVIN, Hsp27, IGFBP5/6, IGFBP6, IGF-IR, TNF-α/β and XIAP) was also observed, indicating that cancer cell death by both the intrinsic and extrinsic apoptosis pathways. SKOV-3 cancer cells presented cell and nuclear membrane disruption, with DNA fragmentation and migration, indicating cell death [293].

### Triptolide

Triptolide (TP) is an active compound present in the Chinese herb *Tripterygium wilfordii* Hook F [294, 297], presenting both immunosuppressive and anti-inflammatory properties, but also an antitumor activity since it acts as a topoisomerase I [295]. However, its therapeutic use is limited by its toxicity, causing hepatic and kidney damage, as well by its poor water solubility [295, 297]. Therefore, to increase its encapsulation efficiency into cancer cells, Liu et al*.* encapsulated TP into SKOV3-exosomes by sonication and ultrafiltration centrifugation. The loaded exosome demonstrated high encapsulation efficiency towards SKOV3 ovarian cancer cells, inducting cell cycle arrest at S phase after 24 h incubation, and at G0/G1 phases after 48 h incubation, and consequent apoptosis. In vivo studies also demonstrated that while the loaded exosomes presented increased tumor suppression, the liver and kidney side effects decreased when compared with free TP [294].

Also, the encapsulation of TP into PGA-L-Phe polymeric NPs increased their anticancer properties, inducing G0/G1 cycle arrest in HT29 colorectal cancer cells and, therefore, apoptosis. Besides, encapsulated TP significantly improved the survival rate and the reduced the tumor growth in HT29 bearing mice, while presenting lower nephrotoxicity and liver toxicity when compared with free TP [295]. Other polymeric NPs composed of hyaluronic acid-vitamin E succinate and poly (ß-amino esters) functionalized with HA demonstrated to increase TP anticancer properties. Poly (ß-amino ester) is a positive charged biocompatible and biodegradable polymer, and vitamin E succinate conjugates with HA via an esterification reaction to create an amphiphilic copolymer. The encapsulated NPs entered MDA-MB-231 breast cancer cells via caveolae-mediated and cholesterol-dependent endocytosis, presenting better capability in suppressing their proliferation and inhibiting their motility, migration, and invasion than free TP. Also, in vivo studies concerning breast cancer 4T1 tumor-bearing nude mice showed that the TP encapsulation in the polymeric NPs reduced the tumor growth compared to free TP, with reduction of the expression of Bcl-2 activity and upregulation of p53 and caspase-3, suggesting apoptosis mediated by the mitochondrial apoptotic pathway. Besides, the encapsulated TP also inhibited angiogenesis and restrained the formation of lung metastasis nodules via MMP-9 and CD31 downregulation, and E-cadherin upregulation [296].

**Fig. 15:**
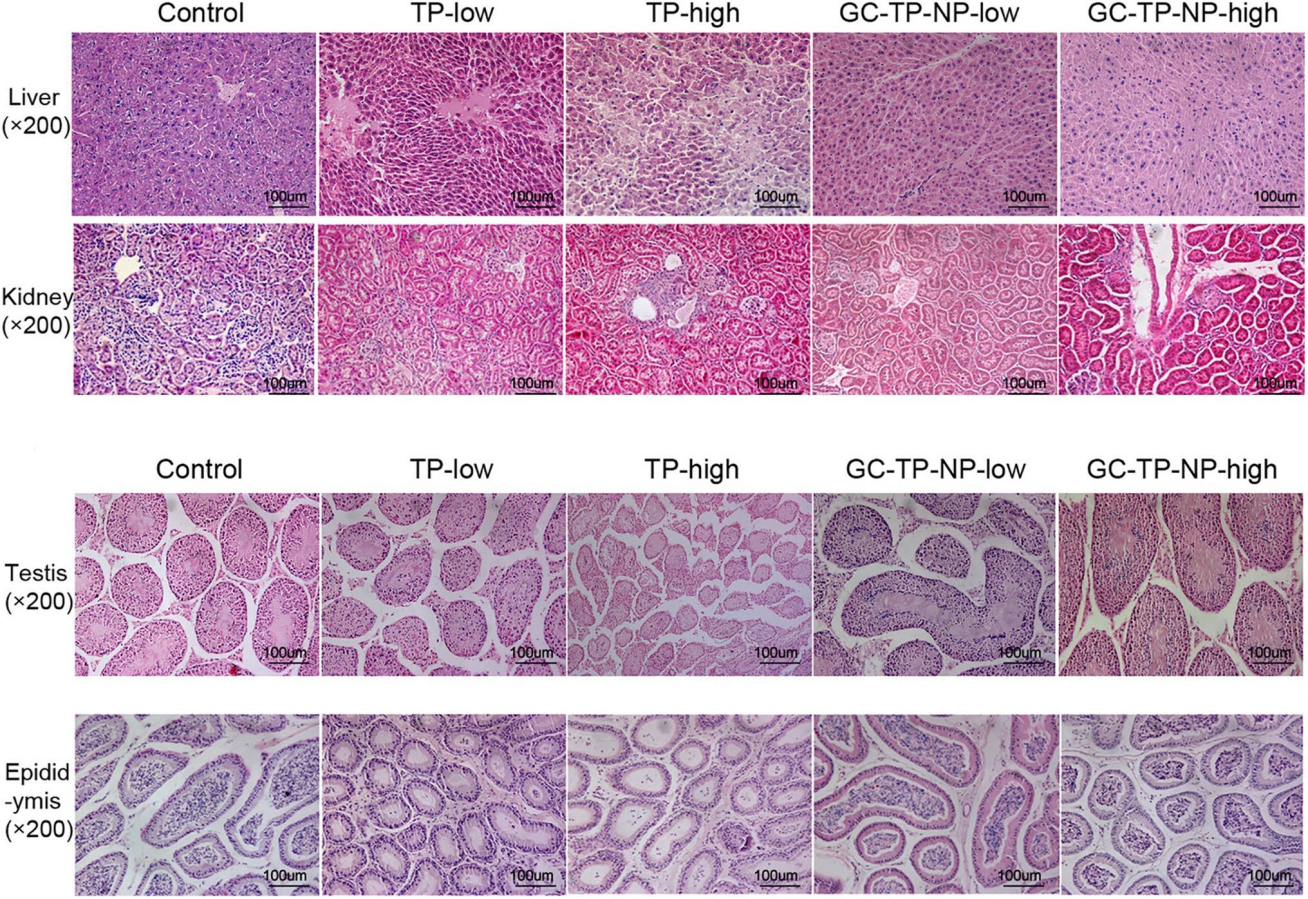
The encapsulation of TP into galactosylated-chitosan NPs decreased the hepatic, renal and male reproductive toxicity of this plant compound, as it is possible to observe comparing the histopathological images of liver, kidney, testis, and epididymis of mice treated low and high doses of TP (1 and 2.5 mg/kg, respectively) and Triptolide NPs (6.76 to 16.90 mg/kg, respectively). Overall, the encapsulation of TP into the galactosylated-chitosan NPs reduced the anomalous alterations the glomerulus and renal tubes, the cell swelling, vacuolar degeneration and necrosis in liver tissue, the damage in seminiferous tubules, with necrotic germ cells and deficient spermatogenesis. Adapted with permission [297]. Copyright 2019, Elsevier B.V. NPs: nanoparticles; TP: triptolide

The loading of TP within galactosylated-chitosan NPs have also demonstrated to reduce the toxicity of this plant compound while increasing its anticancer properties. The galactosylation of the chitosan NPs improved asialoglyco protein receptor mediated cellular uptake, inducing apoptosis in SMMC-7721 hepatocellular carcinoma via blocking TNF/NF-kB/BCL2 signaling, inhibiting inhibited the expression of TNF, NFKB1, NFKB1A, RELA, BCL2 and XIAP proteins. Also, in vivo* studies* on orthotopic xenograft hepatocellular carcinoma mice models demonstrated that the TP encapsulation into the galactosylated-chitosan NPs reduced its hepatic, renal and male reproductive toxicities (Fig. 15), while treatment with free drug led to a significant weight loss and toxicity, while having similar anticancer properties [297].

### Polyphenols

Polyphenols are natural compounds that exist in herb plants, vegetables, and fruits [298] and contain a minimum of one aromatic ring and hydroxyl groups [253, 298]. Polyphenols have presented therapeutic properties against chronic diseases, as well as cancer [253]. Several polyphenols induce apoptosis in cancer cells by mobilizing copper ions bound to chromatin, causing DNA fragmentation [248]. However, polyphenols also have poor stability and water solubility, lack of targeting towards cancer cells and low bioavailability, which prevents their anticancer efficacy. Therefore, their loading into nanocompounds, such as nanogels, polymeric NPs, liposomes and micelle, have improved their anticancer efficacy, increasing their targeting ability and aqueous solubility as well as enabling both an chemotherapeutic and immunomodulatory effect towards cancer [298].

### Honokiol

Honokiol (HK) is a biphenolic compound isolated from Magnolia with the root and stem bark of *Magnolia officinalis* [299, 300] and has been use for several pathologies in Chinese traditional medicine [299], presenting anti-inflammatory, antioxidant, antidepressant, neuroprotective and anticancer properties [299, 300], being able to suppress tumor growth and metastasis of several cancer types, such as brain, colon, liver, lung and skin [300]. Recent studies have demonstrated the potential of HK when encapsulated into NPs. For examples, Yang et al. developed polymeric NPs composed of copolymerpoly(e-caprolactone)-poly (ethyleneglycol)-poly (e-caprolactone) and functionalized with folic acid. The resultant nanocompound presented inhibitory properties against HNE-1 nasopharyngeal carcinoma cancer cells, with an IC_50_ of 18.41 µg/mL, while free HK presented an IC_50_ of 38.59 µg/mL. In vivo studies also demonstrated that the loaded polymeric NPs induced G1 cell cycle arrest and improved mice survival rate, by preventing tumor growth and angiogenesis [299].

**Fig. 16:**
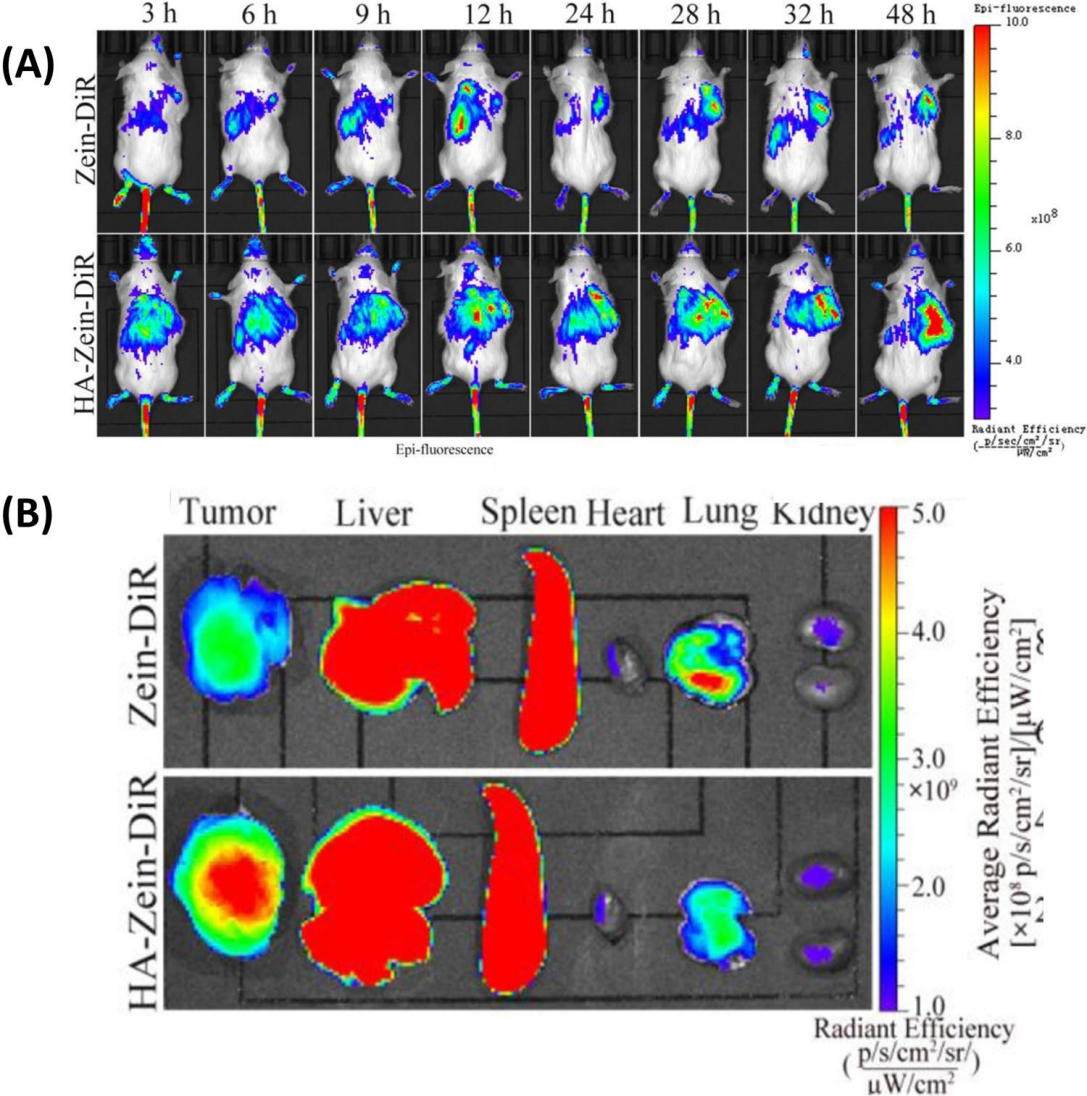
In vivo biodistribution studies of the several loaded nanoparticles produced using a fluorescent dye (DiR): **A** In vivo fluorescence images of 4T1 breast tumor-bearing BALB/c mice at different timepoints, in which it was possible to observe that while NPs functionalized with HA presented fluorescence at the tumor site at 24 h post administration, which lasted until the experiment ended, the fluorescent signal of the non-functionalized NPs appeared at 28 h after administration and the signal was much weaker; **B** Ex vivo fluorescence images of tumors and organs emphasising that the intratumoral delivery of the functionalized NPs was much higher and extensive than the non-functionalized NPs, although a relevant NPs accumulation was visible for both groups. Reprinted with permission [300]. Copyright 2020, Elsevier B.V. HA: hyaluronic acid; NPs: nanoparticles

In further studies, Zang et al*.* encapsulated HK into cationic NPs composed of zein, an amphiphilic protein from corn with biodegradable and biocompatible properties, and the targeting agent HA. The cationic NPs presented a slow sustained release of HK and increased antitumor properties towards 4T1 triple negative breast cancer cells, with and IC_50_ of 2.36 ± 0.85 μg/mL, while free HK presented an IC_50_ of 10.05 ± 1.19 μg/mL. The loaded cationic NPs inhibited the motility of cancer cells by downregulating vimentin expressions, while upregulating the expression of E-cadherin, as well as inhibiting Bcl-2 and increasing the levels of Bax. In vivo studies also demonstrated their inhibitory properties towards 4T1 tumor spheroids and improved tumor targeting ability, with improved accumulation into the tumor site (Fig. 16), reducing the tumor growth [300].

Zhang et al*.* also studied the anticancer potential of HK against 4T1 triple negative breast cancer cells, by encapsulating this plant compound into a nanocomposite with an inner layer of PBAE and an outer layer of FA-DSPE-PEG2000 to confer active targeting abilities. The produced NPs presented pH-dependent behavior and increased HK uptake by the 4T1 cancer cells, resulting in an IC_50_ value of 2.07 μg/mL, while free HK presented an IC_50_ of 7.08 μg/mL. Besides, FA/PBAE/Hol-NPs presented antimigration and anti-invasion properties and, when administrated at 4T1 bearing mice, inhibited the MMPs pathway, decreasing the tumor growth and suppressing the appearance of lung metastases, without visible damage in the major organs [301].

### Resveratrol

Resveratrol (3,5,40-trihydroxystilbene) (RES) is a plant phytoalexin which can be found in foods including peanuts, raspberries, mulberries, blueberries, knotweed, grapes and red wine [248, 250, 302], being that red grapes contain around 50–100 µg per gram in their barks [303]. RES has shown to have antioxidant, antitumor, antimicrobial, antiviral, anti-inflammatory, neuroprotective and cardioprotective properties [302–304], being that the trans isomeric form is more biologically active than the cis counterpart [305]. However, although RES has demonstrated therapeutic properties against skin, breast, prostate, and colon cancer, among others [306], and shown to reverse multidrug resistance in cancer cells [305], its low aqueous solubility, high photosensitivity, suffering rapid oxidation, low bioavailability limits its therapeutic applications [302, 304]. Therefore, several studies have encapsulated RES into NPs, such as liposomes, micelles, SLNs and metallic NPs, to improve its anticancer properties [306]. For example, Carletto et al*.* have demonstrated that the encapsulation of RES poly(ε-caprolactone) nanocapsules increased its therapeutic efficiency against murine melanoma, reducing the tumor growth, while increasing the necrotic areas and the inflammatory infiltrate compared to free drug, as well as prevented metastasis and pulmonary hemorrhage [302]. The encapsulation of RES within MSMs has also induced increased cytotoxicity against both A375 and MNT-1 melanoma cells. The RES-MSMs had pH-dependent behavior, since RES release was faster at pH 5.2 (acid environment in some tumorous tissues) than at pH 7.4 and, after 72 h incubation, presented an IC_50_ of 25.5 and 29.5 µg/mL in MNT-1 and A375, respectively [305].

Further studies have RES into SLNs against breast cancer [307, 308]. Wang et al. encapsulated RES into SLNs prepared by the solvent injection method using stearic acid and lecithin. The resultant RES-SLNs presented higher antiproliferative properties towards MDA-MB-231 cells, with and IC_50_ of 40.82 ± 3.92 µg/mL, while free RES presented an IC_50_ of 72.06 ± 7.85 µg/mL. RES-SLNs inhibited the invasion and migration of MDA-MB-231 cells and induced apoptosis and G0/G1 cell cycle arrest by increasing Bax/Bcl-2 ratio and downregulating cyclinD1 and c-Myc [309]. A recent study used D-α-Tocopheryl polyethylene glycol 1000 succinate (TPGS) for the preparation of SLNs. The resultant TPGS-RES-SLNs demonstrated to be cytotoxic towards SKBR3/PR breast cancer cells, by reducing P-gp mediated drug efflux, and inhibiting cell migration and invasion, via N-cadherin, vimentin and MMPs downregulation. TPGS-RES-SLNs also induced G2/M cell cycle arrest and mitochondria-mediated apoptosis and decreased the tumor growth in SKBR3/PR subcutaneous bearing nude mice, without damaging vital organs [308].

**Fig. 17:**
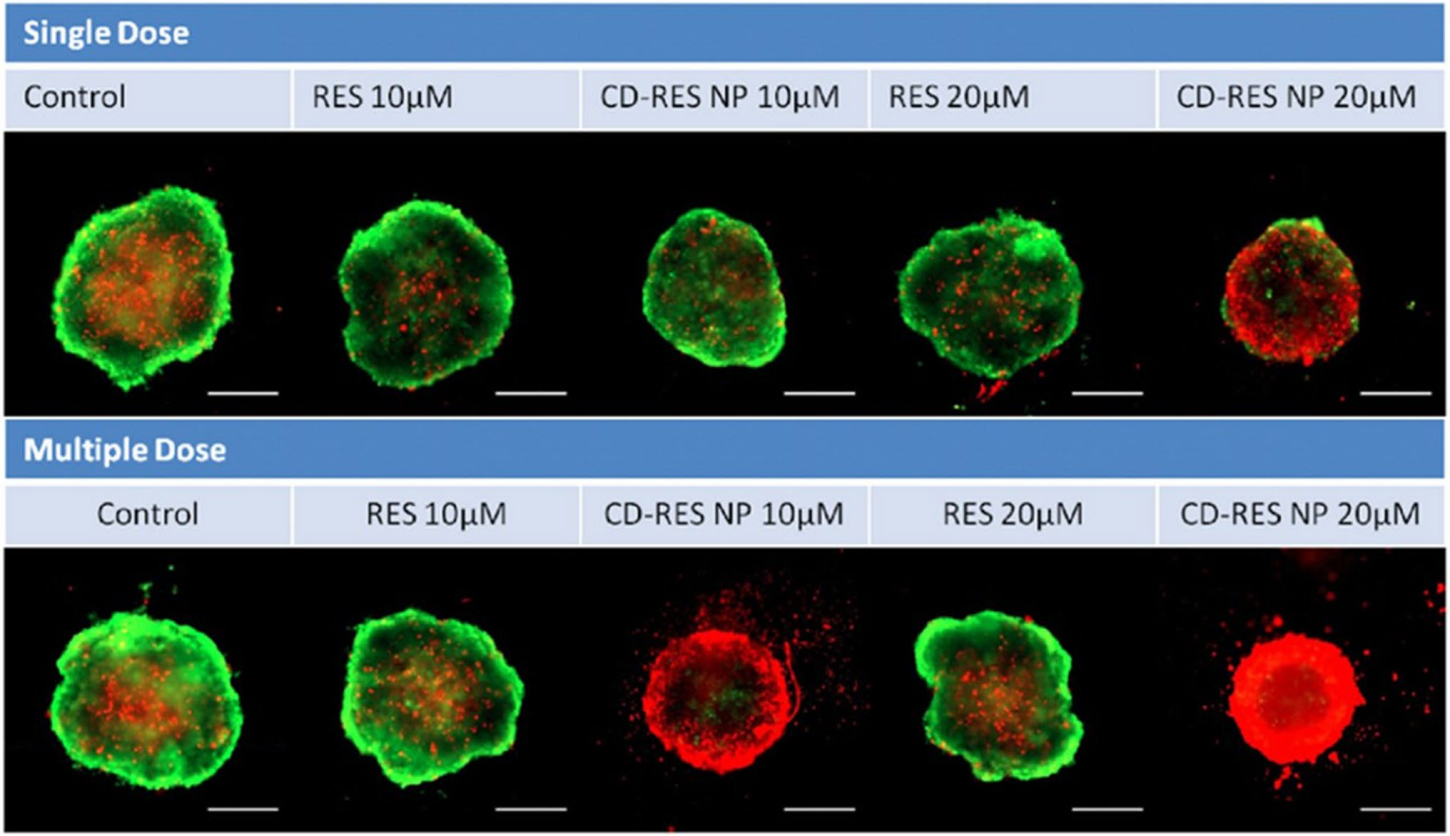
Images (20 × magnification) of A549 3D spheroids treated with single or multiple doses of Res and CD-Res NPs and further stained with fluorescent filters: green fluorescent protein (imaging calcein AM) for live cells; and red fluorescent protein (imaging EthD-III), for dead cells. It was possible to observe that, for both single- and multiple- doses, the spheroids treated with CD-Res NPs presented an increased intensity of red fluorescent protein (higher number of dead cells) compared to control and both concentrations of Res. Reproduced with permission [304]. Copyright 2020, ElsevierB.V. CD: cyclodextrin; NPs: nanoparticles; Res: resveratrol

RES have also presented therapeutic properties towards pancreatic cancer when loaded human serum albumin (HSA) NPs functionalized by the RGD peptide. RGD-RES-HAS NPs not only demonstrated antiproliferative properties in vitro against PANC-1 pancreatic cancer cells compared to free RES, but also prolonged RES blood circulation time and reduced the tumor growth in mice bearing PNAC-1 cancer, without relapse episodes and significant systemic toxicity during the 35 days treatment [307]. Also, Wang et al*.* have shown that the encapsulation of Res into CDs, cyclic polysaccharides composed of glucopyranose units with a cone-like structure, increases Res water solubility and its therapeutic properties against non-small cell lung cancer. Therefore, while Res-loaded CDs presented an IC_50_ of 3.3 ± 1.4 μM for A549 lung cancer cells, the free Res presents an IC_50_ of 50.8 ± 10.0 μM. The encapsulated Res presented antimigratory and antioxidant properties, while increasing caspase-3 activities, decreasing the volume of tumor spheroids as well (Fig. 17) [304]

### Curcumin

Curcumin (Cur) is a hydrophobic polyphenol extracted from the rhizome of *Curcuma longa*, commonly known as turmeric, which is mainly used as an yellow food colorant and spice [249, 250, 310]. Although Cur has shown anticancer properties, its physical instability, poor aqueous solubility and rapid metabolism limits its therapeutic application, being that the biocompatible and biodegradable NPs have been used to enhance its aqueous solubility, stability, bioavailability and targeting to cancer cells [250, 310, 311]. For example, polymeric NPs prepared using poly(glycerol sebacate) presented increased cytotoxic properties against HeLa cervical cancer cells compared to free Cur, by increasing cleaved caspase-3 and PARP levels and inducing cell cycle arrest and apoptosis [312]. Also, niosomes (Nio) prepared using cholesterol and PEG and loaded with Cur demonstrated cytotoxic properties against sw-1736 thyroid cancer cells. The treatment with Cur-Nio resulted with increased bax/bcl2 ratio and overexpression of caspase-3 and caspase-8, resulting in cell shrinkage and appearance of apoptotic bodies [313]. Other in vitro study demonstrated Nio potential to improve Cur anticancer effect. Cur-Nio presented toxicity towards glioblastoma stem-like cells, by increasing intracellular ROS and upregulating Bax, while decreasing NF-kB and IL-6 expressions, inducing sub-G1 cell cycle arrest and both apoptosis and necrosis. Besides, Cur-Nio prevented colony formation and inhibited the migration and invasion of cancer cells, by suppressing MMP-2, as well as their invasiveness via monocyte chemoattractant protein-1 mediated pathways. Besides, the expression of several cancer stem cell markers, such as Sox2 and nestin, was substantially reduced [314].

**Fig. 18:**
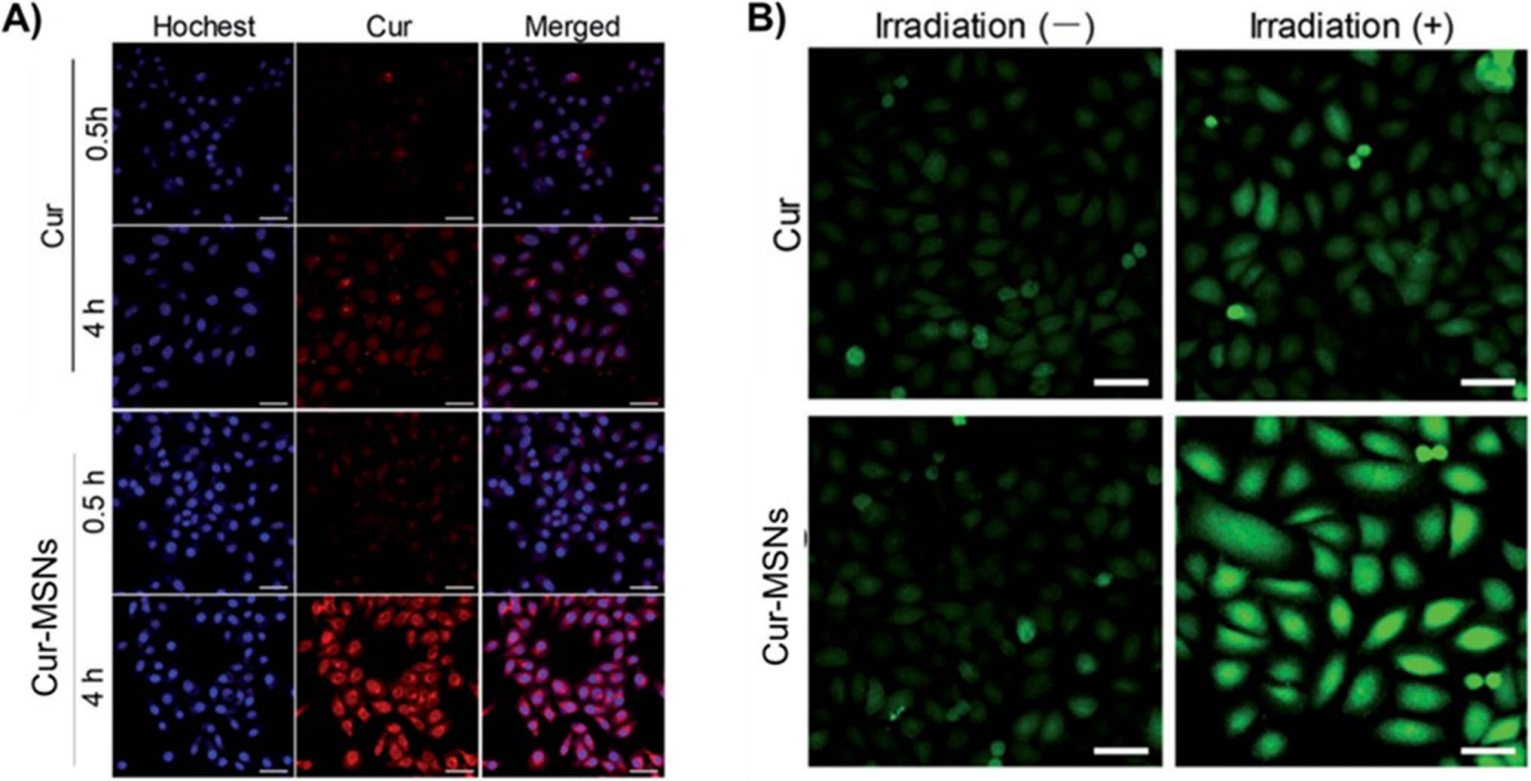
Intracellular behavior of Cur and Cur-MSNs in Hela cells. **A** Cur poor solubility prevents its cell internalization (red fluorescence), while its internalization into MSNs increased its internalization after 4 h of incubation. **B** Therefore, the generation of reactive oxygen species (ROS) in Hela cells incubation after incubation with Cur-MSNs was increased compared with free Cur, especially when combined with irradiation (430 nm, 20 mW cm.^−2^). Reproduced with permission [311]. Copyright 2020, RSC Publishing. Cur: Curcumin; MSNs: mesoporous silica NPs; NPs: nanoparticles

The encapsulation of Cur into magnetite NPs functionalized with FA improved the internalization in folate receptor-positive MCF-7 human breast cancer cells, improving Cur anticancer properties [315]. The FA functionalization have also improved the targeting of Cur-NioNPs to cancer cells. FA-Cur-NioNPs induced apoptosis on both MCF-7 and 4T1 breast cancer cells, with an IC50 of 47.75 ± 0.82 and 69.88 ± 2.70 µg/mL after 72 h incubation, respectively. FA-Cur-Nio increased ROS amount and induced the upregulation of both Bax and p53, inducing sub-G1 cell cycle arrest and apoptosis [310]. In vivo studies concerning a HeLa cervical carcinoma xenograft model have also demonstrated the FA potential to targeting NPs encapsulating Cur towards cancer cells. FA-β-CD-polycaprolactone polymeric NPs presented pH-responsive behavior and increased Cur accumulation in the tumor site, decreasing the tumor growth when compared with free Cur and non-functionalized Cur-NPs (Fig. 18) [316].

Polymeric NPs have also demonstrated to increase Cur anticancer properties against osteosarcoma, by increasing the Cur uptake by mesenchymal stromal cells, a class of multipotent progenitor cells that reside mostly in the bone marrow and modulate the immune system to enable tumor growth, metastasis, and drug resistance. The encapsulated Cur presented higher anti-inflammatory properties than free Cur, preventing the secretion of IL-6/8, reverting the pro-tumorigenic phenotype of MSCs, and decreasing the tumor stemness, migration, and invasion during photogenic and migration assays [317]

Cur encapsulation into lipid NPs have also increased its anticancer potential [318, 319]. For example, Luo et al*.* produced lipid NPs functionalized by a specific peptide targeting nasopharyngeal cancer to encapsulate Cur. The lipid NPs increased Cur uptake into 5-8F cancer cells compared to free Cur, resulting in inhibition of the motility and increased apoptosis, with an IC_50_ of 104.1 ± 2.5 μM. Also, in vivo studies using a nasopharyngeal cancer xenograft tumor model demonstrated that the nanosystem inhibited the tumor growth and blocked lung metastasis without visible side effects, while mice treated with free Cur presented several metastatic lung nodules [319]. More recently, Wei et al. verified the increased therapeutic effect of Cur when encapsulated into SLNs. Cur-SLNs presented increased uptake efficiency to hepatocarcinoma MHCC-97H cells, inhibiting their proliferation, migration, and invasion potential by downregulating NF-kB, COX-2, MMP-2, MMP-9, and uPA pro-cancer proteins. Besides, SLNs enhanced Cur pharmacokinetic profile, extending its retention time and increasing the area under the curve, without significant liver toxicity [318].

Silica-based NPs have been also studied for Cur encapsulation to improve its anticancer efficacy [310, 320, 321]. Ghazaeian et al. produced a Cur-Silica nanocomplex with photodynamic properties under blue LED (465 nm; power density: 34 mW/cm2), inducing cytotoxicity in melanoma A375 cancer cells, without effecting human normal fibroblasts. Cur-Silica NPs presented better photodynamic properties than free Cur, causing ROS production and nuclei damage, with inhibition of colony formation and increased apoptosis [320]. Furthermore, MSNs have also demonstrated to increase Cur uptake into cancer cells. For example, the encapsulation of Cur into MSNs which surface was modified with alginate oligosaccharides improved its therapeutic potential against HCT-116 colon cancer cells [321]. Furthermore, rod-shaped Cur-MSNs have presented higher cytotoxicity against HN5 head and neck cancer cells compared to free Cur, increasing ROS production, and reducing Bcl.2 expression, resulting in IC50 levels of 16 µM, 11.93 µM in 24 and 48 h, respectively (64). Additionally, Kuang et al. demonstrated the photodynamic potential of Cur encapsulated into PEGylated MSNs. The resultant Cur-MSNs could quickly generate ROS specially upon irradiation in Hela cancer cells, resulting in increasing in vitro cytotoxicity compared with free Cur, with IC50 values of 5.5 mg/mL and 22.9 mg/mL, respectively (Fig. 18) [311].

Cur-loaded NPs have also presented potential for photothermal [322] and ultrasound anticancer therapy [323]. Howaili et al*.* produced a cross-linked nanogel composed of poly (N-isopropyl acrylamide) and chitosan, to which Cur-AuNPs were further incorporated. The Cur-AuNPs uptake was more efficient in MDA-MB-231 cancer cells than in non-cancer cells and, and after exposed to NIR irradiation (808 nm) for 10 min, AuNPs converted the light into heat, which resulted in nanogel shrinkage and Cur release. Therefore, Cur-AuNPs presented synergy response with the NIR laser exposure in reducing the viability of MDA-MB-231 cancer cells [322]. Additionally, Guo et al*.* produced ferritin nanocages to encapsulate both Cur and liquid fluorocarbon perfluorohexane and evaluated the therapeutic potential of this nanosystem against SKOV-3 ovarian cancer cells when exposed to low-intensity focused ultrasound. Overall the nanosystem presented pH-dependent release and presented synergic activity with the use of ultrasound to inhibit the viability of cancer cells in vivo and the tumor weigh in vivo, at the same time that induced an contrast image of the tumor site, demonstrating to be a viable theragnostic option [323].

### Flavonoids

Flavonoids are a large family of plant bioactive polyphenolic metabolites [248, 324], which main sources are fruits and vegetables, especially plums, cherries, apples, spinach, and onions. Due to their potential as ROS modulators and to interfere in the cell cycle, flavonoids have been studied as anticancer compounds, capable of suppressing cancer cell proliferation [324, 325]. Due to their low bioavailability and lack of targeting towards the cancer cells, nanotechnology has been used to enhance their water solubility and tumor specificity to improve effectiveness of this class of biocompounds [325] (Table 7).

**Table 7:** Characteristics and anticancer properties of NPs loaded with of flavonoids

NPs properties	Cancer cell type	Dosage	Biochemical mechanism	Effect on target cells	Reference
Baicalin					
Dendrimers Size: 247.5 nm ZP: -9.0 mV	Cervical (Hela) and Lung (A549) cancer	IC_50_ = 7.49 µg/mL	-	↑ Apoptosis and G1 phase cell cycle arrest	[326]
AuNPs Size: 30 nm	Breast Cancer (MCF-7)	-	↑ Caspase-3 and apaf-1	↑ Nuclear Fragmentation ↑ Apoptosis	[327]
Polymeric NPs Size: 182.6 ± 3.8 nm ZP: -13.8 ± 0.3 mV	Melanoma (B16)	-	M1/M2 macrophage polarization ↑ Infiltration of CD4 + T and CD8 + T cells	↓ Tumor Growth	[328]
Chrysin					
Polymeric NPs Size: 20—75 nm	Gastric cancer (AGS)	IC_50_ values (µM): 24 h – 58.24 48 h – 44.21 72 h – 36.80	↑ MicroRNAs 22, 34a and 126	↓ Cell Viability	[329]
Polymeric NPs	Breast cancer (4T1)	In vitro studies: IC_50_ value = 45 µM In vivo studies: 25 mg/kg b.w. i.p	↓ MMP-2/9	↓ Tumor size	[330]
Zinc Oxid NPs Size: 415.1 nm ZP: -17.36 mV	Lung cancer	IC_50_ = 223.42 µg/mL	↓ Bcl-2 ↑ ROS, Bax and caspases-3/9 ↑ p53 ↓ cyclin D1 ↓ MMP-2 and VE-cadherin	G0/G1 cell cycle arrest ↑ Apoptosis ↓ Cell migration and invasion	[331]
Genistein					
AuNPs Size: 65.0 ± 1.7/ 153.0 ± 2.2 nm ZP: 35.0 ± 2.5/ 37.0 ± 1.6 mV	Prostate cancer (LNCaP, DU 145, PC3)	In vitro studies (µg/mL and 72 h treatment) LNCaP – 19.6 ± 0.1/ 29.3 ± 5.1 DU 145 – 39.6 ± 0.6/ > 50 PC3 – 22.6 ± 1.6/ 46.3 ± 1.1	-	↓ Cancer cell viability ↑ Viability of non-cancer cells	[332]
NPs Size: 150 nm ZP: negative	Oral squamous cell carcinoma (JHU011)	IC_50_ = 40 µM	↑ ROS, Bax, caspase-3, PP2A-Cα/β ↓ Bcl-2, Bmi-1 and EZH2	G0/G1 cell cycle arrest ↑ Apoptosis	[333]
Luteolin					
ROS-responsive NPs Size: 196.7 ± 1.8 nm ZP: -41.0 ± 0.01	Breast cancer (4T1)	In vitro studies: IC_50_ value = 39.9 µM In vivo studies: 10 mg/kg b.w. i.v	↓ MMP-9	↓ Tumor growth and size	[334]
Ethosomal NPs Size: 267 ± 8.6 nm ZP: -42.6 ± 3.01	Hepatocellular Carcinoma (N-Nitrosodiethylamine induced)	100 mg/kg b.w verbal gavage, for four weeks	↓ Hepatic GPC3 expression ↓ ALP, bilirubin and AFP-L3, ↓ Nitric Oxide and malondialdehyde ↑ GSH and SOD	↓ Neoplastic hepatic injuries ↓ Hepatic adenomas	[335]
Phloterin					
Chitosan NPs Size: 80.21 nm ZP: 55.06 mV	Papilloma (KB)	IC_50_ = 21.34 ± 0.48 μg/mL	↑ ROS, lipid peroxidation ↓ SOD, CAT, and GSH Mitochondria dysfunction ↑ PARP, BAX cytochrome c, APAF, and caspases-3/9 ↓ Bcl-2	Changes in cell morphology G0/G1 cell cycle arrest Intrinsic apoptosis	[336]
	Oral squamous cell carcinoma (DMBA induced)	20 mg/kg b.w. oral	↑ ROS ↓ SOD, CAT, GSH, vitamin C and E ↑ BAX cytochrome c, APAF, and caspases-3/9 ↓ Bcl-2	↓ Tumor volume and burden ↓Buccal damage ↓Collagenous fibers	[337]
Chitosan-PLGA NPs Size: 342 ± 39 nm ZP: 23.3 ± 2.4 mV	Melanoma (B16F10)	In vitro studies: IC_50_ value = 87.5 ± 5.8 µg/mL In vivo studies: 5 mg/kg b.w. i.v 5 mg/kg) days 3, 7, 9, 11, and 15	-	↓ Metastasis	[338]
Quercetin					
PLGA NPs Size: 89.8 ± 5.9 nm ZP: 19.8 mV	In vitro: Cervical cancer (HeLa) In vivo: Breast cancer (DMBA induced)	128 mg/kg b.w i.p	In vitro: Mitochondria dysfunction ↑ Caspases-3/7 ↓ PI3K/AKT pathway	In vitro: G2 cell cycle arrest Apoptosis and necrosis In vivo: ↓ Tumor weight and volume	[339]
Size: 114.6 ± 6.22 nm ZP: 19.8 ± 0.75 mV	Lung (A549) and breast (MDA MB 468) cancer	25 mg/kg b.w. i.v 2x/week for 4 weeks	-	↓ Tumor volume	[340]
Hydroxyapatite NPs Size: 32.2 ± 5.9 ZP: − 19.3 ± 0.1 mV	Cervical cancer (HeLa)	IC_50_ = 200 μM quercetin	↑ ROS	↑ Apoptosis	[341]
Rutin					
Chitosan/poly (acrylic acid) nanogel Size: 81.9 nm	Hepatocellular Carcinoma (Diethylnitrosamine induced)	50 mg/kg b.w. oral (6 weeks)	↑ Bax, caspase-3 and p53 ↓ Bcl-2, VEGF	↓ Tumor Growth ↓ Hepatocellular Damage	[342]
Liquid crystalline NPs Size: 160 nm ZP: less than -25 mV	Lung Cancer (A549)	IC_50_ = 35.44 µM	↓ MMP-9	Anti-colony and anti-migratory properties ↑ Apoptosis	[343]

*ALP* alkaline phosphatase, *b.w.* body weight, *CAT* catalase, *DMBA* 7,12-dimethylbenz[a]anthracene, *GSH* glutathione, *IC*_*50*_ half maximal inhibitory concentration *i.p.* intraperitoneally, *IL* Interleukin, *i.v.* intravenous, *MMP* metalloproteinase, *NPs* nanoparticles, *ROS* reactive oxygen species, *SOD* superoxide dismutase, *VEGF* vascular endothelial growth factor, *ZP* ζ-potential

For example, baicalin (BAI) is a flavonoid extracted from the root of *Scutellaria baicalensis* Georgi [326, 328], and has shown anticancer properties on bladder, lung, breast, prostate, colorectal, ovarian and liver cancer cells [326]. Lv et al*.* demonstrated that the encapsulation of this biocompound in dendrimers composed of polyamidoamine and functionalized by FA improved its anticancer properties on Hela cancer, inducing G1 phase cell cycle arrest [326]. Also, Lee et al*.* used AuNPs conjugated with thiolated beta-CD as a BAI delivery vector to improve its therapeutic properties against MCF-7 breast cancer cells. The treatment with this nanosystem resulted in higher expressions of pro-apoptotic makers caspase-3 and apaf-1, indicating apoptosis with nuclear fragmentation [327]. BAI has also presented immunotherapeutic properties when encapsulated with PLGA polymeric NPs containing a Hgp antigenic peptide and a toll-like receptor 9 agonist and coated with red cell membranes functionalized with galactose to target tumor-associated macrophages. Overall, this nanosystem induced the polarization of M2-procancer macrophages to the M1-anticancer macrophages both in vitro and in vivo, as well as increased the infiltration of CD4 + T and CD8 + T cells into tumor sites, suppressing melanoma growth in mice [328].

Chrysin (5, 7-dihydroxyflavone) is another flavonoid with anticancer properties which encapsulation of NPs have been shown to increase its effectiveness in both gastric [329], breast [330] and lung cancers [331]. Mohammadian et al*.* study the potential of PLGA-PEG NPs to encapsulate and improve chrysin antitumor properties. The encapsulated chrysin presented higher toxicity than free chrysin against AGS gastric cancer cells, by inducing the expression of pro-apoptotic MicroRNAs 22, 34a and 126 [329]. The encapsulation of chrysin in PLGA-PEG NPs has also shown to improve its anticancer properties against 4T1 breast cancer cells, both in vitro and in vivo, by reducing both MM2 and MM9 expressions, therefore reducing the tumor size in mice in comparison with free chrysin [330]. More recently, Mahalanobish et al*.* encapsulated chrysin in zinc oxide NPs functionalized with phenylboronic acid. The resultant nanosystem targeted A549 lung cancer cells via sialic acid receptors, which are overexpressed on their cell surface, and induced oxidative stress, with downregulation of Bcl-2 and overexpression of Bax and caspases-3/9. Also, p53 was activated and the expression of cell regulatory cyclin D1 was decreased, leading to G0/G1 cell cycle arrest and intrinsic apoptosis. Furthermore, the nanosystem also decreased cell migration and invasion via downregulation of MMP-2 and VE-cadherin, respectively [331].

More recently, the encapsulation of genistein (Gen) has been studied for anticancer therapy as well. This isoflavone, present in plant species of the *Leguminosae* family, especially soybean, has strong anti-proliferative properties [250, 332]. Therefore, Vodnick et al*.* encapsulated Gen into two types of AuNPs and demonstrated their potential against several types of prostate cancer cells, without inhibiting non-cancerous MRC-5 cells [332]. Additionally, Dev et al*.* produced Gen NPs using lactalbumin via antisolvent precipitation and studied their potential against JHU011 oral squamous cell carcinoma cells. The treatment with Gen NPs enhanced ROS production, leading to decreased Bcl-2 expression and Bax and caspase-3 overexpression. Also, the expression of the apoptotic marker PP2A-Cα/β was increased, while the epigenetic regulators Bmi-1 and EZH2 were downregulated through proteasomal mediated degradation and 3PK inhibition, leading to a decreased expression of their respective products, UbH2AK119 and H3K27me3. Therefore, Gen NPs induced G0/G1 cell cycle arrest and increase the apoptosis of cancer cells [333].

Luteolin (Lut) is another flavonoid present in several fruits, vegetables, and medicinal herbs, that presents several therapeutic properties, including antitumor effects against several types of cancer, by inducing cell cycle arrest and apoptosis, and inhibiting angiogenesis [334, 335]. However, due to its hydrophobic nature, Lut also presents limited therapeutic applications due to its poor solubility, which limits its oral bioavailability [335]. To improve its anticancer potential, Wang et al*.* encapsulated Lut into NPs produced using lecithin and 1,2-Distearoyl-sn-glycero-3-phosphoethanolamine-N-methoxy(polyethylene glycol)-2000 and containing, as well as CD modified by 4-(hydroxymethyl) phenylboronic acid pinacol ester as a ROS-responsive biomaterial. The resultant nanocompound was functionalized with FA to increase its targeting ability towards 4T1 breast cancer cells in vitro and in vivo, accumulating preferentially at the tumor site. The nanosystem mechanism of action involved the reduction of MMP-9 expression, inhibiting tumor growth 3 times more compared with free Lut, without significant side effects [334]. Additionally, Elsayed et al*.* encapsulated Lut into ethosomal NPs by the cold method and demonstrated their potential against N-Nitrosodiethylamine induced hepatocellular carcinoma. It was observed that while the expression of the hepatic GPC3 gene was enhanced in carcinogenic groups and the treatment with free Lut did not change its expression, Lut-NPs significantly reduced GPC3 hepatic expression. Lut-NPs reduced both liver alkaline phosphatase (ALP), bilirubin and AFP-L3, a biomarker elevated in hepatic cancer. The liver oxidative environment was also reduced in groups treated with Lut-NPs, with reduction of nitric oxide and malondialdehyde, while the levels of the antioxidant compounds GSH and superoxide dismutase (SOD) were increased. Therefore, the treatment with Lut-NPs reduced the amount of neoplastic hepatic damage caused by N-Nitrosodiethylamine [335].

Phloretin is found in apple fruit and leaves, and the bark of pear and cherry and that has several medicinal properties, including antitumor activities against several types of cancer cells [336]. However, this biocompounds also presents poor aqueous solubility, being that its encapsulation into several types of NPs have been studied. For example, Mariadoss et al*.* studied the anticancer potential of phloretin loaded into chitosan NPs against KB cells. The encapsulated phloretin induced ROS generation and lipid peroxidation, while decrease the levels of the antioxidant enzymes SOD, catalase (CAT), as well as GSH. Due to the exacerbated oxidative environment, the mitochondrial membrane potential was reduced, inducing downregulation of Bcl-2 and increased activity of PARP, BAX cytochrome c, APAF, and caspases-3/9, which are characteristic of the intrinsic apoptosis pathway. Therefore, the treated cells presented cell cycle arrest in G0/G1 phase, with apoptotic and necrotic characteristics, including cytoplasmic condensation and fragmented nuclei with chromatin condensation [336]. The anticancer potential of phloretin encapsulated into chitosan NPs was also demonstrated against oral squamous cell carcinoma induced using 7,12-dimethylbenz[a]anthracene (DMBA). The treatment with encapsulated phloretin demonstrated the same trend as the previous study, decreasing the formation of oxidant species, and enhancing the level of antioxidant compounds, such as CAT, SOD, GSH, as well as vitamin C and E. Therefore, the encapsulated phloretin reduced the tumor volume and burden in the exposed hamster, inverting the buccal damage and the overproduction of collagenous fibers caused by DMBA [337]. Lee et al*.* also encapsulated phloretin into PLGA NPs decorated by chitosan to study its antitumor potential against melanoma lung metastasis. The produced nanosystem reduced the number of metastases, without presenting hepatic or renal side effects, demonstrating to be safe for intravenous administration [338].

Another flavonoid with proven antitumorigenic and antioxidant properties is quercetin, which is found in berries, caper, black and green tea leaves, buckwheat, onion, broccoli, apple, among other leafy green vegetables [249, 250]. However, quercetin also presents low aqueous solubility and intracellular absorption, as well as poor tumor-targeting [250, 339, 344], being that its encapsulation into NPs have been studied to enhance its therapeutic properties [250]. Yadav et al*.* demonstrated that PLGA NPs loaded with quercetin against were more cytotoxic to HeLa cancer cells than free quercetin. The encapsulated quercetin caused mitochondrial disfunction, inducing the activation of pro-apoptosis caspases-3/7, as well as inhibiting the pro-proliferation PI3K/AKT pathway, causing G2 cell cycle arrest, and provoking both apoptosis and necrosis. The quercetin-PLGA NPs also reduced the volume and weight of DMBA-induced mammary tumors in rats [339]. Other in vivo studies demonstrated the quercetin encapsulated into chitosan also shown therapeutic properties against both lung and breast cancer, reducing the tumor volume in A549 and MDA-MB-468 tumor bearing mice, respectively [340]. Additionally, Simon et al*.* encapsulated quercetin into hydroxyapatite NPs doped with copper nanoclusters. The nanosystem induced apoptosis in HeLa cancer cells via ROS generation, which hypothesized that, although quercetin commonly acts as an antioxidant, it can be a pro-oxidant at higher concentrations or in the presence of transition metals like copper [341].

Rutin is also a flavonoid present in apples, oranges, lemons, tomatoes, and tea, and presents chemopreventive properties in several types of cancers [342], although has poor aqueous solubility that limits its oral bioavailability, being that its encapsulation within NPs has shown to increase its efficacy [343]. Radwan et al*.* produced a chitosan/poly (acrylic acid) nanogel as a rutin deliver system for the treatment of chemically induced hepatocellular carcinoma. The encapsulated rutin presented better antitumor properties than the drug, normalizing the levels of alanine aminotransferase, ALP, gamma glutamyl transferase, and total bilirubin [342]. The oral delivery of this nanogel also resulted in upregulation of Bax, caspase-3 and p53 while the expression of antiapoptotic Bcl-2 was decreased. The nanogel also decreased the level of vascular endothelial growth factor, indicating the inhibitory propertied of rutin in angiogenesis. The histopathological observation liver sections also permitted to observe the rutin deceased the damage in the hepatocellular architecture induced by diethylnitrosamine [342]. Additionally, Paudel et al*.* encapsulated rutin into liquid crystalline NPs (LCNs) composed of monoolein and study its anticancer potential against A549 human lung carcinoma cells. Rutin-LCNs presented both anti-colony and anti-migratory properties, downregulating MMP-9, as well as induced apoptosis of cancer cells [343].

### Chlorophyll

Chlorophyll (CHL), an essential biomolecule for plant photosynthesis, is known for its therapeutic benefits, including antioxidant, detoxification and anticarcinogenic [345], although its hydrophobic nature results in reduced solubility in physiological media and reduced cellular uptake [345–347]. CHL has also been shown to be a promising photosensitizer for photodynamic therapy, due to its ability to absorb light at the range of 600–700 nm [346], with conversion of the triplet oxygen ^3^O_2_ to the reactive singlet oxygen (^1^O_2_), which is cytotoxic [347].

**Fig. 19:**
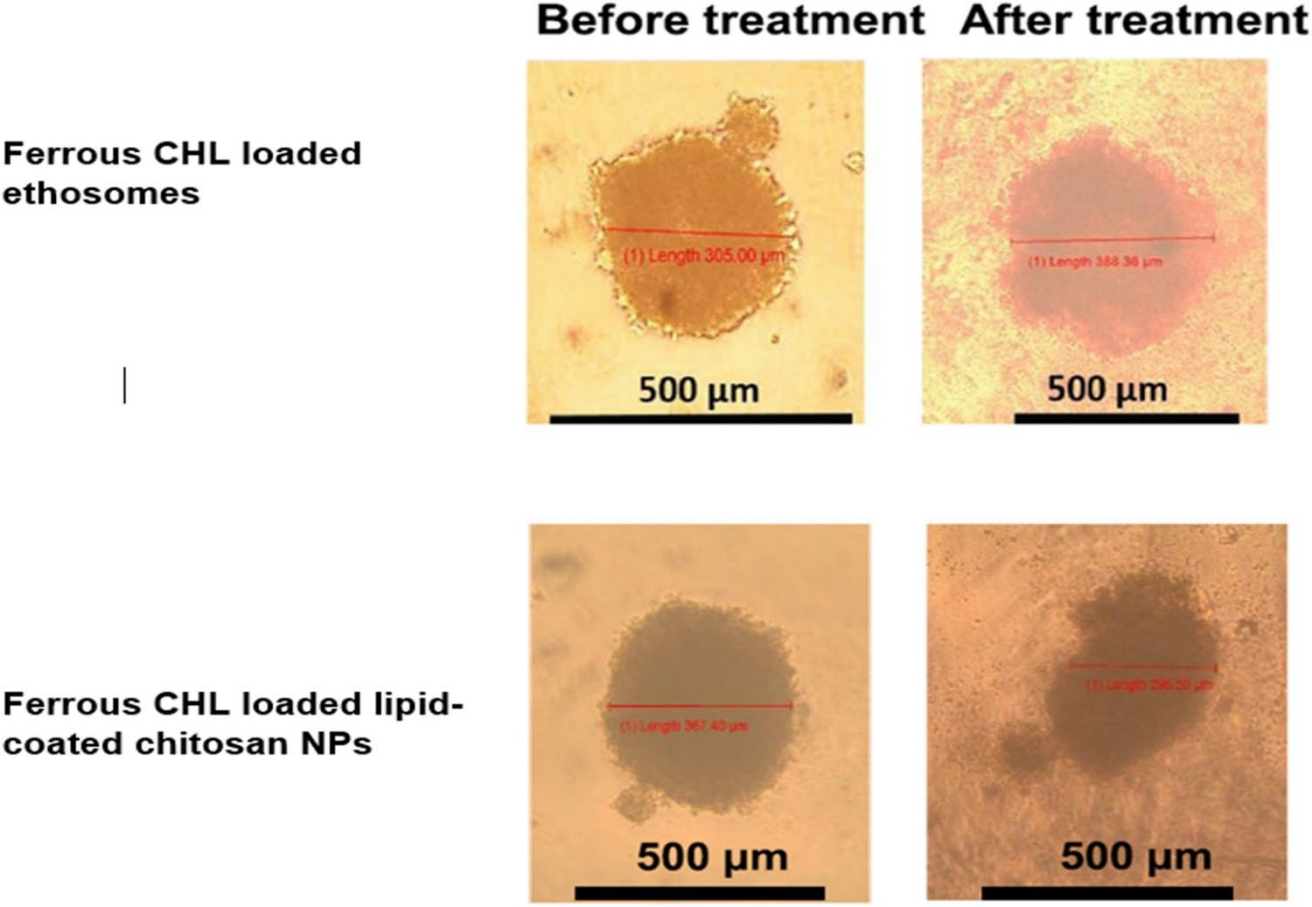
A431 spheroids PDT treated with ethosomes and lipid-coated chitosan NPs loaded with ferrous CHL before and after treatment. Reproduced with permission [348]. Copyright 2019, Elsevier B.V

Pemmaraju et al*.* loaded the CHL rich biomolecular fraction from the plant *Anthocephalus cadamba* and a near infra-red dye IR-780 into polymeric PLGA NPs. The polymeric NPs increased the dye stability to light, preventing photo bleaching, and increased the uptake of biomolecular fraction of *A. cadamba* into melanoma cancer cells, resulting in increased ROS production. Therefore, the resultant nanosystem presented both synergic photothermal and cytotoxicity properties towards cancer cells, in which the use of laser radiation results in local hyperthermia and increased release of the biomolecular fraction of *A. cadamba*. [345]. Furthermore, Nasr et al*.* have demonstrated the photothermal potential of CHL towards skin cancer, by loading a naturally derived photosensitizer, ferrous CHL into ethosomes and chitosan NPs coated by phosphatidylcholine and incorporating the nanosystems into a gel matrix. The gel formulations did not present cytotoxic effects, maintaining the skin structure before laser exposure. Also, the nanosystems increased the skin retention and penetration of CHL, specially ethosomes, while the chitosan NPs were more localized in the epidermis, without penetration into deeper skin layers. However, the loaded lipid-coated chitosan NPs presented much higher cytotoxicity towards A431 human squamous carcinoma cell than ethosomes, especially when a red laser was applied. The loaded chitosan NPs also presented synergy with laser treatment by decreasing the integrity of cell membrane in 3-D spheroids of A431 cancer cells (Fig. 19), indicating the potential of this nanosystem for photodynamic therapy [348].

AuNPs have also been shown to increase CHL photostability. While free until laser light, CHL molecules aggregated and lost energy through dipole–dipole energy transfer between the porphyritic macrocycles, being destroyed with ROS generation. However, AuNPs stabilized the CHL and preserved its optical properties. Therefore, AuNPs conjugated with CHL presented photodynamic therapy properties against both MCF7 and HelG2 cancer cells, whereas conjugated CHL was photoactive and photostable and, therefore, more efficient for photodynamic and photothermal therapy than the unconjugated [346].

NPs loaded with CHL have also presented potential for sonodynamic therapy, which uses low-intensity ultrasound (1–3 MHz) to activate a sonosensitizer agent, inducing selective cancer cell toxicity. The encapsulation of CHL into liposomes, SLNs or PLGA NPs, increased the toxicity towards prostatic cancer cells (PC-3), especially when used in concomitance with sonodynamic treatment, due to the overproduction of ROS. The synergy of the loaded NPs with the sonodynamic treatment was also observed in DU-145 spheroids, being possible to observe that the PLGA NPs presented with highest sonodynamic cytotoxicity, reducing the spheroid volume in 60% after 24 h incubation [349].

## Essential oils and plant extracts

Essential Oils (EOs) are odorant products obtained from a defined plant raw material as part of their secondary metabolism and are mainly composed of terpenes EOs can be obtained using several extraction techniques, such as and microwave-assisted processes and can be administrated through both external, oral, and topical routes [350]. Although several EOs presented anticancer properties, inducing ROS overproduction, modulation of tumor suppressor proteins, DNA damage and cell cycle arrest, they can also have several side effects, such as possible allergies, intoxication, phototoxicity and photosensitivity, abortion-provoking, nephrotoxic and hepatotoxic. Besides, EOs are also unstable, have a high volatility and deterioration risk and low water solubility, resulting in poor viability. Therefore, EOs loaded into NPs have been studied to reduce their side effects, increase their safety and stability, or have a controlled release. Several EOs loaded into nanocompounds have presented toxicity activity against MDA-MB-231 breast cancer cells, A549 human lung cancer cells and HepG2 liver cancer cell line by causing mitochondria dysfunction, without significant effect on normal cells [350].

For example, Xu et al*.* produced chitosan NPs loaded with *Cinnamomum cassia* EO, known to have high amounts in cinnamaldehyde, cinnamic acid, and coumarin, and to have analgesic, hypoglycemic, antibacterial, anti-depressant, anti-inflammatory, antioxidant, and chemotherapeutic properties. The loaded NPs presented increased cytotoxic and antimigratory properties against MDA-MB-231 breast cancer cells (IC_50_ of 25.24 ± 3.31 μg/mL) when compared with free EO (IC_50_ of 32.25 ± 4.72 μg/mL), by increasing ROS production and up-regulating the pro-apoptosis proteins caspase-3 and AIF. Further in vivo studies in mice injected with triple negative 4T1 breast cancer cells indicated that the chitosan NPs loaded with *Cinnamomum cassia* EO at a dose of 25 mg/kg reduced the tumor growth, however while inducing renal damage [351]. Also, Rashidipour et al*.* extracted EO from *Satureja khuzistanica jamzad*, a plant known to have antispasmodic, antibacterial, anti-inflammatory, antidiabetic, anticoagulant, antioxidant, and antifungal properties. The extracted EO was encapsulated into chitosan NPs in the form of a nanogel by a two-step process (droplets formation and freezing). The encapsulation of the EO into nanogels enhanced its thermal stability and anticancer properties against the KB and A549 cancer cells, with IC_50_ values of 5.6 and 6.71 µg/ml, respectively [352]. More recently, Panyaja et al*.* distillated *Z. ottensii* rhizomes, extracting *Zingiber ottensii* essential oil with high anticancer activity towards both A549, MCF-7, HeLa, and K562 cancer cells, inducing cell cycle arrest at sub-G1 phase and apoptosis. However, its encapsulation into several nanoformulations, especially nano and micro emulsions (IC_50_ of 1.08 ± 2.58 and 0.74 ± 0.45 ng/mL, respectively), increased the EO toxicity, due to their retardation effects on the EO release [353].

**Fig. 20:**
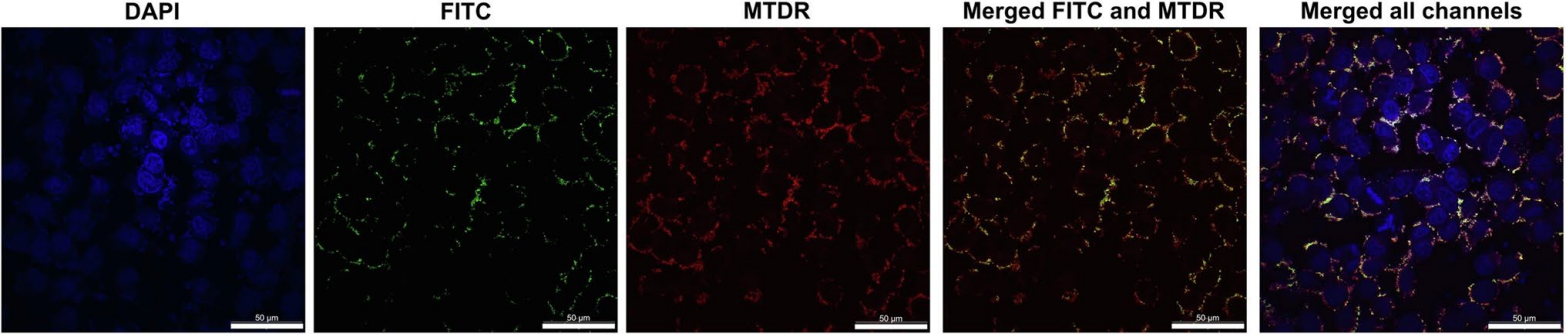
Confocal laser scanning microscopic images of SKOV3 cells after treatment of the nanosystem loaded with coumarin-6-loaded. Red signal stems from MitoTracker™ Deep Red (MTDR)-staining mitochondria. The yellow signal observed in the merged channels indicates an overlapping of FITC and MTDR channels from the NPs and mitochondria, respectively, suggesting that triphenylphosphonium acted as a mitochondrial targeting ligand. Reprinted with permission [355]. Copyright, Elsevier B.V

Plant extracts have also been encapsulated into NPs to increase their cancer therapeutic properties. Recently, Teaima et al*.* loaded SLNs with pomegranate extract, known to have high amounts of as flavonoids (e.g., catechins, anthocyanins) and hydrolysable tannins (punicalagin, punicalin, pedunculagin, gallic acid, and ellagic acid), and to present antioxidant and antiproliferative properties. The SLNs loaded with the with pomegranate extract were afterwards converted into a transdermal emulgel using Carbopol 940 as a gelling agent, and propylene glycol and ethanol as penetration enhancers. While ex vivo permeation studies of the emulgel through mouse skin indicated that the loaded SLNs transdermal delivery followed non-Fickian diffusion transport, in vivo studies concluded that the emulgel significantly reduced Ehrlich ascites carcinoma (EAC) tumor volume, with high amounts of necrotic areas in the tumor area [354]. Also, Damrongrak et al*.* produced poly(glycerol adipate) NPs decorated with FA and triphenylphosphonium for tumor and mitochondria targeting, respectively, and loaded this nanosystem with *Annona muricata* Linn leaf extract, known to have antitumor properties towards leukemia, breast and lung cancer. The loaded nanosystem entered SKOV3 ovarian cancer cells by folate receptor-mediated endocytosis and electrostatic interaction between cationic triphenylphosphonium and the negative charge of the cell membrane, targeting the mitochondria as well (Fig. 20), presenting increased cytotoxicity properties against SKOV3 ovarian cancer cells compared to free extract [355].

### Biosynthesis using plant extracts

The typical reducing agents used during NPs production, such as vitamin c, NaBH_4_ and citric acid, formaldehyde, among others, can be expensive corrosive, toxic, flammable, and irritant [356–358]. Besides, several compounds used for NPs stabilization are also toxic in high concentrations, with negative effects in the environment and the human health. However, the use of biological materials, such as plant components, has been presented as an effective, cheaper, and green alternative to produce NPs, by reducing the metallic ions to stable NPs. The green synthesis of NPs using plant extracts, biomass or algae also permits a better control of the NPs morphology and size, since the phytochemicals present in the extracts act as a natural NPs stabilizer and coating agents [356, 358]. The bioreduction of metallic ions takes place intracellularly, in which the plant gathers metal ions, or extracellularly, using crude biomass. The functional groups of plant flavonoids, alkaloids, terpenoids, polysaccharides, enzymes, protein, vitamins, and organic acids act as reductants and donate electrons to reduce metallic ions to metal complexes, resulting in the production of metal NPs [356, 357, 359]. Furthermore, several phytocompounds, such as phenols, flavonoids and terpenoids may also have active properties, such as antioxidant potential [358–360], playing a role in free radicals scavenging, while synthetic antioxidants can cause several side effect, like allergic reactions, and carcinogenic affects [358].

Several plant constituents of *Aloe vera*, *Coriandum sativum*, *Eucalyptus*, *Cinnamomum*, *Mangifera indica*, *Pinus resinosa*, *Cinnamom zeylanicum*, *Boswellia ovalifoliolata*, *Jatropha curcas*, *Syzygium cumini*, among other, as well as green and brown algae (e.g., *Saragassum natans*, *Pseudochlorococcum typicum*, *Chlorella vulgaris*, *Chlmydomonas reinhardtii*, *Turbiaria conoides*, etc.) have presented potential to reduce Au, Ag, Fe, Ti, Zn, Pd, Pt, Cu, and Cd [356, 358].

Overall, bioreduction phases are activation/nucleation, growth/development, and termination/capping. Activation consists in the reduction of metal ions and synthesis of all nuclei. During growth, the thermodynamic stability increases by the accumulation of small NPs whereas the termination phase, which ultimately shapes the synthesized metallic NPs, is the capping of the developed nuclei to prevent the NPs agglomeration [356, 357]. Another mechanism that explains biosynthesis consists in reduction, chelation, and stabilization of NPs, with the functional groups derived from the plant’s compounds. After the reduction of metallic ions, the resulting molecules coordinate with each other and chelate, resulting in a metallic core that is afterwards covered and stabilized by plant secondary metabolites [357]. FTIR has shown to be a useful tool to characterize the functional groups in the NPs, such as alcohols, C–H stretching of methylene group, –C = C– stretching and C = O stretching, –C–H bending vibration of alkanes, –C–O stretching,–C–O–C– vibration, and aromatic C–H bending [358].

**Fig. 21:**
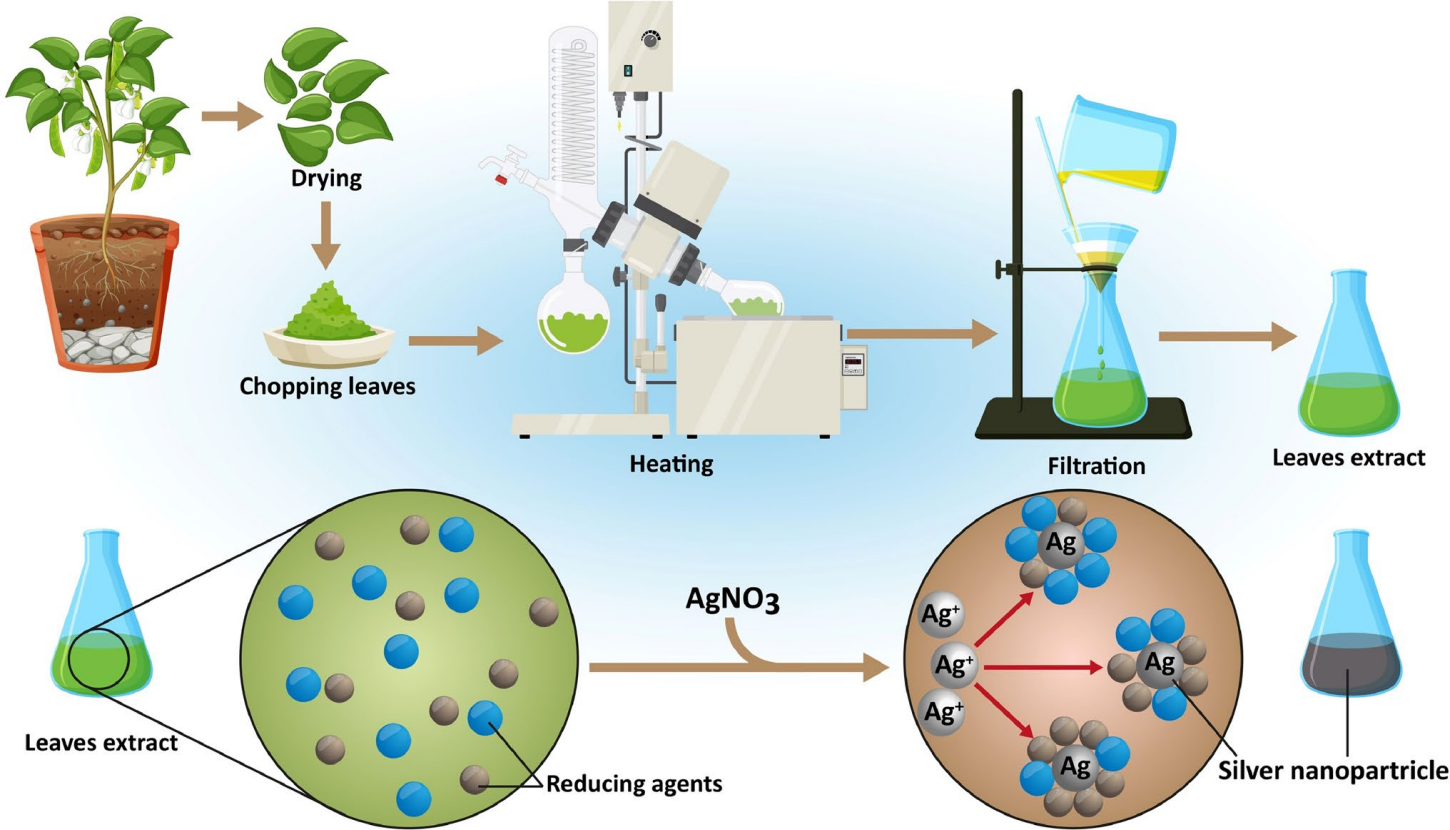
Biosynthesis of AgNPs. The plant extracts, collected by heating, contained several reducing agents, used for AgNPs biofabrication. Reproduced with permission [359]. Copyright 2022, Elsevier B.V

Several biogenic NPs have presented anticancer properties, such as CuNPs and AgNPs, which presented cytotoxicity towards MCF-7 breast cancer cells [358]. AgNPs produced by green synthesis have presented therapeutic properties for cancer therapy [357, 361]. During the biosynthesis of Ag NPs, their size and shape is mostly controlled by the environment pH (being 7 considered the optimal), temperature (between 25ºC and 40ºC), the silver salt concentration (0.25–10 mM), and the plant parts used (Fig. 21). AgNPs have presented pH-responsive behavior and their potential to increase the formation of intracellular ROS and the p53 upregulation, resulting in sub-G1 cell arrest in cancer cells, and inhibiting the formation of new blood microvessels, by reducing VEGF activity through Src and PI3K/Akt dependent pathways [357, 359, 361].

Recently, Aboyewa et al*.* produced biogenic AuNPs using mangiferin (MGF) and water extracts of *Cyclopia intermedia*. Mangiferin is a natural polyphenol found with chemotherapeutic properties, such as the ability to reverse drug resistance, and is present in several plant species, such as *Cyclopia intermedia*, an endemic species of South Africa known to have antioxidant, antimicrobial, antidiabetic, and anticancer properties. The biogenic AuNPs were loaded with doxorubicin (DOX), resulting in synergic anticancer properties against Caco-2 cancer cells, while not presenting toxicity towards non-cancer skin fibroblasts. The loaded AuNPs reduced ATP levels and increased ROS production, leading to mitochondrial depolarization, DNA fragmentation and cell apoptosis [362]. Further studies also presented the green production of magnetic iron oxide NPs from iron (III) chloride by using *Brassica oleracea var. capitata sub.var*. rubra, an autumn plant with phenolic compounds, flavonoids, glucosinolates, sulforaphane, ascorbic acids, and anthocyanin pigments. The resulting NPs presented toxicity and anti-migrative properties towards MCF-7 breast cancer cells [360]. *Acacia falcata* leaf extracts were also used for production of hydroxyapatite nanoparticles with anticancer properties towards A549 lung and MDA-MB231 breast carcinoma cell lines, with an IC_50_ of 55 µg/mL, by inducing alterations in the cell membrane and cell morphology, chromatin condensation and consequent cell necrosis [363].

## Marine organisms metabolites-loaded NM

Currently, lots of the chemotherapeutic drugs come from nature, especially from the land, such as paclitaxel, curcumin, hydroxycamptothecin, in the market. In contrast, the ocean cover around 70% area of the earth, but less than 5% deep ocean has been explored, which means the unexplored ocean and marine organisms have incalculable possibilities in bioactive products, especially in chemotherapeutic drugs [364]. Moreover, the different environment of ocean, such as pressure, salinity, temperatures, could induce the marine organisms generating secondary metabolites with extraordinary chemical and pharmacological properties [365].

The caribbean sponge is the first identified marine organisms for detailly chemical investigations [366]. After that, various secondary metabolites of marine organisms, such as algae, sponges, molluscs, bryozoa, shark cartilage, actinomyces, tunicates, have been investigated for cancer treatments [367]. Some of these marine secondary metabolites have been approved in clinal [368]. For example, lurbinectedin has been approved in treating metastatic small cell lung cancer in clinical; As a ligand of B cell maturation antigen, belantamab mafodotin has been developed as a targeted therapeutic agent in treating relapsed/refractory multiple myeloma; enfortumab vedotin has been approved in treating advanced or metastatic urothelial carcinoma [369]; As an antibody drug conjugate, polatuzumab vedotin has been applied in treating relapsed or refractory diffuse large B-cell lymphoma [370]. Specifically, these marine secondary metabolites are divided into different chemical classes, such as peptides, polyketides, alkaloids, and polyphenol [371]. Encouraged by the successful application of marine secondary metabolites in clinical, researchers are attempting to fabricated marine metabolites-loaded nanoparticles for cancer treatment since the nanoparticle platforms exhibit unique properties in drug delivery, such as improved stability, prolonged circulation time, controlled drug release, reduced potential drug side effects, etc. [372]. Herein, we will introduce some marine metabolites and the marine metabolites-loaded nanoparticles in treating cancers, in terms of the chemical classes of these marine metabolites.

### Peptides

Cryptophycins is highly cytotoxic macrocyclic depsipeptides isolated from cyanobacteria. Cryptophycins can interact with tubulin, disturbing microtubule dynamics and preventing microtubules from forming correct mitotic spindles. Consequently, cryptophycins would cause cell-cycle arrest and apoptosis. Additionally, the biological activity of cryptophycins is not influenced by the P-glycoprotein, a drug efflux system commonly found in multidrug-resistant cancer cell lines and solid tumors. Moreover, the antiproliferative effects of cryptophycins is 100 times than the paclitaxel, according to the report. Currently, there is limited report about the cryptophycins-loaded nanoparticle for cancer treatment. But the cryptophycins was covalently connected with the cyclopeptide c (Arg-Gly-Asp fK), targeting integrin αvβ3, across the protease-cleavable Val-Cit linker and two different self-immolative spacers to prepare the antibody–drug conjugates (ADCs) by Adina et al*.* [373]*.* Furthermore, the ADCs exhibited powerful inhibited effect on the proliferation of M21 and M21-L human melanoma cells.

Kahalalides is a cyclic depsipeptide with a long linear lipopeptide moiety. Additionally, Kahalalides exhibits effectiveness on various solid tumor types, which could be attributed to the deactivation of apoptosis control pathways such as AKT (protein kinase B) and receptor tyrosine kinase (ErbB/Her) causing necrosis or oncosis. Specifically, Leticia et al*.* conjugated Kahalalides with gold NPs with hydrodynamic size at 20 nm and 40 nm respectively [374]. They proved the cytotoxicity of Kahalalides-loaded gold NPs on HeLa cells by WST-1 assay. Additionally, the Kahalalides-loaded gold NPs with 40 nm exhibited the higher tangibility to cell lysosomes compared to that with size at 20 nm.

Aplidine is isolated from the Mediterranean tunicate *Aplidium albicans*. Aplidine can induce the early oxidative stress and cause a rapid and persistent activation of JNK and MAPK phosphorylation that regulate cell cycle progression. Furthermore, the activation of these pathways can trigger cytochrome c release and subsequently activate caspase cascades. Pau et al*.* prepared a aplidine loaded-glutathione degradable polyurethane-polyurea NPs modified with a cyclic RGD peptide as a targeting moiety. The aplidine-loaded NPs showed the IC_50_ (9.7 ± 2.7 nM) at MDA-MB-231 compared to the free aplidine with IC_50_ (22.1 ± 7.4 nM) [375].

Dolastatin 10 is a unique pentapeptide that isolated from the sea hare *Dolabella auricularia*. Similar as the cryptophycins, Dolastatin 10 is a potent antimitotic peptide that can inhibit tubulin polymerization. Monomethyl auristatin E (MMAE), as a synthetic analog of the natural product dolastatin 10, has been involved in prepraing various NPs. Hanhee et al*.* first covalently conjugated cathepsin B-specific cleavable FRRG peptide with MMAE via one-step simple synthetic chemistry [376]. Then, the conjugated molecules can self-assemble into about 200 nm-sized spherical NPs in aqueous condition. The NPs exhibited high stability in mouse plasma and powerful antitumor effect on the 4T1 tumor-bearing mice models.

Different from potential antitumor mechanism of the abovementioned peptides, AE-941 is isolated from the shark cartilage with the capabilities in inhibiting the angiogenesis and inducing the endothelial cell apoptosis [377]. Similar as the cryptophycins, there is limited report about AE-941-loaded NPs for the cancer treatment. But the capabilities of AE-941 in inhibited tumor development have been confirmed by some research.

### Alkaloid

ET-743 (Trabectedin) is first isolated from the *Caribbean Ecteinascidia* turbinate, it can bind to the N2 position of guanines in the minor groove of DNA and affect transcription in a gene and promoter-specific fashion. Consequently, ET-743 could induce cancer cell death and modify the tumor microenvironment by reducing the number of tumor-associated macrophages. However, the ET-743 could cause serious local toxicity in the peripheral vein. Therefore, Umberto et al*.* prepared the ET-743-loaded amphiphilic biodegradable block-copolymers nanoparticle to improve the pharmacological properties of ET-743 [378].

### Polyketide

Salinosporamide A (Marizomib) is produced by the obligate marine bacteria *Salinispora tropica* and *Salinispora Arenicola*. Marizomib can inhibit proteasome activity by covalently modifying the active site threonine residues of the 20S proteasome, leading to the cancer cell death [379]. Inspired by these properties, Lulu et al. encapsulated Marizomib (MARI) in poly (lactic-co-glycolic acid) (PLGA) NPs (MARI@PLGA-NPs). The MARI@PLGA-NPs exhibited significant cytotoxicity on two types of hepatocellular carcinoma cancer cells (Caco-2 and HepG2) [380].

## Clinical status and challenges for the clinical use of natural compounds-loaded nanocarriers

Targeted delivery and enhanced apoptosis in tumour cells of natural therapeutic agents encapsulated in nanocarriers have been widely demonstrated in several in vitro and in vivo studies, as stated above. However, despite their efficacy and the great interest of the scientific community, there are still limited clinical trials performed for the application of natural compounds loaded in nanocarriers for cancer prevention and treatment. Most trials have been conducted using the natural compounds alone or in combination with another therapeutic agents.

Regarding the application of nanocarriers for natural compound delivery in cancer treatment, a non-randomized, early Phase I clinical study using luteolin NPs were conducted to evaluate their anticancer effects on tongue squamous cell carcinoma in 4 patients (NCT03288298). Albumin NPs-bound paclitaxel (nab-Paclitaxel) associated with gemcitabine have been approved by The Food and Drug Administration (FDA) for the treatment of pancreatic cancer. Non-randomized, open-label, multi-center, Phase II safety and efficacy studies with nab-Paclitaxel plus gemcitabine in 107 patients with 18 years old or older showed that this nanomedicine was well tolerated by locally advanced pancreatic cancer (NCT02301143). In a double-blind, placebo-controlled trial with 26 muscle-invasive bladder cancer patients, the administration of 180 mg/day of nanocurcumin or placebo during the chemotherapy showed that although the nanocurcumin was well tolerated, there was no significant differences between the two groups associated with the grade 3/4 renal and with the occurrence of hematological toxicities and hematoxicological nadirs [381].

Although the nanotechnology has emerged as a promise to improve the bioavailability of the natural compounds and circumvent the most limitations associated with the conventional cancer therapy, the application of optimized nanoformulations faces various challenges required for the pre-clinical and clinical trials, such as production at an industrial level, assurance of nanotherapeutics long-term stability, physiological barriers and safety and regulatory issues.

One of the prerequisites for the insertion of nanotherapeutics into the pharmaceutical market is the translation of the laboratorial scale production in a large-scale, making it cost-competitive and safety. Nanomedicines are complex and sophisticated formulations which their clinical application are strictly dependent on their physicochemical properties (particle size, surface charge, morphology, and hydrophobicity) [382].

The reproducibility and the product analysis are essential parameters for the commercialization of nanotherapeutics, especially due to the possible dramatic changes in the physicochemical characteristics of nanomaterials during the production at large-scale. Therefore, since most of nanomaterials that are tested in vitro and in vivo studies are produced in minor batches, the physicochemical characterization of nanomedicines must be monitored after scale-up synthesis and under clinically relevant environments (certain pH and ionic strength) using several analytical methods to guarantee the same properties, stability, and biological performance. In this sense, new quality control methods must be developed and performed to characterize not only the physicochemical properties of nanomedicines, but also to evaluate their fate and behavior regarding the drug release, biodistribution and toxicity [383].

When nanomaterials are introduced into living organisms, they suffer remarkable differences in the pharmacokinetic processes, such as absorption, distribution, agglomeration, metabolism, and elimination, which can influence the pharmacological activity and toxicological profile of nanomaterials. Thus, since each nanoparticle can have its own pharmacokinetic properties, which are dependent on its physicochemical characteristics, it is a herculean task to predict the in vivo performance trends of different types of nanomaterials. A recent article published by Kumar et al*.* (2023) [384] provided a quantitative data based on the calculation of nanoparticle biodistribution coefficient from a total of 2018 published pharmacokinetics of different NPs in various mice tissues, which can be profitable for the prediction of tissue distribution of several nanomaterials that are widely used in medicine [384].

In fact, the small particle size and high surface area make nanomaterials suitable to penetrate the biological barriers and reach the desired target. However, these characteristics can also lead to several adverse effects even when nanomaterials are produced from safe compounds. Although there are numerous in vitro and in vivo studies in different organisms and cells relating the toxicity of nanomaterials with their physicochemical properties [385–388], most of them are divergent or ambiguous due to the observation of the toxicity and the absence of hazardous effects for the same nature of nanomaterials, and thus, this pattern can also restrict the transition of nanotherapeutics from lab to clinical settings.

Another limitation that may hamper the development of nanomedicines for clinical application is the lack of regulatory guidance for nanotherapeutics. As described above, due to their distinctive properties, the pharmacokinetic and pharmacodynamic profiles of nanotherapeutics are different compared with the other conventional therapeutic agents and, thus, it is extremely difficult to stablish specific guidelines to evaluate and regulate these products. Although great efforts have been made by the international regulatory agencies to create specific standard protocols to characterize these nanotherapeutics regarding the physicochemical, biological, and physiological properties, nanomedicines continue to be evaluated as other traditional drugs [389]. In this context, the quality control of generic nanomedicines (nanosimilars) will also face significant challenges since these products must have the same bioequivalence, safety and efficacy when compared to innovator nanotherapeutic and any minor changes in manufacturing can have a drastic impact on their efficiency and toxicological profile, as observed for the nanosimilar of approved nab-paclitaxel product [390].

## A bibliometric analysis on studies of natural compounds-based nanomedicine applications on cancer

In the context of natural compounds-based nanomedicine for cancer treatment, bibliometric studies provide valuable insights into the research trends and advancements in this burgeoning field. They help in identifying the most impactful studies, prominent researchers, and leading institutions, thereby offering a comprehensive overview of the current state of research. Such analysis is crucial for understanding how natural compounds are being integrated into nanotechnological frameworks to enhance their therapeutic efficacy and for tracking the progress of these innovations from the laboratory to clinical applications.

Despite the limited availability of resources analyzing clinical trials on these natural compound nanomedicines, this review aims to summarize recent studies that offer a comprehensive overview of progress in the field of natural compound-based nanomedicines and clinical trials. A study by Bhandari et al. (2022) [391] provided an extensive bibliometric analysis of biogenic gold and silver nanoparticles used in cancer therapy. It highlighted how these nanoparticles, synthesized using eco-friendly methods involving plant-based agents, offer remarkable characteristics for cancer diagnosis and treatment. The analysis covered the publication trends from 2010 to 2022, emphasizing the increasing interest in biogenic approaches to nanoparticle synthesis. Despite the numerous published reports demonstrating the benefits of these eco-friendly nanoparticles, their clinical translation remains limited. This study also discussed the specific challenges faced in bringing biogenic nanodrugs from the laboratory to the market, such as issues related to large-scale production and regulatory approvals. The review underscored the necessity for continued research to overcome these obstacles and realize the full potential of biogenic nanoparticles in clinical settings.

Furthermore, a bibliometric analysis of herbal nanoparticles, as presented elsewhere [392], underscored the significant rise in research combining herbal medicine with nanotechnology over the past two decades. The analysis used tools like VOSviewer and CiteSpace to map the landscape of this research area, identifying key trends, influential authors, and collaborative networks. It highlighted how nanoparticles derived from herbal sources can enhance the stability, bioavailability, and therapeutic efficacy of natural compounds. This study also illustrated the dynamic growth in publications related to herbal nanoparticles, particularly noting the rapid increase in research output since 2017. By identifying the most influential studies and authors, this analysis provided a roadmap for future research and highlights the collaborative efforts driving this field forward.

**Fig. 22:**
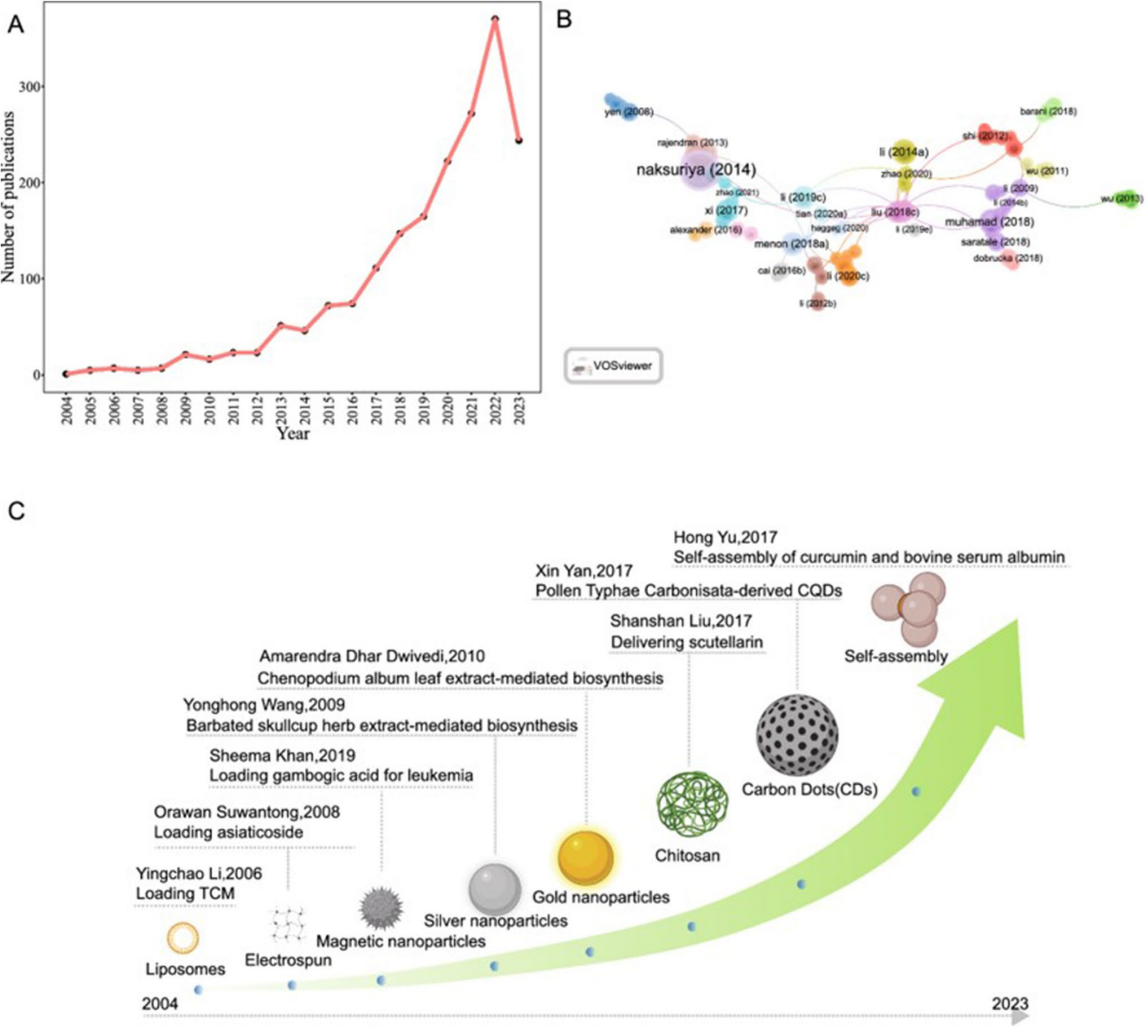
Analysis of all publications. **A** Trends in annual publications. **B** Co-citation network analysis of publications. **C** Chronological timeline of key articles in herbal nanoparticle research [392]. This is an open access article distributed under the terms of the Creative Commons CC-BY license, which permits unrestricted use, distribution, and reproduction in any medium, provided the original work is properly cited (https://creativecommons.org/licenses/by/4.0/)

The analysis included a total of 1876 publications from the WoSCC database, comprising 1512 articles and 364 reviews. A line chart (Fig. 22A) illustrates the evolution of publications related to herbal nanoparticles from 2004 to 2023, which is divided into three phases: a stable period (2004–2008), a slow growth period (2009–2016), and a rapid growth period (2017–2023). According to VOSviewer’s co-citation network analysis (Fig. 22B), the most cited article was by Eileen White titled “Curcumin nanoformulations: a review of pharmaceutical properties and preclinical studies and clinical data related to cancer treatment” [393] which was published in the Biomaterials journal (IF = 14.0) in 2014 and accumulated a total of 630 citations. The second most cited article was by Bonifacio on nanotechnology-based drug delivery systems and herbal medicines, published in the International Journal of Nanomedicine in 2014 [394]. By systematically assessing citation counts, pivotal papers on the applications of specific nanotechnologies in traditional herbal medicine were identified. A chronological timeline (Fig. 22C) highlights some milestone studies sequentially.

While bibliometric studies offer an in-depth analysis of progress and trends in natural compound-based nanomedicines, clinical trials remain relatively scarce. This study on herbal nanoparticles, for instance, highlights a significant body of research but identifies only a few clinical trials, underscoring a gap between preclinical research and clinical application. Other reviews also corroborate this, emphasizing the need for more robust clinical trials to validate the safety and efficacy of these nanomedicines [395–397]. Despite, the promising potential of natural compounds-based nanomedicine for cancer therapy, there is a critical need for more extensive clinical trials to bridge the gap between research and clinical practice. Addressing this gap is essential to fully realize the therapeutic potential of these innovative treatments.

## Conclusions and future directions

The application of nanoformulations emerges as a promising strategy in the pharmaceutical field for overcoming the limitations in the conventional therapy in cancer treatments. In this context, due to remarkable properties, nanomaterials containing natural compounds can specifically target tumor cells, improving the specificity and efficacy of cancer therapeutics which in turn improves patient compliance. This review summarized several types of nanocarriers serving as host for natural compounds from diverse origins, as well as their pivotal role for targeting cancer cells. In general, nanomedicines are complex and refined drug delivery systems and thus, before their approval from in clinical trials and marketing, their development must overcome several hurdles. These include the adaptation from the laboratorial scale production to a large-scale, with reproducible physicochemical analysis and suitable pharmacokinetics and pharmacodynamic profiles. Since each nanoformulation presents own intrinsic properties (size, surface charge, solubility, homogeneity), it is difficult to predict its behavior in vivo. Additionally, as considered above, due to their distinctive characteristics, the pharmacokinetic and pharmacodynamic profiles of nanoformulations are different compared with the other conventional therapeutic agents and, thus, it is extremely arduous to install specific guidelines to regulate such products. In this sense, the cooperation between pharmaceutical companies, research centers and regulatory authorities is urgent need for the development of effective and safe treatments based on nanomedicines for cancer therapy.

## Data Availability

Data sharing not applicable to this article as no data set was generated or analyzed during the current study.
